# Co-creation methods for public health research — characteristics, benefits, and challenges: a Health CASCADE scoping review

**DOI:** 10.1186/s12874-025-02514-4

**Published:** 2025-03-06

**Authors:** Danielle Marie Agnello, Vinayak Anand-Kumar, Qingfan An, Janneke de Boer, Lea Rahel Delfmann, Giuliana Raffaella Longworth, Quentin Loisel, Lauren McCaffrey, Artur Steiner, Sebastien Chastin

**Affiliations:** 1 School of Health and Life Sciences, Glasgow Caledonian University, Cowcaddens Road, Glasgow, Scotland G4 0BA UK; 2 School of Business, Social and Decision Sciences, Constructor University, Bremen, Germany; 3 Department of Community Medicine and Rehabilitation, Umeå University, Umeå, Sweden; 4 Department of Public Health and Primary Care, Ghent University, Ghent, Belgium; 5 Department of Movement and Sports Sciences, Ghent University, Ghent, Belgium; 6 Department of Sport Sciences, Faculty of Psychology Education and Sport Sciences Blanquerna, Ramon Llull University, Barcelona, Spain; 7 Yunus Centre for Social Business and Health, Glasgow Caledonian University, Glasgow, Scotland UK

**Keywords:** Co-creation, Co-design, Co-production, Participatory, Methods, Scoping review, Public health

## Abstract

**Background:**

Co-creation engages diverse stakeholders, including marginalized populations, in collaborative problem-solving to enhance engagement and develop contextually appropriate solutions. It is increasingly recognized as a way to democratize research and improve the impact of interventions, services, and policies. However, the lack of synthesized evidence on co-creation methods limits methodological rigor and the establishment of best practices. This review aimed to identify co-creation methods in academic literature and analyze their characteristics, target groups, and associated benefits and challenges.

**Methods:**

This scoping review follows the Preferred Reporting Items for Systematic Reviews and Meta-Analyses Extension for Scoping Reviews. The search was conducted in the Health CASCADE database v1.5 (including CINAHL, PubMed, and 17 additional databases via ProQuest) from January 1970 to March 2022. Data was aggregated and summarized, with qualitative data analyzed using Braun and Clarke’s six-phase thematic analysis approach.

**Results:**

The review included 266 articles, identifying 248 distinct co-creation methods published between 1998 and 2022. Most methods were rooted in participatory paradigms (147 methods), with 49 methods derived from co-approaches like co-creation, co-design, and co-production, and 11 from community-based health promotion and action research. Methods were applied across 40 target populations, including children, adults, and marginalized groups. Many methods (62.3%) were delivered face-to-face, with 40 articles incorporating digital tools. Thematic analysis revealed nine benefits, such as enhanced creativity, empowerment, and improved communication, and six challenges, including resource constraints and systemic and structural barriers.

**Conclusion:**

This review emphasizes the importance of robust documentation and analysis of co-creation methods to inform their application in public health. Findings support the development of collaborative co-creation processes that are responsive to the needs of diverse populations, thereby enhancing the overall effectiveness and cultural sensitivity of the outcomes. This review highlights the potential of co-creation methods to promote equity and inclusion while emphasizing the importance of evaluating and selecting methods tailored to specific objectives, offering a critical resource for planning, conducting, and evaluating co-creation projects.

**Supplementary Information:**

The online version contains supplementary material available at 10.1186/s12874-025-02514-4.

## Background

The lack of transparency, inadequate communication, and insufficient engagement of communities in the research process contribute to a perception of research as an extractive and exploitative endeavor [1]. Engaging affected populations is a proven strategy for developing relevant knowledge that can adapt health interventions and policies effectively [2, 3]. However, individuals are rarely involved in research processes beyond consultative roles, limiting them to information providers rather than active co-creators [4, 5]. To address these issues, it is essential to adopt more inclusive and participatory research approaches that actively involve all relevant stakeholders in all stages of the research process [6, 7].

Co-creation is any act of collective creativity that involves a broad range of relevant and affected actors in creative problem-solving that aims to produce a desired outcome [8]. It holds promise for enhancing stakeholder engagement, ensuring interventions are tailored to specific needs and contexts, thereby enhancing their relevance, acceptance, and effectiveness [3, 9]. Unlike traditional top-down or bottom-up processes, co-creation adopts a multi-directional approach to problem-solving [10] and fosters democratic processes and knowledge production [11–13]. Co-creation is increasingly thought of as a methodology to make research and service design more inclusive, democratic, and diverse [8, 14], and aspires to create a collaboration that recognizes shared insights, works equitably, and shares power [15]. However, this promise is only true if the methods used within the co-creation process uphold and enact the co-creation principles, such as including all perspectives and skills, using a systems perspective, enabling a creative approach to research, and sharing power and decision-making [3, 9, 16, 17]. To improve health equity, co-creation needs to empower all relevant stakeholders to be engaged in an equitable way [4]. This is particularly true for marginalized groups, as they frequently experience health disparities due to systemic, social, and cultural barriers [6, 18] and are minimally involved in research efforts [4].

Despite the growing recognition of co-creation, a body of research on methods used in co-creation is lacking [8, 9, 20]. Furthermore, reports on the process of co-creation (including methods) are scarce, with most of the resources coming from the private sector [21]. For instance, recent findings by Agnello et al. identified a significant gap in the academic literature, highlighting the underreporting of participatory and creative methods in empirical co-creation research, as well as a surprising difference between methods used in academic and non-academic sources, limiting methodological rigor and the establishment of best practices [9].

### Aim

 This study aims to identify and understand co-creation methods in academic literature, focusing on their characteristics, applicability across different populations, and the reported benefits and challenges, to inform guidance on selecting suitable methods for various problems, contexts, and target groups.

### Research questions

1. What co-creation methods are described in academic literature?
2. What methodologies are these methods derived from?
3. What are the key characteristics of these methods that can enable their replication and application in other studies?
4. Which target populations have these methods been applied to?
5. What benefits and challenges are associated with the use of these co-creation methods?

## Methods

Co-creation is a broader methodology that emphasizes collective creativity and collaboration [8], this study focuses on the methods used within co-creation, which are defined by Agnello et al. is, “co-creation methods encompass a diverse range of tools, activities, approaches, and techniques strategically employed across the entirety of the co-creation process. These methods serve various purposes, including but not limited to data collection, facilitation, recruitment, reflection, data analysis, and dissemination, allowing for flexibility in achieving diverse objectives” [9].

### Search strategy

This study focused on sourcing scientific evidence available in the academic literature about co-creation methods, as method names were already sourced from empirical studies and grey literature in the recent systematic methods overview by Agnello et al. [9].

The scoping review followed the Preferred Reporting Items for Systematic Reviews and Meta-Analyses Extension for Scoping Reviews (PRISMA-ScR) [22]. To address the lack of consensus and the interchangeable use of various co-approaches, as well as their overlap with participatory research methodologies [8], a comprehensive search was conducted in the curated and peer-reviewed Health CASCADE database v1.5 from January 1970 to March 2022 [23]. This database was developed by the Health CASCADE network, which includes relevant co-creation literature from CINAHL, PubMed, and 17 additional databases accessible via ProQuest [8]. The database excludes all the following materials: Blogs, Websites, Podcasts, Biography, Conference abstracts, papers and proceedings, dissertations and theses, letter(s) to the editor, newspapers, magazines, pamphlets or brochures, speeches, lectures, or presentations, working papers, audio and video works, artistic and aesthetic works, Encyclopaedia and reference works, or essays and interviews. The search strategy included the keywords representing a method in Table 1. The search was conducted in the title only, as all abstracts contain the term ‘method.’ The search was not limited to the field of public health, as the aim was to source co-creation methods regardless of the originating field or discipline. The search strategy was piloted and tested by the lead author (DMA), with rounds of input and assessment from the senior researcher (SC), to check the appropriateness of keywords and to ensure desired studies were identified. The search was only conducted in English due to the language limitations of the study team.

**Table 1:** Keywords used to capture methods in the co-creation database

Target term	Keywords
Method	“Method” OR “ Methods” OR “Methodology” Or “Methodologies” OR “Tool” OR “Tools” OR “Techniques” OR “Technique” OR “Procedure” OR “Procedures”

Adding specific key terms representing co-creation was unnecessary in this study, as the database utilized for this review was derived from a systematic review that comprehensively collected the entire corpus of literature on co-creation [8]. For clarity, Table 2 provides the keywords used to generate the co-creation database.

**Table 2:** Keywords used to capture co-creation in the co-creation database

Target term	Keywords
Co-Creation	“co-creat*” OR “co-conception” OR "co-production" OR "public and patient involvement" OR "public participation" OR "Participatory" OR "experience based design" OR "co-design" OR "user involvement" OR "collaborative design" OR "citizen science"

The search outcomes were combined into a folder in Zotero (Corporation for Digital Scholarship) [24], and duplicates were removed. The final set of articles was exported as a RIS file and then imported into Rayyan (Qatar Computing Research Institute) a systematic review manager [25], for the title/abstract screening. Any additional duplicates found by Rayyan were also reviewed and removed when determined to be duplicates.

### Screening

The articles were double-screened in pairs by five researchers (DMA, GRL, VAK, JB, and QL) based on the selection criteria outlined in Table 3. Primary research (including protocols), methodological papers, and literature reviews were included if they fulfilled the selection criteria. Each pair screened independently, with conflicts resolved by a third reviewer.

**Table 3:** Title/Abstract selection criteria

The material is included if it fulfills all the following:	The material is excluded if it fulfills one or more of the following:
Includes at least one of the keywords used in the search (above) in the title or abstract	Does not include at least one of the keywords used in the search (above) in the title or abstract
A method(s) is mentioned by name in the title or abstract	The title or abstract does not mention/describe at least one method
The abstract provides a (brief) description of the method(s) or indicates that the study is about the development, assessment, or application of the method or methods	The abstract does not have a description of the method or is a guideline or framework paper
The title or abstract was written in English	The title or abstract was written in a non-English language
Is considered ‘co-creation,’ based on the Agnello and Loisel et al. definition of co-creation [8]	Is not co-creation

After the title and abstract screening, the included articles were exported as an RIS file and moved to the full-text screening phase. The RIS file was re-uploaded into Rayyan, and the included citations were divided among six researchers (JB, LRD, LMcC, QA, QL, and VAK). Due to the large volume of articles, each researcher single-screened their assigned packet using the selection criteria in Table 4. To ensure robustness and consistency, the lead researcher (DMA) acted as the second reviewer, double-checking the decisions. Any conflicts or uncertainties were resolved through discussion among the team members to ensure adherence to the selection criteria.

**Table 4:** Full-text selection criteria

The material is included if it fulfills all the following:	The material is excluded if it fulfills one or more of the following:
The full text is accessible	The full text is not accessible
A method(s) is mentioned by name in the full-text	The article does not mention or describe at least one method
The article provides a (brief) description of the method(s) or indicates that the study is about the development, assessment, or application of the method or methods	The article only mentions the name of the method but does not describe the method itself, provide any details about the method, or describe when or how it was used in the study (e.g., this can be a primary study)
The article is about a singular method, or methods, used in a process	The article is about a methodology (e.g., full process, model, or framework) and not about a singular method, or methods, used in a process
Is considered ‘co-creation,’ based on the Agnello and Loisel et al. definition of co-creation [8]	Is not co-creation
The full text was written in English	The full text was written in a non-English language

### Data extraction

Articles that passed the full-text screening process were reviewed in Rayyan for extraction (DMA, GRL, LMcC, LRD, JB, QA, and VAK). The data extraction form was developed by the lead author (DMA) and senior researcher (SC) to align with the research questions of this study. The form was tested on sample articles and revised accordingly.

Data was extracted per method, using the extraction form in Google Forms (Google Workspace) [26]. For instance, if an article had two methods, then two forms were completed for that article. The extraction form prompted the researcher to extract details about each method, including 1) a description of the article (e.g. article name, year, authors); 2) details about the method (e.g. name, steps, type, execution details); and 3) supportive data about the co-creation process (e.g. models or frameworks used, digital tools, methodology or theory). It is important to note that all the classifications, such as the method type and the methodological approach, were determined based on the language used in the source article. An additional file shows the extraction form (see Additional File 1).

Using the pre-defined form, the extracted data was downloaded and merged into a standard reporting form (Microsoft Excel format), which facilitated the integration and additional analysis of the findings. The extracted data were reviewed by the lead author (DMA) and any confusion or discrepancies regarding the data were discussed and resolved between the researchers.

### Analysis

Data from the extraction table were summarized for ease of reference and gathering of key insights, such as intangible outputs, execution time, facilitator need, and examples of application. Additional focus was placed on the underlying methodology, method delivery, associated digital tools, method types, and a co-occurrence analysis of method combinations.

Qualitative data extracted from the included articles, aiming to ascertain the benefits (pros) and challenges (cons) of each method, was analyzed in a manner that respected the subjectivity and perspectives of the authors of the included article, minimizing additional interpretation by the researchers and focusing on synthesizing the reported insights [27]. The data was analyzed using an inductive thematic analysis approach in NVivo version 1.7.2 (Lumivero) [28] following the six phases outlined by Braun and Clarke [29].

In line with Braun and Clarke's guidance, no pre-existing coding framework was applied, and the reported themes emerged through open coding and iterative theme development [29]. Phase 1 (familiarisation), Phase 2 (generating initial codes), and Phase 3 (generating themes) were carried out by the lead researcher (DMA), with subsequent Phases 4 (reviewing potential themes) were executed with four researchers (DMA, GRL, LRD, and VAK) to ensure accuracy and coherence. Phase 5 (defining and naming themes) and Phase 6 (producing the report) were completed by the lead researcher (DMA), and the themes were reviewed, revised, and approved by eight researchers (DMA, GRL, JB, LMcC, LRD, QA, SC, and VAK). In cases of disagreement, the issues were discussed among the co-authors until a consensus was reached.

## Results

### Search strategy and screening

**Fig. 1:**
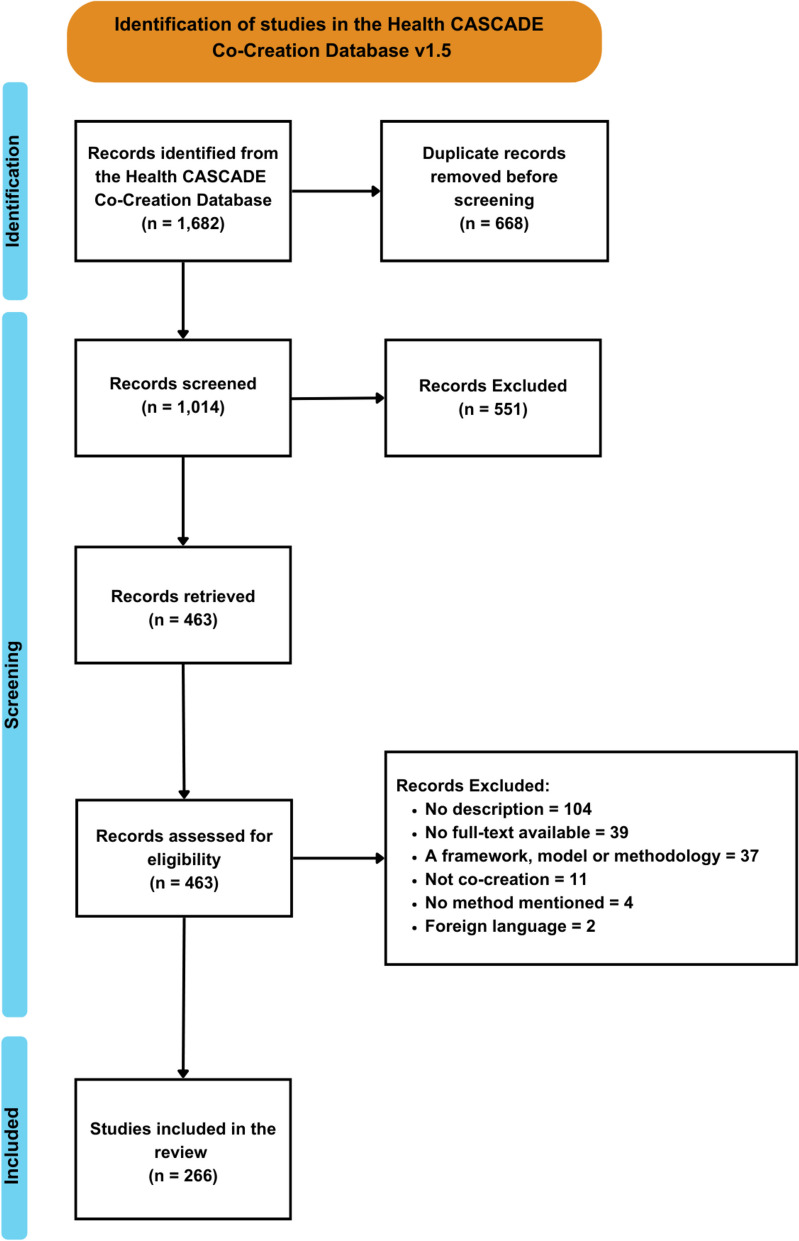
PRISMA flow chart

A total of 1,682 search results were initially retrieved from the database search. After removing 668 duplicates, 1,014 unique articles remained for further screening. After full-text screening, 266 articles were included for final analysis. Figure 1 shows this in a PRISMA flow chart, and an additional file contains the completed PRISMA-ScR checklist (see Additional File 2).

### Extracted data

**Fig. 2:**
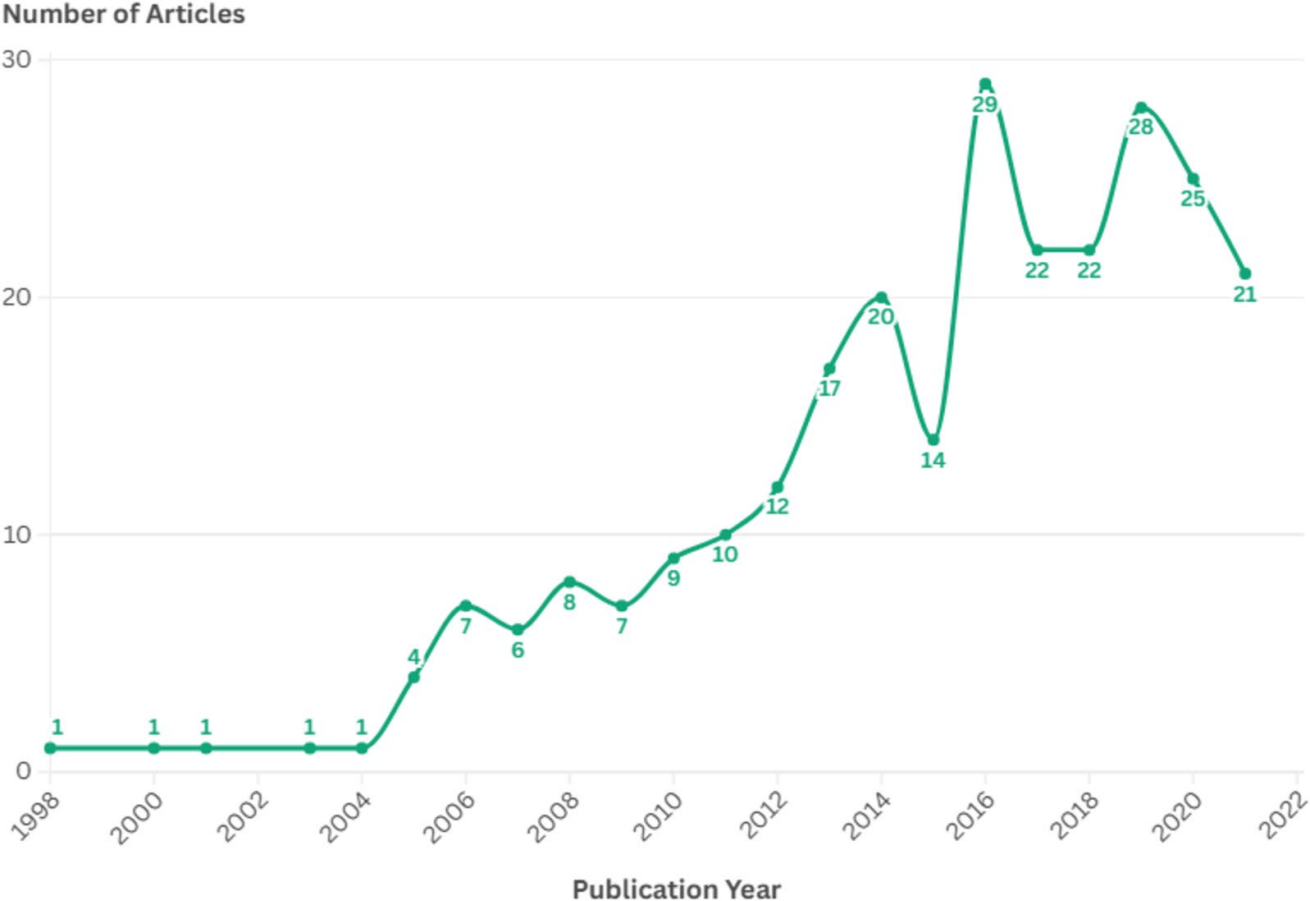
Number of included articles per year; total included articles = 266

The researchers performed 404 data extractions from the 266 included articles, gathering data on 248 different methods used in co-creation. The included publications ranged from 1998 to 2022, with most of the articles published from 2016 to 2022, which is visualized in Fig. 2.

Of the 404 extractions, 71.8% (*n* = 290) described the steps of the method (e.g. method script), 100% (*n* = 404) described the purpose of the method (what it is aiming to achieve), and 49.8% (*n* = 201) reported some intangible outputs (e.g. improved understanding, enhanced social bonds, or increased comfort and connection among participants). Furthermore, 29.7% (*n* = 128) reported expected execution time, 68.6% (*n* = 277) expressed a need for a facilitator, 87.6% (*n* = 354) gave an example of applying the method, and 51.7% (*n* = 209) gave an example of another method to use together with the reported method. Interestingly, only 24.5% (*n* = 99) described the use of a process, model, or framework, and 78.8% (*n* = 78) of those explained the steps of the process, model or framework.

Methods were used in projects that had different modes of delivery. For instance, 62.6% (*n* = 253) were delivered face-to-face, 5.7% (*n* = 23) processes were delivered online, 4.5% (*n* = 18) were delivered in a hybrid format, and 30.2% (*n* = 122) did not describe how the method was delivered. Regarding digitalization, 40 of the articles discussed an associated digital tool, which ranged from Geographic Information Systems (e.g. ArcGIS), GPS tools, Smartphones, and GoPro cameras, to Google Earth and Maps and mapping software (e.g. Mental Modeler, Netdraw, Venism Software). They also discussed the use of different Microsoft programs (e.g. Excel, PowerPoint, Word, and MS Teams) to support the method. An additional file contains the full details of the extracted data (see Additional File 3).

### Methodological underpinnings

Of the 266 included articles, 194 reported using a methodology or approach. The number of articles and their associated approach is presented in Table 5.

**Table 5:** The number of articles that contained different methodologies

Number of Articles	Type of Approach	Methodologies
144	Participatory Approach	Participatory research, Community-based participatory research, Participatory action research approach, Youth-led participatory action research, or Participatory design
30	Co-approaches	Co-creation, Co-design, and Co-production
20	Various types	Action Research, Participatory evaluation, Ethnographic approach, User-centered design, and Community participation

The following methodologies were represented by one study: Patient and public involvement, Public participation, Community-based health promotion, Formative community-based research, Interactive participatory learning, Social ecology, Community partnership in research, Patient-centered research, Community-based management, and Service design.

Furthermore, the number of methods extracted per methodology varied over time according to the publication year. Each approach also contributed a certain number of methods. Table 6 visualises the distribution of methods over time and across methodologies. An Additional File contains the extraction table, which includes methods per methodology (see Additional File 3).

**Table 6:** The number of methods sourced from different methodologies

Number of methods	Approach	Methodologies	Years
147	Participatory Approach	Participatory action research, participatory research, community-based participatory research, and participatory design	1998 to 2022
49	Co-approaches	co-creation, co-design, co-production	2008 to 2022
11	Various	Community-based health promotion and action research	2014 to 2022

### Method types

Different types of methods were extracted from the included articles based on how they were described in the source material, which are depicted in Table 7. The remaining (22 methods) were not categorized into a method type because they were not described or labeled as a specific study type in the source material.

**Table 7:** The number of methods per method type sourced from the included literature

Type of method	Number of methods
Participatory	137
Qualitative	99
Mixed methods	35
Quantitative	16
Visual	4
Semi-quantitative modeling	4
Sampling	2
Observational	1

Depending on the article it was sourced from, some methods were reported as different method types, so those methods were categorized under both method types (e.g. participatory and qualitative, or participatory and visual). For instance, different sources categorized the Photovoice as a qualitative (*n* = 17 articles) and participatory (*n* = 26 articles) method. An additional file contains the full details on methods per method type (see Additional File 4).

In 174 instances the authors reported methods that were used together in the co-creation process, providing insights into how methods can be combined. The extracted combinations were grouped by method type. The analysis of co-occurrences between source and target methods visualizes key patterns, which are displayed in Table 8.

**Table 8:** Methods Combinations (by type) and their frequency of co-occurrence

Source Method Type	Target Method Type	Frequency % (*n* = 258)
Participatory	Participatory	26.7% (*n* = 76)
Qualitative	Qualitative	18.2% (*n* = 52)
Participatory	Qualitative	17.9% (*n* = 51)
Qualitative	Participatory	6% (*n* = 17)
Mixed	Qualitative	4.9% (*n* = 14)
Quantitative	Qualitative	3.2% (*n* = 9)
Mixed	Participatory	2.5% (*n* = 7)
Mixed	Mixed	2.5% (*n* = 7)
Visual	Qualitative	2.5% (*n* = 7)
Quantitative	Participatory	2.1% (*n* = 6)
Qualitative	Mixed	1.8% (*n* = 5)
Quantitative	Mixed	1.8% (*n* = 5)
Visual	Participatory	1.4% (*n* = 4)
Visual	Visual	1.4% (*n* = 4)
Sampling	Sampling	1.4% (*n* = 4)
Qualitative	Quantitative	0.7% (*n* = 2)
Mixed	Quantitative	0.7% (*n* = 2)
Qualitative	Sampling	0.4% (*n* = 1)
Quantitative	Quantitative	0.4% (*n* = 1)
Participatory	Visual	0.4% (*n* = 1)
Visual	Quantitative	0.4% (*n* = 1)
Quantitative	Visual	0.4% (*n* = 1)
Visual	Observational	0.4% (*n* = 1)
Participatory	Observational	0.4% (*n* = 1)

These findings illustrate the varied ways methods intersect, with Participatory and Qualitative approaches dominating the landscape, while Visual and Quantitative combinations remain relatively rare. An additional file contains the full set of combined methods and a visualization of the method combinations (see Additional File 5).

### Target population

Almost half of the articles described a target population (*n* = 119). Articles reported the engagement of 40 different target populations with 139 different methods.

Engaged populations ranged from academics, healthcare professionals, and caregivers to more marginalized groups such as autistic children [30], LGBTQAI + individuals [31–33], refugees [34], people living with dementia [35, 36], disadvantaged single mothers [37], and Indigenous people [38–42]. The target population that had the most methods were Children (*n* = 33), Adults (*n* = 22), and Youth (*n* = 16). The methods used with the most target populations are Photovoice (*n* = 16), Photo-elicitation/Photographic elicitation (*n* = 6), and Alternative scenarios (*n* = 6). Table 9 depicts the methods used per target population and their associated source reference. The methods were grouped based on the language used in the study it was extracted from.

**Table 9:** Methods used per target population

Target Population	Method (Reference)
1. Academics	Partnership Data Report for Reflection [22] and World Café [23]
2. Adolescents	Art-based narrative interview [24]; The River of Life [22]; Photovoice [25, 26]; Card sort [27]; and VR FestLab [28]
3. Adults	The Three-StepTest-Interview [29]; Posters [30]; Situation analysis tool [31]; The River of Life [22]; Interview [32]; Mockups of webpages [33]; Participant observation [34]; User stories [35]; Conflict Family (cultural storytelling activity) [36]; Co-created river [37]; Multi-criteria decision analysis [38]; Photovoice [39–41]; MUST method [42]; Photo-elicitation [43]; Co-design by Appropriation of Affordances [44]; Visioning [45]; Participatory systems mapping [46]; Geo-Wiki online tool [47]; Storytelling Group [48]; Sensemaking [35]; Web-based visualization tool [49]; and Social Network Analysis [50]
4. Autistic children	Full-Body Interaction [51]
5. Cancer survivors	Photovoice [52]
6. Caregivers	Carer's Assembly [53] and Purposive sampling [54]
7. Children (under 18 years old)	About me [55]; Art Making [56]; Bookmaking [56]; Building a Model [56]; Challenge lists and asset cards [57]; Diamond ranking [58]; Digital storytelling [59, 60]; Draw and write technique [61–64]; Drawing [65]; Five Field Map [66]; FUBImethod [67]; Graphs over time [68]; Image Theatre [59]; Informal interviews [61]; Lego Serious Play [69]; Mosaic approach [63, 70]; Novelty scale: lolly jars [55]; Novelty Scales: Smiley faces [55]; Participant observation [71]; Participant Photography [72]; Participatory Theme Elicitation [73]; Participatory Video [59]; Photo-elicitation [55, 58]; Photovoice [26, 74, 75]; Puppets [63]; Role-playing [56]; Semi-participant observations [61]; Stick-a-star quiz [61]; Story board [76]; The Five Whys Method [77]; Video diary [78]; Visual voices method [79]; and Word Search [76]
8. Children with special needs	Diamond ranking [80]; School preference cards [80]; SCERTS observational checklists [80]; and The Graffiti Wall [80]
9. Community members	Asset mapping [81]; Concept mapping (aka Cognitive mapping) [82]; Dot map focus groups [83]; Partnership Data Report for Reflection [22]; Pathways [45]; Photovoice [84]; Satellite imagery-assisted activity logs [83]; World Café [23]; and Yonmenkaigi System Method [85]
10. Disabled people	Interpretative Phenomenological Analysis [86]; The Concerns Report Method [87]; and Photovoice [88]
11. Farmers	Cross sectional survey [89]; and Photovoice [90]
12. Forest communities	Alternative scenarios [45]
13. Healthcare professionals	Concept mapping (aka Cognitive mapping) [91]; Nominal group technique (expert panel) [92]; Semi-structured interview [78]; Sociogram (directed graph) [93]; World Café [23]; and User driven systematic review [94]
14. Homeless people	Photovoice [95]
15. Immigrants	Concept mapping (Cognitive mapping) [96]
16. Indigenous people	Storytelling [97]; Gaataa’aabing Visual Research Method [98]; Interpretive focus groups [99]; Digital storytelling [100]; and Group interview [101]
17. Inuit communities living with diabetes	Storytelling [102]
18. LGBTQAI +	Community mapping [103] and Photovoice [26]
19. Low-income people	Body measurements [104]; Causal loop diagram [68]; Graphs over time [68]; Snowball sampling [54]; and The Concerns Report Method [87]
20. Marginalized people	Alternative scenarios [45]; Participatory Video [105]; Mandala drawing [106]; Participant-created comics [45]; and Photovoice [107, 108]
21. Muslim women	llustrative arts-based method [109]
22. Non-binary youth	Body mapping [110]
23. Nurses	Graphic Facilitation [93]; Photographic elicitation [93]; and Sociogram (directed graph) [93]
24. Older people	Photovoice [111, 112]; Interview [32]; Alternative scenarios [45]; Living Lab [113]; Photo-elicitation [43, 114]; storyboard & animations [113]; and Life Café [115]
25. Parents	Narrative Interview [78]
26. People living with breast cancer	Critical incident technique [116]
27. People with chronic non-cancer pain	Purposive sampling [54]
28. People with Dementia	Storytelling [117] and Policy café [53]
29. People with diabetes	Nominal group technique (expert panel) [92]
30. People with low literacy	Alternative scenarios [45] and Photo-elicitation [118]
31. People with mental health challenges	Participatory mapping [119]
32. Refugees	Forum Theatre [120] and Playback Theatre [120]
33. Rural populations	Photovoice [95]
34. Single mothers	Photovoice [121]
35. Students	Co-created river [37]; Brief Re-Edited With Gamification Elements [122]; and Photovoice [41]
36. Teachers	Participatory visual methodology [123]
37. Underserved at-risk people	Photo-elicitation [43]
38. Vulnerable people	Blended approach: photovoice and photo-elicitation [124]; Daily activity space travel diary [125]; Geocaching games [83]; Listing, scoring, ranking [126]; Participant Photography [72]; Participatory geographic mapping [125]; Photo production with interviews [117]; Photovoice [62, 108, 127–131]; Analytic hierarchy process [132]; and Alternative scenarios [133]
39. Women with previous gestational diabetes	Facebook group [134]
40. Youth (15–24 years old)	Community mapping [83, 103]; Digital storytelling [59, 60]; Dot map focus groups [83]; Geocaching games [83]; Image Theatre [59]; Lego Serious Play [69]; Illustrative arts-based method [109]; Participant Photography [72]; Participatory Theme Elicitation [73]; Participatory Video [59]; Participatory/reflective photography [135]; Peer-interviewing [136]; Photovoice [39, 127, 128, 137]; Satellite imagery-assisted activity logs [83]; Wellness Quest tool [138]; and Youth ReACT (Research Actualizing Critical Thought) data analysis method [139]

### Thematic analysis

The data was analyzed using an inductive thematic analysis approach in NVivo version 1.7.2 (Lumivero) [28] following the six phases outlined by Braun and Clarke [29]. An additional file contains the codebook including the theme and code name, description, example text, and indicator terms (see Additional File 6).

### Method benefits

**Fig. 3:**
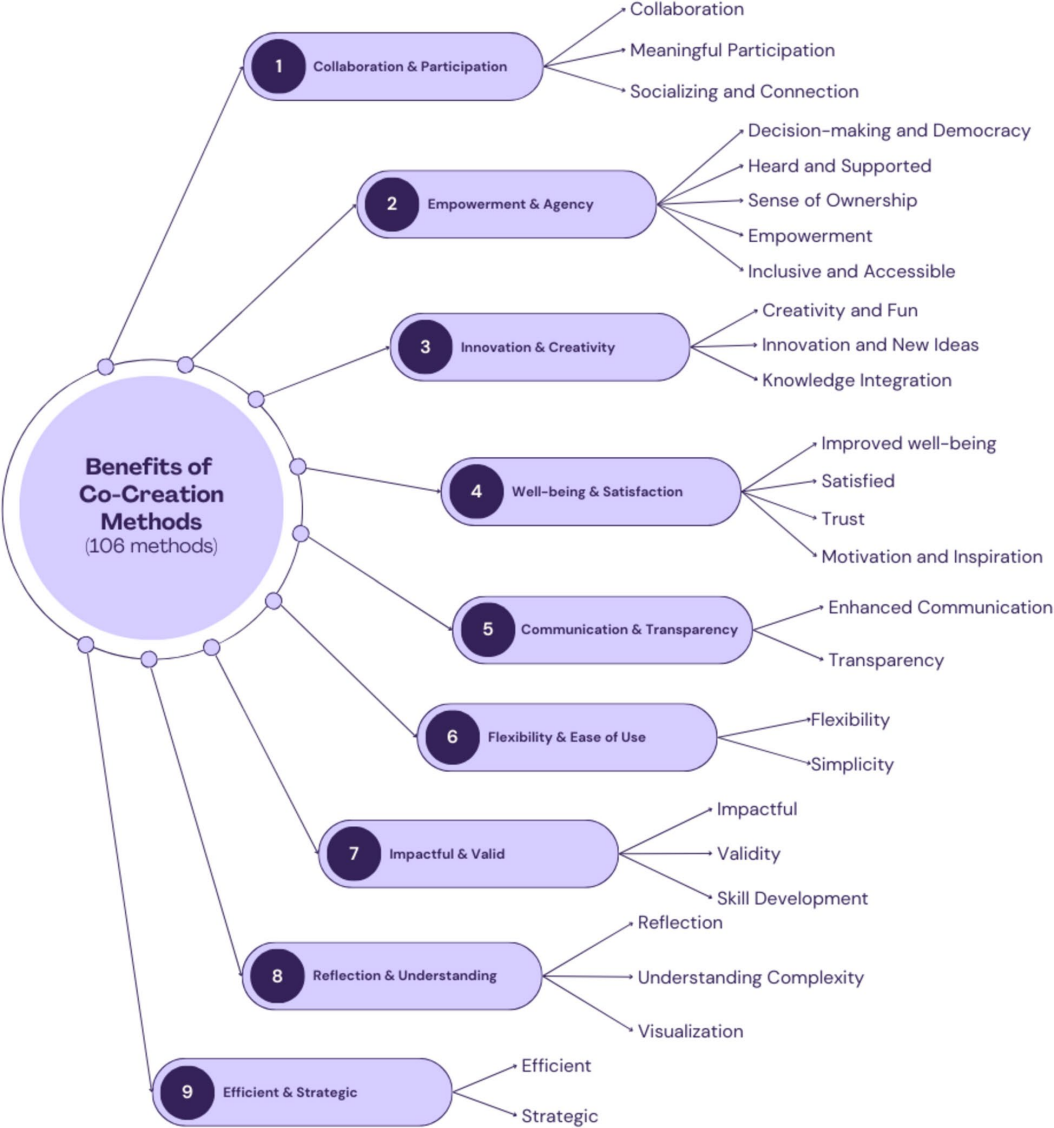
The reported benefits of 106 methods used in co-creation; including 9 themes and 27 sub-themes

**Fig. 4:**
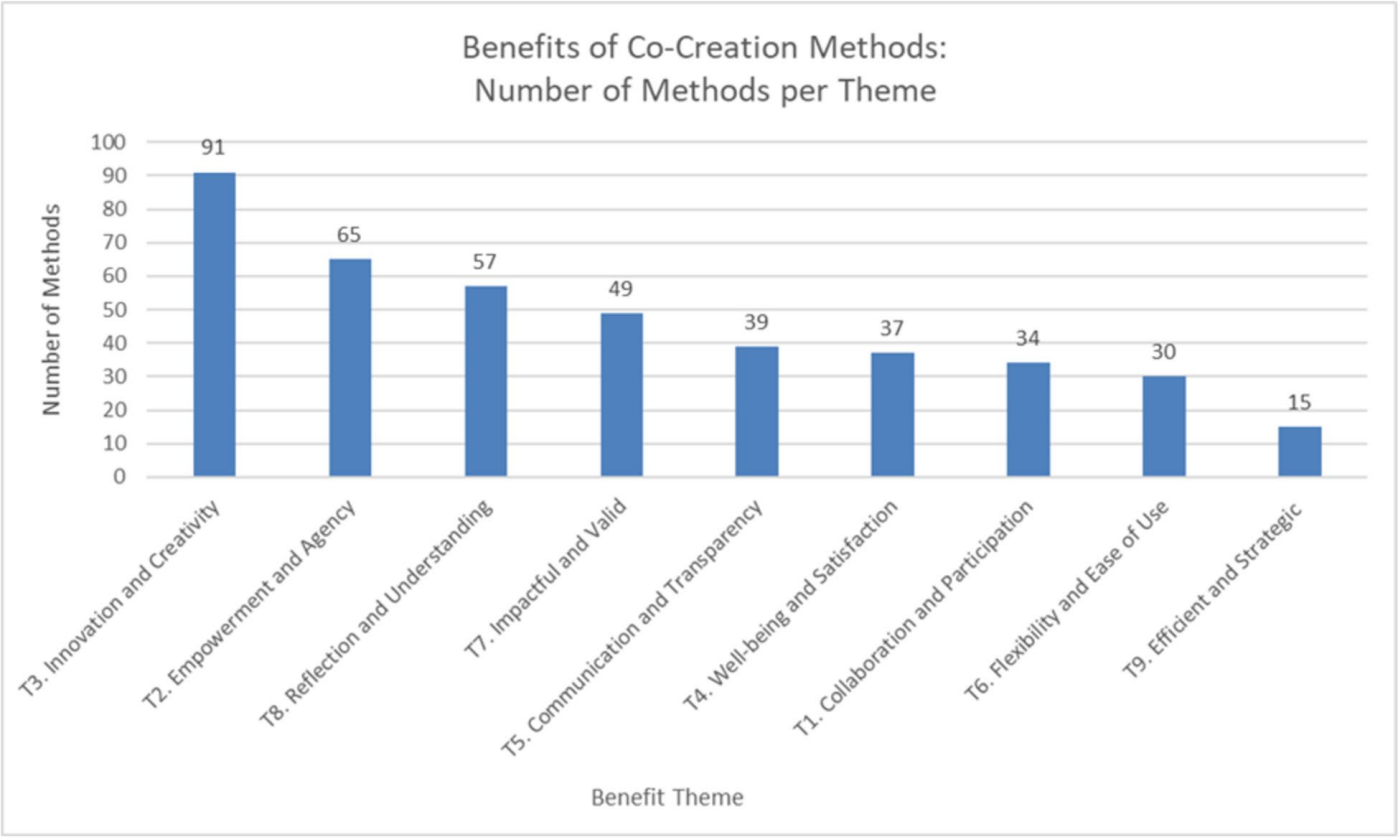
Graph of the number of methods that align with themes 1 through 9. The order of the themes is from highest to lowest number of methods. T# equals the theme number

Data regarding the benefits was extracted per method: 139 articles reported the benefits of 106 different methods, and the following is a summary of the findings grouped into 9 themes and 27 sub-themes. These themes and sub-themes are visualized in Fig. 3, and an additional file contains the set of benefits per method (see Additional File 7), and the total number of methods per theme is visualized in Fig. 4.

An additional file contains the sub-theme names, descriptions, and associated methods and their references (see Additional File 8).

#### Theme 1: collaboration and participation

Collaboration and participation are fundamental to co-creation, shaping how power, decision-making, and engagement unfold within 34 different methods. This theme emphasizes active involvement, shared ownership, and relational dynamics, reflected in the sub-themes: Collaboration, Meaningful Participation, and Socializing and Connection. These methods demand ongoing, meaningful co-creation, where participants shape the research process rather than merely contributing insights. As Barry and Higgins describes how “Photovoice promotes equal partnership and collaboration” [52] and Hartwig speaks about how “the method embraces the participatory and collaborative nature of research” [53].

Meaningful engagement is key, with O’Reilly-de Brún et al. showing how methods “have the capacity to facilitate meaningful engagement that automatically incorporates co-generation and co-analysis of data by and with stakeholders” [54]. Co-creation methods also foster connection and relationship-building, ensuring participants feel integrated. This relational aspect is reflected in studies noting how “connection can be established in the very early phase of a project, as the base for sustainable outcome” [55] and how “people will also make connections with other visions and other people” [56].

These findings reinforce that collaboration is not just about working together but about creating spaces for shared learning, trust, and connection. Co-creation methods also encourage reflection, agency over expression, and connection through shared experiences, demonstrating that knowledge production is a social, iterative process rather than a linear extraction of data.

#### Theme 2: empowerment and agency

Empowerment and inclusive decision-making are central to 65 co-creation methods, fostering democratic participation, decentralized decision-making, and reducing power imbalances. The five sub-themes (Decision-making and Democracy, Heard and Supported, Sense of Ownership, Empowerment, and Inclusive and Acessible) illustrate how these methods redistribute power, amplify participant voices, and ensure accessibility for diverse populations. At their core, these methods foster equitable participation, positioning individuals as active agents in shaping outcomes rather than passive contributors. Voinov et al. describe how Agent-based Modelling enables “decentralized, autonomous decision making” [45] and Szczepańska et al. introduced how Civic Budgeting has the potential to “promote collaborative decision-making” [107].

A key aspect of empowerment is ensuring participants feel heard and supported. Methods like Photovoice offer participants “the opportunity to share their pain experiences and feelings with others” [88], while Keogh et al. describe how Carer’s Assembly allowed participants to feel “heard and many reported, and for the first time as a family carer, they felt valued and had a voice” [35]. These methods build trust and foster inclusive dialogue, even in conflict situations.

Ownership is another vital element, allowing participants to control and influence research outcomes. O'Reilly-de Brún points out how Direct Ranking is “giving [participants] power” [54] by allowing them to define their own priorities, while Townley et al. emphasize how Participatory Mapping can allow individuals to “draw their own maps, as opposed to relying on pre-drawn maps or census boundaries” [108].

Beyond individual empowerment, many methods actively challenge power hierarchies and create equal partnerships. Townley et al. note, Participatory Mapping empowered participants to “be able to explain their communities from their own unique perspectives” [108], and Participatory Video positions participants as “equal partners alongside government authorities to provide a collaborative approach to problem solving” [109]. This redistribution of power fosters community-led engagement, promotes social justice and ensures that traditionally excluded voices play a role in decision-making.

Inclusivity and accessibility are essential components, ensuring that barriers such as literacy, language, or technical knowledge do not exclude participants. Leurs describes how participants found Checklists “easier to relate to” [110], while Linabary et al. state how participants found the Conflict Family method “flexible and accessible” [111]. O'Reilly-de Brún et al. comments on how Direct Ranking “can appeal to a wide range of stakeholder groups, including those where literacy and/or numeracy challenges” [54], helping to create safe, participatory spaces for children, marginalized communities, and hard-to-reach groups.

By redistributing power, amplifying participant voices, and promoting inclusivity, co-creation methods in this theme foster shared ownership, empowerment, and democratic decision-making processes.

#### Theme 3: innovation and creativity

Creativity and innovation are central to 91 co-creation methods, fostering imaginative thinking, knowledge integration, and the generation of new ideas. The three sub-themes (Creativity and Fun, Innovation or New Ideas, and Knowledge Integration) highlight how these methods create dynamic, engaging environments that encourage participation and problem-solving. Many co-creation methods incorporate playfulness and enjoyment, allowing participants to engage in storytelling, artistic expression, and exploratory thinking. One study describes how participants “enjoyed developing the different types of scenarios” [46] and “often had a profoundly positive effect—of collective laughter, recognition and release” [34]. This element of fun is particularly valuable when working with youth or groups disengaged in traditional research settings, helping sustain motivation and inspire spontaneous ideas.

Beyond creativity, co-creation methods stimulate innovative thinking and novel solutions. By encouraging participants to approach problems from new perspectives, they help “participants to see beyond its prior emphasis and instead led to [identifying] a wider set of intervention options” [66]. Some methods introduce non-traditional data collection and analysis techniques, such as Felker‑Kantor et al.'s description of “an innovative approach to collect activity space data” [118]. This capacity for generating new strategies makes these methods particularly valuable in co-creation.

Co-creation is not just about innovation; it also integrates diverse perspectives to tackle complex problems. Methods within this theme combine personal insights, cultural knowledge, and scientific expertise to develop cohesive project visions and actionable solutions. Wang discusses how an Art-based Narrative Interview can “access and transform [participants’] interior lives, and both invite the clients to the world of phenomenological knowing that is not easily put into words” [128]. Similarly, Timotijevic and Raats emphasizes how Citizens’ Jury includes “"hard-to-reach" residents in decision-making” [62], ensuring socially and culturally sustainable research.

By fostering playfulness, innovation, and meaningful knowledge integration, co-creation methods empower participants to think beyond conventional solutions and contribute to transformative change.

#### Theme 4: well-being and satisfaction

Well-being and satisfaction are key benefits of 37 co-creation methods, offering therapeutic effects, and creating a relaxed, anxiety-reducing environment. The four sub-themes (Improved well-being, Satisfied, Trust, and Motivation and Inspiration) highlight how these methods promote emotional release and socioemotional benefits, fostering comfortable settings for exploring sensitive topics. Fairchild and McFerran describe that co-creation can offer “therapeutic effects” [112], while Blodgetta et al. mention how Mandala Drawing “can alleviate tension and apprehension that participants might feel in the research context” [139]. This emotional support enhances engagement and allows for more open dialogue.

Participants frequently express high satisfaction, describing co-creation as a rewarding and meaningful experience. Timotijevic and Raats report that “subjective satisfaction with the events were high” [62], and Dodds et al. noted that “the participants enjoyed their involvement in the research phases” [129]. This sense of fulfillment encourages continued participation and increases the likelihood of sustained engagement. Creating safe spaces for dialogue further enhances participant well-being, as seen in Sommer et al.'s work in Tanzania, where participatory methods enabled youth to share honest descriptions of their lived experiences in spaces where they felt heard [104].

Trust is another critical outcome, fostering deeper engagement and strengthening relationships. Timotijevic and Raats note that participants “felt that the organizer was trustworthy” [62], while Vallely et al. described how Listing, Scoring, Ranking fosters “trust and understanding between researchers, study participants and community representatives” [140]. This trust-building is vital when working with sensitive topics or marginalized communities, laying the foundation for open collaboration.

Finally, co-creation methods sustain engagement by fostering motivation and inspiration, encouraging participants to take ownership and seek solutions. Beyer et al. note that participants “become motivated to seek solutions” [117], and Lahtinen et al. noted that the Future Workshop was “perceived as inspiring by the participants” [116]. Participants frequently leave co-creation processes enthusiastic and eager to continue engaging in identifying and addressing key challenges.

By enhancing emotional well-being, increasing satisfaction, building trust, and sustaining motivation, co-creation methods empower participants to engage deeply and contribute meaningfully to collaborative problem-solving.

#### Theme 5: communication and transparency

Communication and transparency are key benefits of 39 co-creation methods, enhancing stakeholder engagement through experience sharing, rapport building, and non-verbal expression. The two sub-themes (Enhanced Communication and Transparency) emphasize how these methods create spaces for participants to express themselves in ways that traditional approaches may not facilitate, fostering deeper connections and shared understanding. Dodds et al. describe how the Zaltman Metaphor Elicitation Technique is “a powerful tool to uncover both unconscious and latent thoughts and feelings that would be difficult to articulate in discursive interviews” [129], while Concept Mapping fosters “rapport between researchers and community members through a system of data collection and analysis that incorporates community input into all stages of research,” [135].

By stimulating focused dialogue and enabling participatory forms of expression, these methods ensure that diverse perspectives are shared and understood. For example, Photovoice “can be used with participants with a variety of communication needs” [102], while Participatory/Reflective Photography “captures an increasingly culturally dominant mode of human communication and self-expression” [51]. Such methods help navigate complex or controversial topics through natural, culturally significant dialogue, aligning communication with participants' lived experiences.

Transparency is equally critical, ensuring participants feel informed and involved in decision-making. Co-creation methods promote open processes, providing structured opportunities for participants to express their opinions. Evans et al. note that Alternative Scenarios support transparency by “opportunities for all participants to express their opinions in a more structured way” [46], and Participatory Modelling “increases transparency and allows reconstruction and analysis of what happened” [45]. This openness enhances trust, accountability, and inclusivity.

By improving communication, cultivating trust through transparency, and encouraging participatory expression, co-creation methods strengthen stakeholder relationships and ensure that all voices contribute meaningfully to the process.

#### Theme 6: flexibility and ease of use

Flexibility and ease of use are benefits of 30 co-creation methods, enhancing adaptability to evolving insights and allowing for easy modification across different contexts. The two sub-themes (Flexibility and Simplicity) reflect how these methods enable spontaneous application and versatility, making them particularly valuable for different settings. Sandman et al. describe Empathic Design as “often agile, flexible” [55], while Voinov et al. emphasizes Geographic Information Systems’ “ease of modification” [45].

This adaptability ensures researchers can respond to changing needs, improving research effectiveness. For example, Photovoice has been used with a wide range of populations, including children, adolescents, LGBTQAI + individuals, cancer survivors, single mothers, sex workers, adults with autism, and street-involved youth [33, 37, 54, 70, 72, 74, 78, 79, 82–84, 87, 90, 91, 95, 96, 99, 100, 102, 104, 145, 146].

Beyond flexibility, these methods prioritize simplicity, ensuring accessibility for participants with minimal training or technical expertise. Their user-friendly design allows for straightforward implementation. Parker et al. describe the Partnership Data Report for Reflection as “concrete, breaking up issues into manageable pieces” [127], and Visioning as “easy to use” [127]. By reducing complexity and providing immediate solutions, these methods ensure broad participation and meaningful engagement.

Co-creation methods in this theme offer practical tools for a variety of research and practice settings by balancing adaptability with simplicity.

#### Theme 7: impactful and valid

Impactful and Valid Outcomes are key benefits of 49 co-creation methods, offering actionable solutions, enhancing research rigor, and fostering learning opportunities. The three sub-themes (Impactful, Validity, and Skill Development) show how these methods lay a foundation for sustainable outcomes, support community-oriented solutions. Leurs notes that Checklists “worked very well for the policy and the firm frameworks” [110], while Participatory/reflective photography was praised for having “real-world applications that benefit individuals and organizations” [51].

By involving diverse stakeholders in decision-making, these methods enhance research validity, improve data accuracy, and capture community strengths. Lightfoot et al. highlight how Asset Mapping “can have a higher validity than data from traditional methods, as all stakeholders are involved in the selection of assets to be mapped” [151], while participants in Citizens’ Workshop “rated the efficacy of the process highly” [62]. These methods help ensure co-created interventions are well-informed, adaptable, and reflective of real-world conditions.

Beyond impact and validity, co-creation methods provide valuable skill development opportunities, equipping participants to engage meaningfully in research and decision-making. They foster mutual learning between researchers and end-users, creating opportunities for critical thinking, teamwork, and leadership development. O'Reilly-de Brún et al. describe how Commentary Charts “enhanced learning” [54], while Digital Storytelling “helps [participants] develop critical thinking skills” [68]. This capacity-building component strengthens participants' ability to contribute to and lead future co-creation efforts.

By integrating real-world applications, ensuring research validity, and building essential skills, co-creation methods contribute to both immediate and long-term improvements in research and practice.

#### Theme 8: reflection and understanding

Reflection and Understanding are key benefits of 57 co-creation methods, promoting self-directed learning, deeper insights, and clearer representation of complex issues. The three sub-themes (Reflection, Understanding Complexity, and Visualization) emphasize how these methods encourage critical thinking, help navigate multifaceted challenges, and provide visually engaging ways to communicate information.

By fostering iterative thought processes and personal transformation, co-creation methods create reflective environments where participants can engage in self-assessment and track progress. Revez et al. describe how the Modified Delphi promotes “reflexivity is a process of critical reflection involving researchers and participants interrogating their own paradigms” [152], while Photovoice has “the potential to facilitate self-reflection and increase the patient’s awareness of his/her successes and difficulties” [73]. These reflective practices help participants explore personal and social issues, deepening engagement and building greater awareness.

Beyond personal reflection, these methods help capture complex systems, stakeholder values, and service interactions, providing decision-makers with comprehensive insights into social and environmental issues. Voinov et al. illustrate how Agent-Based Models are “well suited for representing complex spatial interactions under heterogeneous conditions and for modeling decentralized, autonomous decision making” [45], while Litovuo et al. describe how a Narrative Interview “yielded comparatively deeper and broader data on the spatial complexity and multiparty nature of the service experience” [156]. By bridging cognitive limitations, co-creation methods ensure a more nuanced and immersive way to examine multifaceted topics.

Visualization plays a crucial role in this theme, offering clear and engaging ways to present complex data. These methods enhance the exploration and representation of health data, spatial patterns, and emerging trends, making information more accessible and actionable. Beyer et al. explain that Geographic Information Systems enhance “visual exploration and presentation of health data” and are useful for “creating large and visually appealing graphics” [117]. Visual elicitation raises issue visibility, stimulates discussion, and generates rich insights. For instance, “the use of images in the concept mapping process enabled participants to identify the natural connections between factors” [132].

By fostering critical reflection, improving understanding of complexity, and enhancing data visualization, co-creation methods empower participants to engage deeply, communicate effectively, and make informed decisions based on nuanced and visually compelling representations of information.

#### Theme 9: efficient and strategic

Efficiency and Strategic Thinking are key benefits of 15 co-creation methods, enabling streamlined processes, cost-effectiveness, and structured decision-making. The two sub-themes (Efficient and Strategic) demonstrate how these methods optimize resources, reduce costs, and enhance problem-solving by offering a broader perspective on complex issues.

These methods improve time efficiency and facilitate effective group discussions, allowing for rapid assessments and the integration of local insights with minimal staff assistance. They offer a structured yet flexible approach to addressing multiple challenges simultaneously, ensuring comprehensive yet manageable processes. Voinov et al. describe Fuzzy Cognitive Mapping as “a useful tool to quickly and efficiently evaluate the structure and function of a dynamic problem” [45]. This emphasis on efficiency makes these methods particularly valuable in resource-limited settings or fast-paced decision-making environments.

Beyond efficiency, these methods promote strategic thinking by maintaining an aggregate view of problem structures and encouraging stakeholders to focus on broader feedback loops rather than isolated details. Voinov et al. highlight Causal Loop Diagrams for their “ability to give an aggregate or strategic view of the problem structure which helps to keep [the] focus on feedback loops rather than on details” [45]. This strategic focus supports long-term planning, mitigation strategy development, and cross-cultural problem-solving, ensuring that decision-making remains holistic and forward-looking.

By optimizing resource use, encouraging strategic insights, and enhancing structured decision-making, co-creation methods in this theme support both efficiency and high-level problem-solving, making them valuable tools for addressing complex challenges across diverse contexts.

### Method challenges

**Fig. 5:**
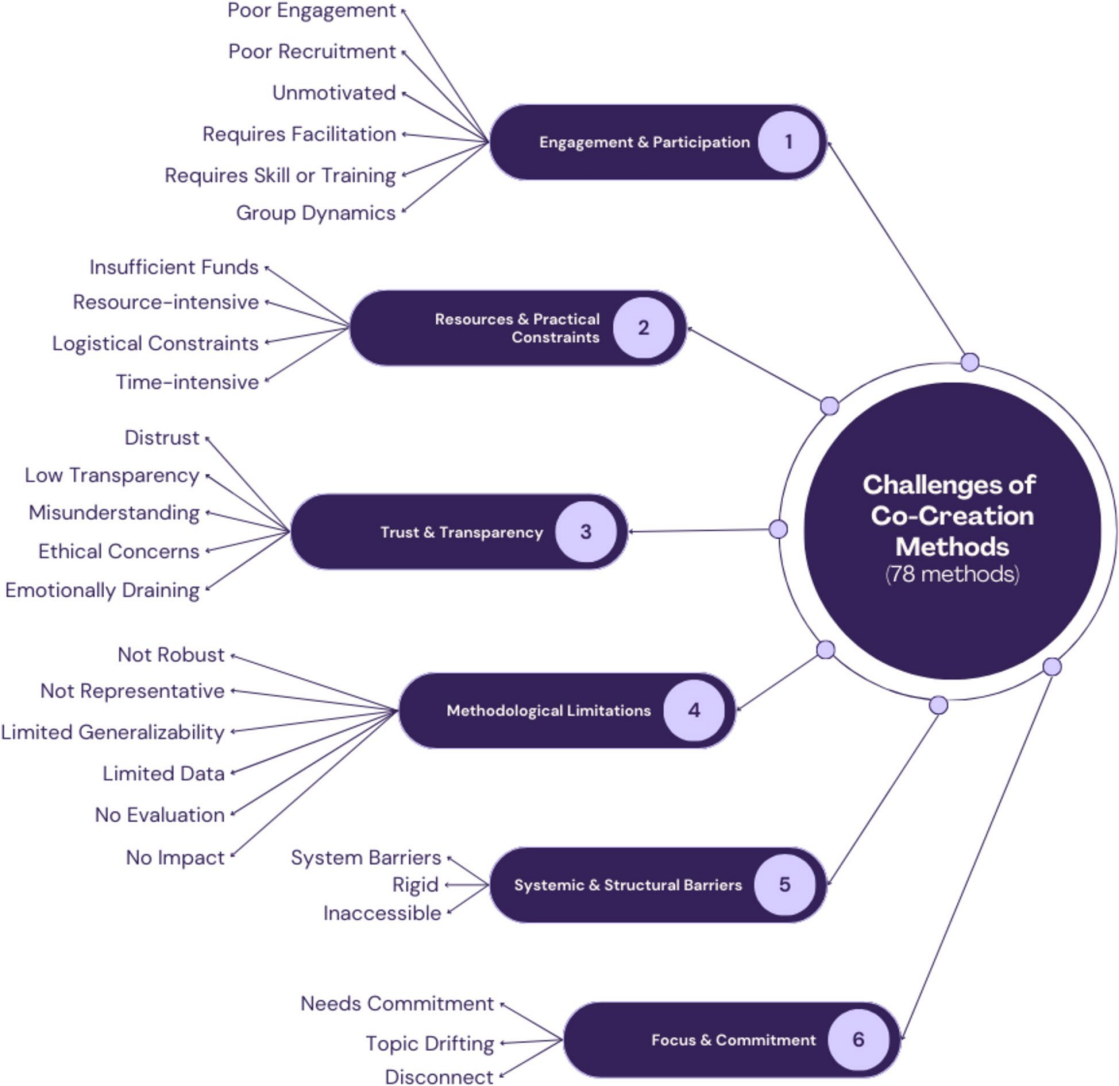
The reported challenges of 78 methods used in co-creation; including 6 themes and 27 sub-themes

**Fig. 6:**
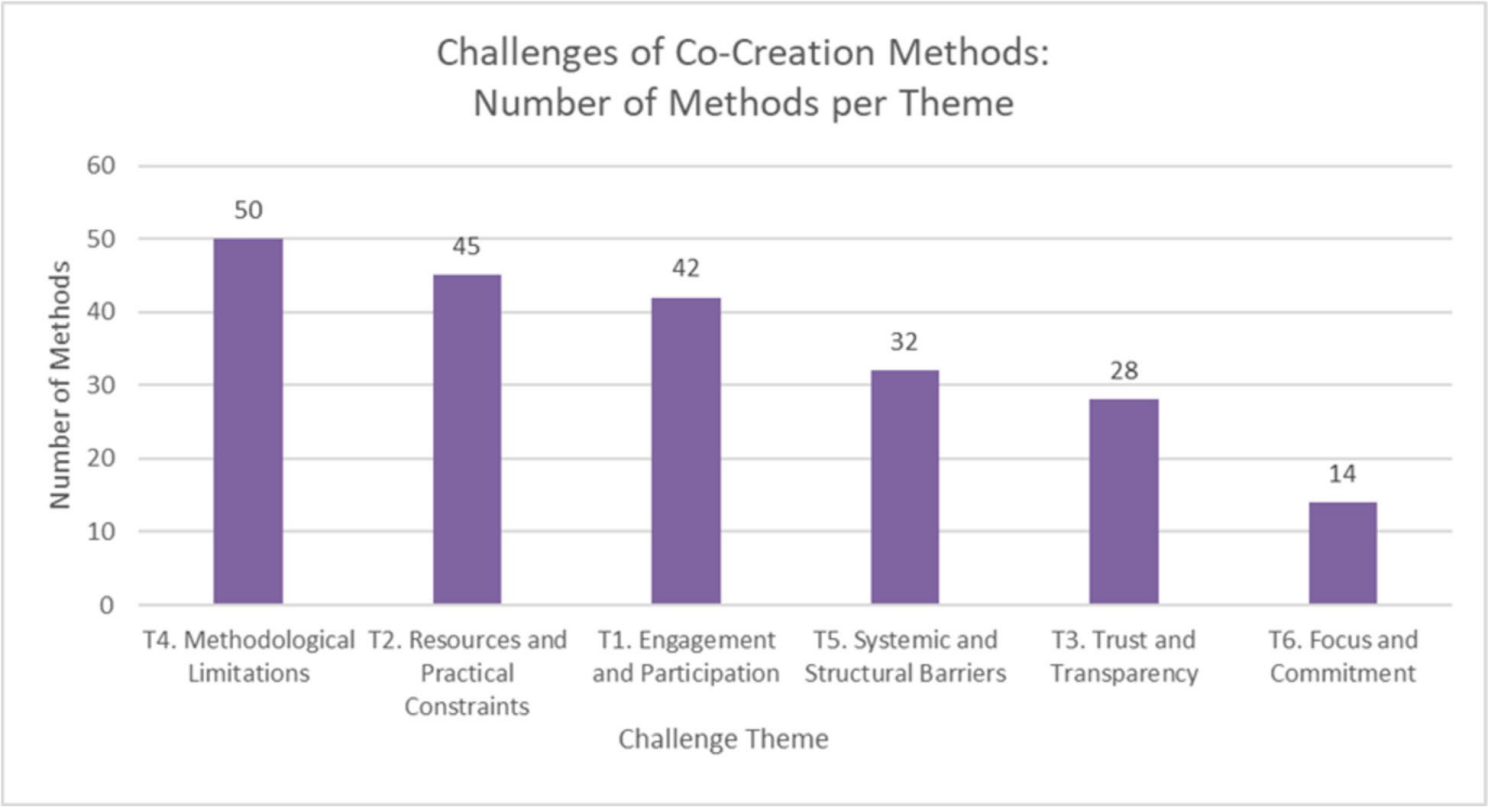
Graph of the number of methods that align with themes 1 through 6. The order of the sub-themes is from highest to lowest number of methods. T# equals the theme number

Data was extracted per method regarding the benefits and potential challenges when using the method: 90 articles reported the challenges of using 78 methods, and the following is a summary of the findings grouped into 6 themes and 27 sub-themes. These themes and sub-themes are visualized in Fig. 5, and an additional file contains the set of challenges per method (see Additional File 7), and the total number of methods per theme is visualized in Fig. 6.

An additional file contains the sub-theme names, descriptions, and associated methods (see Additional File 8).

#### Theme 1: engagement and participation

Challenges related to engagement and participation are common across 42 co-creation methods, affecting participant involvement, motivation, and the skills required for effective implementation. The six sub-themes (Poor Engagement, Poor Recruitment, Unmotivated, Requires Facilitation, Requires Skill or Training, and Group Dynamics) highlight barriers to participation, the need for specialized facilitation, and the complexities of managing group interactions.

Engagement challenges often arise when participants struggle to connect with research activities or feel constrained by the method’s format. Redman-MacLaren notes that participants in an Interpretive Focus Group “only analyzed a small amount of data when compared with the qualitative data available” [39], while Woolner et al. discuss how “adult participants…were reluctant to complete some of the activities” [60]. These issues are particularly evident in methods requiring creativity or unfamiliar forms of participation, where hesitation or discomfort can limit involvement.

Recruitment can also be difficult, particularly when relying on community gatekeepers or snowballing techniques. Furman et al. warn that “recruitment can represent a challenge” [31], while Van Loon et al. describe how participant selection was “dependent on the village leadership and our local research assistants for selecting and communicating with participants” [63]. Such approaches risk selection biases and make it harder to reach underrepresented groups.

Motivation presents another barrier. Methods that require significant time or effort can discourage participants, particularly when the perceived benefits are unclear. Lambert et al. illustrate that during Informal Interviews, “children may become bored with verbal interaction, reluctant to talk and give limited in-depth responses” [48], while Pereira et al. warn that the time-intensive process “can often limit the participation of some people unless they see a direct benefit for their work” [114]. Sustaining engagement requires well-structured activities that keep participants interested and invested.

Facilitation plays a critical role in many co-creation methods, with outcomes often hinging on the facilitator’s expertise. Without skilled guidance, participants may struggle to navigate discussions or derive meaningful insights. Leurs notes that participants relied on “the project team members about which issues they might wish to explore further” [110]. Samaddar et al. emphasize that “a lot of aspects of the workshop depend on facilitation skills of the facilitator” [162]. This reliance introduces variability and makes scaling these methods more challenging.

Training and skill requirements also pose significant challenges. Inadequate training can lead to errors in data interpretation and implementation. BeLeu et al. reports errors in Causal Loop Diagram exercise due to “inadequate training to conduct such an exercise” [66], while Lightfoot et al. highlight that “Asset mapping is time intensive and requires an extensive amount of training and oversight” [151]. Structured training programs are essential to ensure methods are applied consistently and accurately.

Finally, group dynamics can shape the success of co-creation processes. Dominant voices or social influences may skew narratives and suppress diverse perspectives. Wagemakers et al. stress that a Focus Group “needs strong facilitating skills to manage group dynamics” [163], while Kaptani and Yuval-Davis note that narratives are “affected by the other participants’ narratives” [34]. Effective management of group dynamics is crucial to creating an inclusive environment where all voices are heard.

By addressing challenges related to engagement, recruitment, motivation, facilitation, training, and group dynamics, co-creation methods can become more inclusive, effective, and sustainable across diverse research settings.

#### Theme 2: resources and practical constraints

Challenges in Resources and Practical Constraints affect the feasibility, sustainability, and engagement of 45 co-creation methods. The four sub-themes (Insufficient Funds, Resource-Intensive, Logistical Constraints, and Time-Intensive) highlight barriers related to financial limitations, high resource demands, logistical complexity, and significant time investment. These challenges are particularly evident in large-scale or long-term projects, where the resource-intensive nature of co-creation methods can become overwhelming [62, 63, 66, 112, 115, 158].

Insufficient Funds are a major barrier, limiting the ability to cover essential costs such as training, staff time, and participant incentives. Financial constraints often make it difficult to sustain projects. Van Loon et al. note that in Creative Practice, there was “not enough funding to evaluate the effectiveness” [63], while Furman et al. highlight how the lack of “funding posed interconnected challenges” [31]. Resource-intensive methods require substantial investments in materials, tools, and facilitation support. Fairchild and McFerran describe Collaborative Songwriting as “resource dependent” [112], while Forsyth et al. point out that Threshold Analysis “require a substantial resource commitment” [164]. Specialized equipment, digital tools, and trained facilitators are often essential, making careful resource planning a critical component of co-creation.

Logistical Constraints add complexity to co-creation, with careful planning needed for organizing activities, accessing confidential data, and managing fieldwork logistics. Green emphasizes the need for “logistical considerations when facilitating art activities with children especially in nature as art making can be messy” [47]. Similarly, scheduling interviews, producing educational materials, and handling large amounts of data require substantial effort, as noted in a study by Chavarria et al. [158]. These logistical hurdles can delay progress and limit the scalability of co-creation efforts.

Time-intensive methods require significant commitment from both participants and facilitators, which can hinder engagement, especially for those with competing responsibilities. Lightfoot et al. describe “Asset Mapping is time intensive” [151], while Zorrilla et al. note that “time-intensive training is required to master [Bayesian Networks]” [143]. These demands can reduce perceived value and make it difficult to sustain participation, particularly in fast-paced or resource-limited settings. Balancing meaningful participation with time constraints is essential to maintaining the value of co-creation approaches.

By addressing funding limitations, resource demands, logistical barriers, and time constraints, co-creation methods can be better adapted for practical implementation. Proactive management of resources, planning, and scheduling is key to ensuring their sustainability and long-term impact.

#### Theme 3: trust and transparency

Challenges in Trust and Transparency affect 28 co-creation methods, posing barriers to participant engagement, ethical considerations, and the clarity of research outcomes. The five sub-themes (Distrust, Low Transparency, Misunderstanding, Ethical Concerns, and Emotionally Draining) highlight issues related to participant hesitancy, interpretation complexities, and the emotional burden associated with certain methods.

Distrust and privacy concerns hinder participation, especially among participants from welfare or therapy contexts who may fear unintended consequences. Hesitation to share information due to concerns about confidentiality can result in selective participation and withheld responses. Valerio et al. note that “participants may not share information freely for fear of privacy or confidentiality” [148], making it difficult to capture their lived experiences. Trust and transparency are essential for encouraging meaningful participation, particularly for individuals from vulnerable backgrounds who may fear judgment or stigmatization [33, 46, 64].

Low transparency in co-creation methods raises concerns about the accuracy, effectiveness, and clarity of research outcomes. Some methods suffer from unclear goals or outputs, making it challenging for participants to understand their role or how findings are derived. As O’Reilly-deBrún describes Direct Ranking may also face challenges related to unclear outcomes or goals [54], while Voinov et al. showcase that Agent-Based Modeling (ABM) suffers from “low transparency,” [45]. These concerns may lead to skepticism and decreased engagement.

Misunderstandings in interpretation further complicate transparency and trust. Visual and artistic methods, while valuable for engagement, can be difficult to interpret accurately, risking misrepresentation. Lambert et al. explain that Draw and Write Techniques involve “interpretation challenges” [48], while North et al. describe how “the first sociograms produced by the [participants] were densely drawn, which made interpretation and analysis difficult” [121]. Clear communication and representation are essential to ensure accurate and comprehensive findings.

Ethical concerns related to consent, ownership, and data handling add another layer of complexity. Participant-generated content, particularly photographs, and visual data raise questions about who controls the data and how it is shared. Ronzi et al. highlight concerns about “photo ownership and individuals appearing in the photographs” and “challenges in photographing negative social concepts” [101]. Lambert et al. describe issues with “confidentiality challenges and ownership issues” [48]. Sensitive imagery and personal data raise ethical dilemmas regarding consent, confidentiality, and ownership [48, 49, 76, 95, 96, 100, 101, 145, 148]. Without robust consent processes, these concerns can lead to discomfort and reluctance to participate.

Beyond trust and transparency, some co-creation methods are emotionally draining, especially when they involve sensitive topics, personal reflection, or unfamiliar processes. Nomakhwezi Mayaba and Wood describe how the Draw and Write Technique “can induce negative feelings” [106], while Blodgetta et al. note the participants’ “experience of anxiety to draw something, [due to their] fear of negative judgment” [139]. Managing emotional responses and ensuring participant well-being is crucial for maintaining engagement and safeguarding participants’ mental health.

By addressing challenges related to trust, transparency, and ethical considerations, co-creation methods can become more supportive and inclusive. Ensuring clear communication, careful data management, and sensitivity to participants' emotional experiences fosters an environment where participants feel safe, valued, and empowered throughout the process.

#### Theme 4: methodological limitations

Methodological Limitations are prevalent across 50 co-creation methods, affecting research integrity, data quality, and broader applicability. The six sub-themes (Not Robust, Not Representative, Limited Generalizability, Limited Data, No Evaluation, and No Impact) highlight weaknesses in analytical frameworks, sample diversity, and real-world effectiveness. These limitations often necessitate the integration of complementary research methods to ensure a more comprehensive understanding of co-creation outcomes [129, 142, 167].

Concerns about methodological robustness arise from weak analytical rigor, a lack of validation processes, and potential biases introduced by convenience sampling. Some methods lack mechanisms to systematically establish traceability and verification, which affects their reliability. Dennerlein et al. comment that Co-design by Appropriation of Affordances faces several challenges, including a lack of “methods to systematically establish traceability and validation” [165]. These weaknesses can undermine research credibility and data accuracy.

Representativeness is another key issue, with many methods failing to capture diverse perspectives. Small sample sizes, self-selection, and the exclusion of certain groups reduce inclusivity and hinder the broader applicability of findings. Timotijevica and Raats observe that participants “did not think that the event captured a representative group of older people” [62], while O’Reilly-de Brún highlights how exclusionary criteria may offer limited representativeness if migrant participants are excluded [54]. These challenges can skew findings and limit their relevance for underrepresented communities.

Limited generalizability restricts the transferability of findings to new contexts or populations. Many co-creation methods are highly context-dependent, making it difficult to apply results beyond their original settings. As Scott-Bottoms and Roe note, Dialogic Art may limit the generalizability of the results because it “was a case study conducted in one specific region, with self-selecting participants” [160]. Without broader replication, these findings remain localized and difficult to scale.

Data limitations further weaken the depth and quality of insights. Some methods focus on linear relationships rather than capturing the complex interactions typical of co-creation. Voinov et al. illustrate how Fuzzy Cognitive Mapping “is largely limited to defining linear relationships between concepts” [45] while Lambert et al. point out that Informal Interviews “give limited in-depth responses” [48]. These constraints require the use of additional methods to obtain a more comprehensive understanding.

A lack of evaluation mechanisms also reduces the effectiveness of co-creation methods. Some approaches are rarely tested beyond isolated settings, limiting the evidence base for their impact. Kohfeldt and Langhout describe how The Five Whys Method is restricted to only one set because the “data [was] based on one [youth participatory action research] project within a school setting” [126], while Szczepańska et al. note that in Civic Budgeting “evaluation of the submitted proposals may be problematic because the ranking methods do not employ quantitative assessment criteria” [107].

Real-world impact is another challenge, with many methods struggling to influence policy or institutional change. Even when community input is gathered, a lack of follow-through or stakeholder engagement reduces the effectiveness of the process. Evans et al. observe that “pathways methods generated no uptake on behalf of the local government” [46].

By addressing these concerns related to rigor, representation, generalizability, data limitations, evaluation, and real-world impact, co-creation methods can be refined to enhance their credibility and broader applicability. Strengthening these areas will ensure that co-creation research produces robust, actionable insights with tangible outcomes.

#### Theme 5: systemic and structural barriers

Systemic and structural barriers in 32 co-creation methods create challenges for bottom-up planning, accessibility, and adaptability. The three sub-themes (System Barriers, Rigid, and Inaccessible) highlight how top-down decision-making structures, complex technical systems, and exclusive practices limit community engagement and reduce the diversity of insights. These constraints hinder co-creation’s potential to support inclusive, community-led solutions [45–47, 63, 64, 153, 164, 168].

System Barriers often restrict decision-making power at the community level by imposing rigid protocols and fragmented sectoral processes that complicate collaboration. Complex formal systems and incomplete data availability can further bias outcomes and deter participation. As Felker-Kantor notes, “participants whose daily activity paths cross geographic spaces with no data, exposure estimates will be biased” [118], while North et al. describe how “complex coding systems can be difficult to apply in real-time recording” [121]. Such barriers make implementing co-creation methods particularly challenging in decentralized or rapidly evolving environments.

Rigidity in certain methods limits their adaptability to dynamic systems and uncertain conditions, reducing their relevance in fast-changing contexts. Zorrilla et al. explain that Bayesian Networks are “not particularly well suited to dealing with dynamic systems because the computational burden required to solve probabilistic relations increases exponentially with the number of variables” [143], while Voinov et al. describe that Geographic Information Systems “cannot handle uncertainty” [45]. Methods that cannot accommodate emergent decision-making processes or unpredictable factors risk becoming outdated or ineffective in real-world applications.

Inaccessibility remains a critical barrier, particularly for marginalized groups and individuals with specific needs. Some methods rely heavily on technical knowledge, dense text, or public-facing formats that unintentionally exclude participants. Evans et al. warn that the “public nature of the activities can exclude marginalized groups” [46], and Felker-Kantor describes how “some participants were not accustomed to reading a map or giving directions” [118]. Additionally, Switzer et al. discuss how the Blended Approach: Photovoice and Photo-Elicitation, may be too advanced for some individuals with complex health needs, posing challenges to effective engagement [64]. Ensuring that methods are inclusive and accessible is crucial for capturing a broad range of experiences and fostering meaningful participation.

By addressing barriers related to system complexity, rigidity, and accessibility, co-creation methods can become more adaptable, inclusive, and effective, ultimately enabling more equitable and representative decision-making processes.

#### Theme 6: focus and commitment

Challenges in Focus and Commitment affect the successful implementation of 14 co-creation methods, particularly in maintaining participant engagement, ensuring alignment with research goals, and linking efforts to broader systems and policies. The three sub-themes (Needs Commitment, Topic Drifting, and Disconnect) highlight the importance of sustained dedication, staying focused on core objectives, and bridging co-creation with real-world impact.

Sustained Commitment is essential for co-creation but difficult to maintain over time, posing logistical and motivational challenges for participants, researchers, and institutions. Haque and Rosas note that Concept Mapping “required substantial commitment and investment on behalf of the participants, researchers, and supporting institution” [132]. Without consistent engagement, projects risk losing momentum and failing to produce meaningful outcomes.

Topic Drifting can arise in culturally diverse settings where differing perspectives shift discussions away from the original focus. Balancing authentic representation with maintaining structured discussions is a delicate task. Zorrilla et al. warn that “cultural peculiarities constrained the ability of [Bayesian Networks] to drive the process. This is because participants often drifted off to side issues” [143]. Effective facilitation can help manage this challenge by keeping discussions relevant while allowing space for organic dialogue.

A disconnect between co-creation efforts and broader systems or policies can hinder the long-term impact of these methods. Measuring the effects of interventions and linking them to specific policy changes is often difficult, limiting institutional support and uptake. Golden highlights that “very few photovoice studies report having actually influenced policy or policy makers, with many authors noting their inability to make contact with policy makers at all” [87]. Similarly, Van Loon et al. describe how their “interaction with policymakers was limited to a few exchanges at the start and end of the project” and they were “not as embedded in the community as [they] would have liked” [63]. Strengthening connections between co-creation outcomes and policy processes is crucial for translating research into meaningful action.

By addressing challenges related to commitment, focus, and systemic integration, co-creation methods can become more effective in engaging participants, maintaining project relevance, and influencing broader change.

## Discussion

This review shows that the literature on co-creation methods is both extensive and diverse, providing valuable insights into their application across various contexts and target populations. It offers evidence to support understanding and implementing these methods in public health settings, such as detailed descriptions of how methods have been applied in various contexts and with different target groups. While some of the findings may have broader applicability, their relevance to public health depends on the specific context. This information helps researchers and practitioners replicate and adapt these methods to their specific needs. This is particularly critical in public health, where interventions, services, and policies must often be tailored to diverse populations with unique cultural, social, and economic backgrounds, encouraging inclusion and equity.

The findings of this review can help researchers harness the full potential of co-creation to advance innovative and inclusive solutions in public health research. The literature on co-creation methods illustrates the challenges that may arise during the process, such as distrust, communication issues, and logistical constraints. By identifying these potential obstacles, this review provides valuable insights into how they can be overcome, thereby enhancing the likelihood of successful implementation. This review also provides a set of key benefits, showcasing how co-creation methods can increase public engagement, well-being, and satisfaction of the participants, and help develop solutions that meet community needs. Even though challenges such as limited generalizability of outcomes and poor evaluation can hinder their effectiveness compared to conventional approaches, co-creation fosters a sense of ownership and empowerment among participants, leading to higher levels of participation and adherence.

### Diverse target populations

Our analysis indicates that nearly half of the articles specified a target population, and engaging a diverse range of target populations shows the broad applicability of co-creation methods across various demographics. The varied target populations indicate that co-creation methods are being used to address the unique needs and perspectives of different groups, including marginalized and vulnerable communities. This reinforces the potential for co-creation to not only enhance research relevance but also to engage traditionally underrepresented voices in the public health discourse. The included articles most frequently engaged children and youth (40 articles), illustrating a concerted effort to involve younger populations in co-creation processes. However, despite this diversity, it is essential to recognize that certain populations, such as those with low literacy or mental health challenges, may still encounter barriers to effective engagement [46, 108, 146, 159].

### Participatory and co-creation research

Notably, the majority of methods identified were sourced from participatory research, participatory action research, and community-based participatory research. This finding reinforces the conclusions of Agnello and Loisel et al. that researchers risk overlooking essential literature relevant to co-creation if they focus exclusively on co-approaches (co-creation, co-production, and co-design) [8]. Additionally, the findings in this review align with previous research indicating that publications on methods in the co-approaches have primarily emerged since 2008, whereas participatory research methodologies have been contributing to this knowledge base since 1998 (as highlighted by Bauman [171] and Agnello and Loisel [8]). This perspective brings attention to the need for a more comprehensive examination of established participatory approaches to enhance co-creation practices.

### Method benefits

The diverse range of benefits associated with co-creation methods underscores their adaptability and effectiveness in addressing various research contexts and community needs. Co-creation enhances stakeholder engagement, fosters collaboration, and improves the relevance of research outputs [17]. In this review, the nine key benefits of co-creation methods align with the foundational goal of co-creation in public health research, to collaboratively address complex challenges in ways that traditional approaches cannot [14]. Categorizing these benefits into nine distinct themes revealed critical insights into how method selection can enhance research quality while promoting inclusivity and equity in public health outcomes.

A comparison with a recent review by Longworth et al., which investigated facilitators of co-creation in low- and middle-income countries, highlights several areas of alignment with this review's finding [172]. Longworth et al. emphasize the importance of creating safe spaces, building trust, fostering a sense of ownership, and selecting methods that fit the target population [172], which parallels the benefits identified here, particularly in the themes ‘Empowerment and Agency’ and ‘Well-being and Satisfaction’. However, this review also identified additional benefits not addressed in Longworth et al.’s work, offering a broader perspective on how co-creation can support public health research.

Several benefits identified in this review align with the work of Smith et al. [20] and Agnello et al. [9], who emphasized the role of co-creation and co-production in fostering innovation and creativity. In this review, ‘Innovation and Creativity’ emerged as the most prominent theme (91 methods), underscoring co-creation’s ability to stimulate novel solutions and encourage creative thinking in the creation of health interventions. These findings address gaps highlighted by An et al. [5] Agnello et al. [9], who pointed out the lack of reporting on creative methods. This review provides a wealth of information about methods that can enable creativity in co-creation.

Similarly, ‘Empowerment and Agency’ (65 methods) emphasizes the importance of empowering participants as active decision-makers, a point also advocated by Steiner and Farmer, who stressed the importance of giving participants in co-production the opportunity to influence decisions that affect their lives [173]. This review demonstrates the significance of empowering participants as co-creators with the agency to make decisions within the co-creation process. Empowerment is essential for ensuring that public health interventions are contextually relevant and culturally sensitive, as it shifts power dynamics away from traditional research practices toward more inclusive and participatory approaches [173, 174].

Other prominent themes include ‘Reflection and Understanding’ (57 methods) and ‘Impactful and Valid’ (49 methods), which contribute to the foundation for sustainable outcomes and community-oriented solutions. Co-creation methods hold significant promise for practical applications in public health by minimizing researcher bias and validating community knowledge, ensuring that the findings resonate with the lived experiences of participants [109, 157]. The integration of personal and social reflection not only clarifies stakeholder values but also strengthens intervention relevance.

In contrast ‘Efficient and Strategic’ (15 methods) were the least frequently reported. This points out a potential gap in the literature and an opportunity for further exploration of how co-creation can enable rapid assessments, integrate local insights efficiently, and foster strategic action planning.

The findings of this review demonstrate that co-creation enables diverse stakeholders to collaborate in ways that enhance collective learning and problem-solving, which are crucial for addressing complex health challenges. Future research should explore how these benefits can inform method selection to help researchers fully unlock the potential of co-creation in promoting inclusive and impactful research.

### Method challenges

While co-creation methods offer numerous benefits, this review highlights challenges when implementing them, emphasizing the complexities that hinder their effectiveness and widespread adoption. These challenges were categorized into six themes, revealing key challenges that need to be addressed to fully unlock the potential of co-creation.

A comparison with a recent review by Longworth et al., which investigated barriers to co-creation in low- and middle-income countries, sheds light on several areas of alignment with this review's finding [172]. Longworth et al. identified key barriers, namely, lack of financial investment, funding-constrains, systemic conditions, literacy level, recruitment strategy’s influence on process, building trust takes time, and lack of data and monitoring systems, which parallels the challenges identified here, particularly in the themes ‘Resources and Practical Constraints,’ ‘Systemic and Structural Barriers’, ‘Engagement and Participation,’ ‘Trust and Transparency’ and ‘Methodological Limitations.’ However, this review also identified additional challenges not addressed in Longworth et al.’s work, offering a broader perspective on potential challenges faced when conducting co-creation.

The most frequently reported challenges were ‘Methodological Limitations’ (50 methods) and Resources and Practical Constraints’ (45 methods), each pointing to critical areas for improvement. The theme ‘Methodological Limitations’ reflects the need for greater standardization and robust evaluation of co-creation methods to enhance their reliability and rigor. This aligns with the recent work of Grindell et al. who called for robust evaluation to ascertain the extent to which co-creation, co-design, or co-production improves health outcomes [3]. Furthermore, common issues within this theme include a lack of robust analytic processes, limited generalizability, and insufficient data, which weaken the overall impact of co-creation initiatives. These findings align with previous critiques of participatory research methods by Cargo and Mercer, which emphasize the importance of balancing methodological rigor with inclusivity [175].

Similarly, ‘Resources and Practical Constraints’ emphasize the resource-intensive nature of co-creation, which often requires significant funding, time, human resources, and support. Barriers such as insufficient funds, and time-intensive and resource-intensive processes highlight a broader systemic issue in our current research and funding systems, which are not designed to support the inherently flexible and creative nature of co-creation. Donor-driven timelines and budgets often fail to account for the time and resource demands of co-creation. Rigid institutional frameworks further restrict the adaptability needed for dynamic and iterative co-creation processes. Furthermore, rigid institutional frameworks and research processes leave little room for the adaptability needed to accommodate the dynamic and iterative nature of co-creation, as shown by the challenge of ‘Systemic and Structural Barriers’ (32 methods).

The theme ‘Engagement and Participation’ (42 methods) reveals the difficulties in motivating and sustaining participant engagement, particularly when verbal-only methods such as interviews are used. Complex methods often require training and specialized skills for implementation, making engagement challenging, and creating a reliance on skilled facilitation and guidance. One of the primary obstacles is the engagement and participation of stakeholders, which can be affected by factors such as reluctance to participate [48, 60]. These findings align with a broader discussion by Cargo and Mercer on the importance of capacity-building in participatory research to support long-term engagement and sustainability [175].

In contrast, ‘Focus and Commitment’ (14 methods) represents the least frequently reported theme but still highlights critical challenges. Methods that require substantial dedication from all stakeholders carry a higher risk of failure, especially when timeframes are extended or when cultural differences divert attention from the main process. Misaligned interventions can result when broad geographic scopes complicate data collection and analysis, limiting the overall impact.

Despite these challenges, co-creation methods still hold significant promise for enriching public health research. Addressing these barriers requires strategic planning, effective resource allocation, skilled facilitation, and clear communication to ensure meaningful and inclusive engagement and successful outcomes. More importantly, structural and system-level changes are necessary to create environments where flexibility, creativity, and inclusivity can thrive. Future research should explore how to overcome these barriers by promoting adaptive frameworks, improving capacity-building efforts, and advocating for policies that support participatory research.

### Applying co-creation methods

Notably, 49.5% of the articles reported intangible outputs, which emphasizes that co-creation methods yield not only tangible and expected results but can also foster collaboration and relationship-building among stakeholders. This finding reinforces the goal of co-creation to empower diverse voices. Additionally, the necessity for facilitators (indicated by 68.2% of methods) brings light to the complexity of implementing effective co-creation. Skilled facilitators play a critical role in guiding discussions and ensuring equitable participation, particularly among marginalized and vulnerable populations. This calls for enhanced training and resources to equip researchers with the tools needed to navigate diverse group dynamics successfully; or additional funding for researchers to hire services from trained process facilitators. Moreover, the finding that 51.5% of articles provided examples of methods used in conjunction with other methods underscores the complex nature of co-creation. This emphasizes the need for methodological diversity and flexibility in designing co-creation processes.

### Consequences and future research

This review revealed that while there is indeed literature about co-creation methods, however, there is no consensus about each method. Many methods have different names but are likely the same thing, such as Photovoice, Photoelicitation, and Participatory Photography. Furthermore, even when the same method name is used, the method is classified as a different type in different sources, for example, Concept Mapping is reported as a Qualitative, Participatory, and Mixed Method. This disconnect and lack of standardization of these methods call for the development of a taxonomy to organize methods to provide a structure to categorize and define the diverse array of co-creation methods. This will facilitate a more coherent understanding, application, and reporting of these methods across various contexts. Such a taxonomy can standardize the terminology, reduce ambiguity, and enhance communication among researchers, practitioners, and all relevant stakeholders. Moreover, organizing co-creation methods into a taxonomy supports researchers in selecting the most appropriate methods based on specific research questions or intervention goals. This is crucial in public health and other fields, where interventions must be contextually relevant and culturally sensitive. Finally, this taxonomy can support the evaluation and comparison of co-creation methods, for instance by tagging the methods based on the known benefits and challenges. This comparative analysis can lead to the identification of best practices and the refinement of existing methods, ultimately advancing the field of co-creation.

The detailed breakdown of methods utilized by each target population serves as a valuable resource for researchers looking to replicate successful strategies in their work. In addressing the challenges encountered with co-creation methods, future studies should focus on developing tailored approaches that specifically address these obstacles, ensuring that all voices are heard and valued throughout the co-creation process. Additionally, improving the capacity of researchers to apply the methods sourced in this review, as well as exploring how these methods can be combined, is crucial for enhancing the effectiveness of co-creation efforts and capturing diverse stakeholder perspectives. As described by Voinov et al., careful and conscious selection of methods, and combinations of methods, is important for the modeling processes and their outcomes. Ideally, the selection would be accompanied by evaluations to monitor the impact of individual methods on the process [45]. This emphasizes the need for informed method selection, as well as guidance on how to evaluate methods to enable assessment of effectiveness and impact.

Additionally, the findings in this review indicate a clear preference for face-to-face delivery modes, highlighting a potential gap in the literature regarding online or hybrid co-creation methods, which have become increasingly relevant in today’s digital landscape. Further exploration of how different delivery modes impact the effectiveness and accessibility of co-creation methods could provide important insights for future studies. The integration of digital tools, as highlighted in 40 articles, points to a growing trend in leveraging technology to enhance co-creation processes. The range of tools utilized, ranging from Geographic Information Systems to common software like Microsoft Excel,—suggests a shift toward more digital practices. This can impact the inclusiveness of co-creation processes, as some socially vulnerable or marginalized communities may not have access to digital tools and platforms. Future research should investigate how these digital tools may facilitate or hinder broader participation and engagement among diverse populations.

### Limitations

This review relied on a pre-screened and curated database containing literature from 1970 to 2022, which may have excluded some relevant articles published after 2022. However, the primary aim was not to capture the most recent literature but to analyze the existing body of work on co-creation methods, including those applied to marginalized and vulnerable communities. This focus seeks to promote improved referencing and reporting practices and learning from best practices. Additionally, the findings are limited by the information provided in the source articles, which may not include all relevant data or insights about co-creation methods. Furthermore, the search was limited to English due to the language limitations of the study team, which may have resulted in the exclusion of some articles published in other languages. However, the large number of methods identified suggests that additional literature in other languages would likely have made the study unmanageable. Finally, this review did not assess the quality of the included articles as this was not aligned with our research questions, which focused on characterizing and gathering all the relevant scientific literature on co-creation methods. Additionally, to our knowledge, there is currently no established or standardized approach for evaluating the quality of co-creation studies or methods.

## Conclusion

Literature on co-creation methods is indispensable for informing the design, implementation, and evaluation of co-creation for public health. It supports the development of innovative and collaborative co-creation processes that are responsive to the needs of diverse populations, thereby enhancing the overall effectiveness and cultural sensitivity of the outcomes. Consequently, this review is a valuable resource for future co-creation research. It represents an essential step in addressing the well-documented challenge of poor reporting of methods used in co-creation [3, 9, 20], ultimately advancing the field of co-creation and enhancing its evidence base and trustworthiness.

Our findings showcase the multifaceted nature of co-creation methods and their implications for promoting equity and inclusion in public health research and increasing the efficiency and applicability of co-created outcomes. By focusing on these critical areas, we can deepen our understanding of co-creation and its potential to reduce health disparities. Involving diverse target populations in co-creation processes is vital for developing equitable health interventions that respond to the needs of all community members. Co-creation can be inclusive of marginalized populations by implementing strategies and best practices supported by scientific literature. This review provides that literature, as well as key reflections regarding the diverse participation, the importance of building trust and mutual respect, as well as being able to continually adapt to changing circumstances, resources, and dynamic contexts.

By illuminating the various methods employed with these populations, this review contributes to the ongoing development of inclusive research practices. The insights from this review highlight the diverse advantages of co-creation methods. By recognizing and leveraging both the benefits and challenges of co-creation methods, researchers can improve the quality, relevance, and inclusivity of their work, ultimately leading to more equitable health outcomes for diverse populations. These findings call on researchers and practitioners alike to embrace co-creation, and its various methods, as a fundamental approach in public health research and practice.

## Supplementary Information

Additional file 1.

Additional file 2.

Additional file 3.

Additional file 4.

Additional file 5.

Additional file 6.

Additional file 7.

Additional file 8.

## Data Availability

All data generated or analysed during this study are included in this published article and its supplementary information files.
