# Supplementary material for: Co-creation methods for public health research — characteristics, benefits, and challenges: a Health CASCADE scoping review
Source: BMC Med Res Methodol. 2025 Mar 6;25:60. doi: 10.1186/s12874-025-02514-4 (PMC11884017; doi:10.1186/s12874-025-02514-4)
Supplement: Supplementary file 1 — Additional file 1. [file 12874_2025_2514_MOESM1_ESM.pdf]

Multimedia Appendix 1. Extraction Form

The Extraction form will include the following fields:

The researcher will be asked to complete a Google Form per article, containing the following question and answer options. It is possible that all the information will not be available, so there are response fields such as "unclear" and "not mentioned" to allow the research to indicate when the information is not available.

Form Fields:

Researchers' name (first and last): ________________

Date of extraction (dd/mm/yyyy): ________________

Section 1. Details on the Article

This section is for gathering basic bibliographic data on the article you're extracting from

- Article DOI:
- Article Title:
- Publication Year:
- Authors of the article (all):

Section 2. Details on the method(s)

This section is the main part of the extraction table, for gathering descriptive information about the method described in the paper. Please note: this section has some free response questions, so if the information is not described, please write "not described" as your response.

- What is the name of the method?
- What are the steps of the method?
- What is the purpose of the method (e.g., the aim of what you're trying to achieve)?
- What kind of method is it? (Select all that apply)
    - Quantitative
    - Qualitative
    - Mixed (quantitative and qualitative)
    - Participatory
    - Not described
    - Unclear

- What data can be used as an input of the method (e.g., free response, list of challenges, etc.)?
- What is the expected output of the method (e.g., the data that will come out of the method, grouped challenges, photographs, etc.)?
- Are there any intangible outputs for the method described (e.g., building trust, relationship building, etc.)?
    - Yes
    - No
    - Unclear
- If yes, please describe the expected intangible outputs:
- Does it mention how long the methods would take to execute (e.g., minutes, or days)?
    - Yes
    - No
    - Unclear
- If yes, how long + what unites (e.g., 60 minutes, 2 days)?
- What is the described mode of delivery for the method (select all that apply)?
    - Online
    - Face-to-face
    - Hybrid
    - Not described
- What kind of material resources do you need to execute this method? (e.g., post-its, camera, projector, online brainstorming tool, etc.) Please list all mentioned resources:
- What kind of human resources do you need to implement the method (e.g., someone to capture notes, a translator, a facilitator, etc.)?
- Does it mention the pros of the method?
    - Yes
    - No
    - Unclear
- If yes, please describe all the pros of the method (+ page #):
- Does it mention the cons/challenges of the method?
    - Yes
    - No
    - Unclear
- If yes, please describe all the cons of the method (+ page #):
- How many participants can engage in this method (exact number or range)?
- Do you need a facilitator to execute this method?
    - Yes
    - No
    - Not described
- Does it describe a certain target population where this method is ideally used?
    - Yes
    - No
    - Unclear
- If yes, what target population(s) is this method intended for (+ page #):
- Do they give an example of when this method is used/applied?
    - Yes
    - No
    - Unclear
- If yes, please input a short description(s) of all the example(s) (+ page #) :

- Does it talk about other methods that can be used with this method?
    - Yes
    - No
    - Unclear
- If yes, please describe (+ page #):
- Any other comments about the method:

Section 3. Supportive Data

This section focuses on gathering data that is about the full co-creation process surrounding the method(s). However, it is possible this kind of information is not accessible in the articles, so if you cannot find the information, please still respond to the questions with the appropriate response.

- Is a process/model/framework used for co-creation described in the text?
    - Yes
    - No
    - Unclear
- If yes, what process/model/framework is mentioned?
- If yes, are the stages of the process/model/ framework mentioned?
    - Yes
    - No
    - Unclear
    - A process/model/ framework is not mentioned
- If yes, what were the stages of the co-creation process/model/framework? Please insert the names of the stages, in order + page number where you found them:
- Does the paper mention an associated digital tool or software (e.g., Mural, Lucid chart, etc.)?
    - Yes
    - No
    - Unclear
- If yes, what is the name of the digital tool(s) + the page number where you found it:
- Does the paper mention a supportive research methodology (e.g., community-based participatory research, holistic person-centered approach, an integrated experience-based co-design approach, co-production, etc.)?
    - Yes
    - No
    - Unclear
- If yes, what is the methodology:
- Any additional comments or information you think is relevant:
