# Supplementary material for: Co-creation methods for public health research — characteristics, benefits, and challenges: a Health CASCADE scoping review
Source: BMC Med Res Methodol. 2025 Mar 6;25:60. doi: 10.1186/s12874-025-02514-4 (PMC11884017; doi:10.1186/s12874-025-02514-4)
Supplement: Supplementary file 5 — Additional file 5. [file 12874_2025_2514_MOESM5_ESM.pdf]

# Additional File 5. Method Combinations

There were 174 instances where the authors reported methods that were used together in the co-creation process, this information provides insights into how methods can be combined. The extracted combinations were grouped by method type and then visualized in a Sankey Diagram in Figure 1, and the combination frequency was depicted in Table 1. Table 2 contains the detailed combinations of the methods.

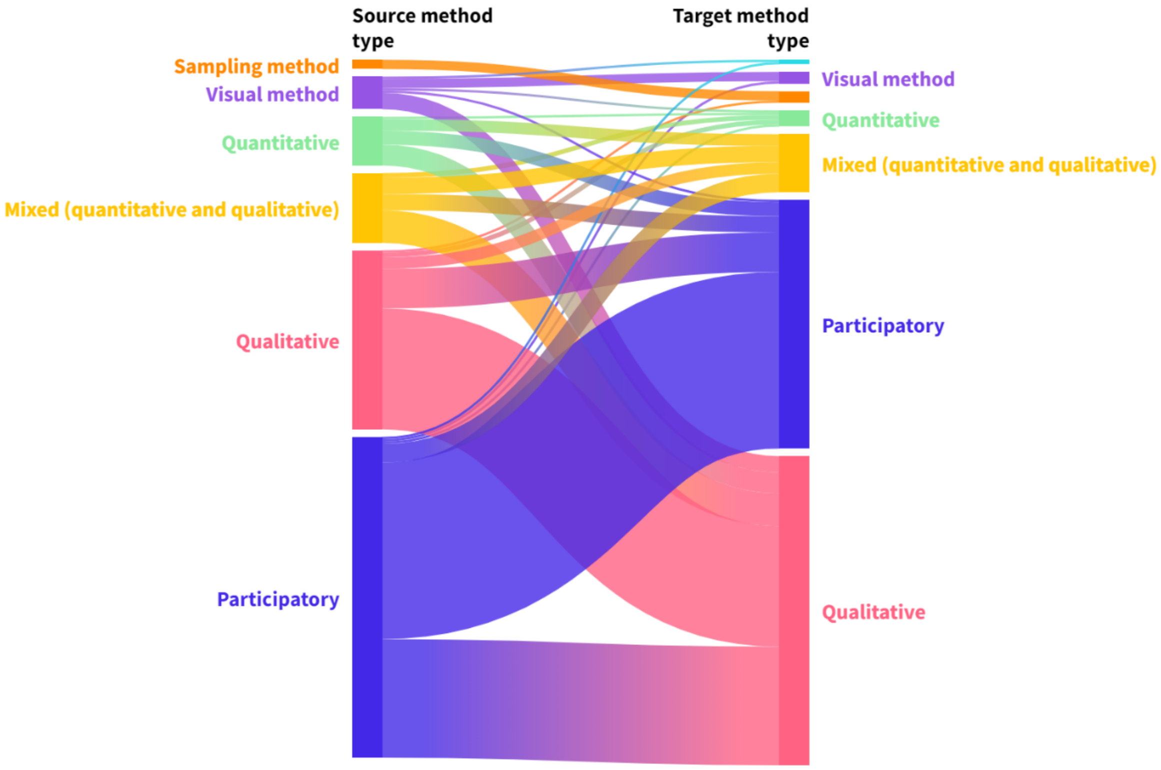


*Figure 1. Co-occurrence of methods by type. The source method was the method type for those methods that were extracted (left), and the target method type as those that were reported as used with the source method (right)*

*Table 1. Methods Combinations (by type) and their frequency of co-occurrence*

| Source Method Type | Target Method Type | Frequency % (n=258) |
|---|---|---|
| Participatory | Participatory | 26.7% (n=76) |
| Qualitative | Qualitative | 18.2% (n=52) |
| Participatory | Qualitative | 17.9% (n=51) |
| Qualitative | Participatory | 6% (n=17) |
| Mixed | Qualitative | 4.9% (n=14) |
| Quantitative | Qualitative | 3.2% (n=9) |
| Mixed | Participatory | 2.5% (n=7) |
| Mixed | Mixed | 2.5% (n=7) |
| Visual | Qualitative | 2.5% (n=7) |
| Quantitative | Participatory | 2.1% (n=6) |

| | | |
|---|---|---|
| Qualitative | Mixed | 1.8% (n=5) |
| Quantitative | Mixed | 1.8% (n=5) |
| Visual | Participatory | 1.4% (n=4) |
| Visual | Visual | 1.4% (n=4) |
| Sampling | Sampling | 1.4% (n=4) |
| Qualitative | Quantitative | 0.7% (n=2) |
| Mixed | Quantitative | 0.7% (n=2) |
| Qualitative | Sampling | 0.4% (n=1) |
| Quantitative | Quantitative | 0.4% (n=1) |
| Participatory | Visual | 0.4% (n=1) |
| Visual | Quantitative | 0.4% (n=1) |
| Quantitative | Visual | 0.4% (n=1) |
| Visual | Observational | 0.4% (n=1) |
| Participatory | Observational | 0.4% (n=1) |

*Table 2. Combination of methods from the included studies*

| **Method 1** | **Methods** |
|---|---|
| A household survey | Photovoice |
| Agent-based modeling | Participatory modeling |
| Alternative scenarios | Visioning; pathways; projections |
| Art-based narrative interview | Present and future learning; interactive-relational approach |
| Asset mapping | Interview; focus group; survey |
| Audio-recorded semi-structured interviews | Photo walkabouts; focus group |
| Bayesian Networks | Group model building; participatory modelling |
| Body mapping | Individual interviews; reflective notes |
| Body measurements | Concept mapping; questionnaires; focus group; logic model; capacity mapping |
| Bookmaking | Role-playing; building a model; art making |
| Capacity mapping | Concept mapping; logic model |
| Carer's Assembly | Policy dialogue |
| Causal loop diagram | Graphs over time; cognitive mapping; System dynamics modelling |
| Checklists | Venn diagrams; master charting |
| Citizens' Jury | Citizen's workshop |
| CLUE | World Café; Research Café |
| Co-design by Appropriation of Affordances | Think aloud |
| Cognitive mapping | Causal loop diagrams; graphs over time; concept mapping; mind mapping |
| Commentary Charts | The Direct Ranking |
| Community mapping | A digital basemap; focus group; individual mapping interviews; dot map focus group; The Geographic Information Systems (GIS) |
| Concept mapping | Logic model; capacity mapping; Multidimensional scaling; Photovoice |
| Consensus conference | Focus group; questionnaire |

| | |
|---|---|
| Creative practice | Photography; music and song; storytelling; video and film; dance and poetry |
| Daily activity space travel diary | Participatory geographic mapping |
| Daily work schedules | Mapping; Venn diagrams; seasonal calendars |
| Decision tree analyses | Semi-quantitative modelling; quantitative modeling |
| Delphi Method | Cognitive interview |
| Dialogic art also referred to as relational art | Practice as Research; storytelling |
| Diamond ranking | Photo-elicitation |
| Direct Ranking | The Commentary Charts |
| Dot map focus groups | Dot map focus group |
| Draw and write technique | Collage making |
| Drawing | Focus group |
| Empathic Design | Design Probing, photography, storytelling, personal interviewing |
| Engaged scholarship | Spherecards; Interview guide; Theatrical monologues, "An exhibition with "pieces of art"; Cartoons; Posters; Booklets and fairytales; Postcards |
| Ethnographic Facilitation | Interview; questionnaire |
| Ethnographic study | Semi-structured interview; personas; content analysis |
| Experience mapping | Prototyping; wild card; persona building |
| Expert interviews | Thematic analysis |
| Five Field Map | Photovoice |
| Focus group | Interviews; concept mapping; logic model; capacity mapping; Collage making |
| Forum Theatre | Individual interviews; The post-performance interviews; Forum Theatre; semi-structured interview |
| Fubimethod | Wizard of Oz prototyping, Dwelling space technique; kidreporter method; situated bodystorming techinque; Puppet-based design technique |
| Future workshop | Management workshop; Future wheel; mind mapping |
| Geocaching games | Focus group; photo scavenger hunt game; geocaching games |
| Geographic Information Systems | Coupling system dynamics model; Community mapping; Participatory mapping |
| Go along interviews | Photo journals; discussion groups; participant observation; field notes; photo logs; questionnaire; focus group; Photovoice; GPS tracking; semi-structured interview |
| GPS Tracking and Interviews | Photo journals; discussion groups; participant observation; field notes; photo logs; questionnaire; focus group; go-along interview; Photovoice; semi-structured interview |
| Graphic Facilitation | Focus group; interview |
| Graphs over time | Cognitive mapping; causal loop diagrams |
| In-depth interviews | Body measurements; concept mapping; focus group; questionnaire; capacity mapping; logic model |
| Informal interviews | About myself; semi-participant observation; draw and write technique; stick-a-star quiz |
| Interpretive focus groups | Storyboarding |
| Interview | Body mapping; reflective notes |
| Kitchen table talks | Photo walkabouts; interview; focus group |
| Learner verification and revision | Semi-structured interview; probing questions |

| | |
|---|---|
| Legoreal play | Content analysis |
| Living Lab | Storyboarding; animations |
| Illustrative arts-based method | Interview |
| Logic model | Concept mapping; capacity mapping |
| Mandala drawing | Conversational interview |
| Mānoa mash-up method: facilitating participatory scenario building | The Anthropod |
| Mapping | Daily work schedules; seasonal calendars; Venn diagram |
| Metaplan method | Problem Tree |
| Mind mapping | Focus group; individual interview |
| Mockups of webpages | Observation; in-depth one-on-one consultation; thematic analysis |
| Modified Delphi | Semi-structured interview; focus group; citizen juries |
| Multiple Criteria Analysis | Focus group; interview |
| MUST method | Prototyping; scenarios; hearings; observations; in-situ interviews; workshop; think aloud |
| Participant observation | Community Dialogue Group; Community Meeting; workshop; semi-structured interview |
| Participant Photography | Interview; focus group; Photographs during focus group; thematic grouping; field diary |
| Participatory geographic mapping | Baseline questionnaire; daily activity space travel diary; daily behavior diary; paper-based travel diary |
| Participatory mapping | Geographic Information System |
| Participatory Theme Elicitation | Focus group |
| Participatory Video | Focus group; interview |
| Participatory visual methodology | Focus group |
| Participatory/reflective photography | Thematic analysis; Coding book |
| Partnership Data Report for Reflection | Workshop; Promising Practices Guide for Reflection |
| Pathways | Visioning |
| Persona Building | Wild cards; prototyping; experience mapping |
| Persona technique / Personas | Personas; collaborative mapping; observational techniques |
| Personas | Ethnographic study |
| Photo-elicitation | Diamond ranking; picture sorting; the giant photograph; focus group; in-depth interview; graphic facilitation; sociogram; interview |
| Photo production with interviews | Interview |
| Photo walkabout | Interview; focus group; kitchen table talks |
| Photovoice | Forums; semi-structured interview; The showed method; photo journals; discussion groups; participant observation; field notes; photo logs; questionnaire; focus group; go-along interview; mapping of walked route; GPS tracking; critical dialogue; interview; Group discussions; multi-pronged evaluation; Inductive analysis; deductive analysis; phenomenology; survey questionnaire; thematic analysis; systematic neighbourhood observation; household survey; one-on-one interviews |
| Plan Review Checklists | A short audit tool; Rapid HIA; The Threshold Analysis |

| | |
|---|---|
| Playback Theatre | Forum Theatre workshop; post-theatre individual interview |
| Policy café | Carer's Assembly; Filming |
| Powerview | Semi-structured interview; Participant observation; field notes |
| Prototyping | Persona building; experience mapping; wild cards |
| Purposive sampling | Purposive sampling; convenience sampling |
| Q methodology | Interview |
| Q sorting-paper-based sorting approach | Interview; focus group |
| Questionnaire | Focus group; Body measurements; in-depth interview; concept mapping; logic models; capacity mapping |
| Rapid HIA | A short audit tool; The Threshold Analysis; Plan Review Checklists |
| Reflective Drawing | Interview |
| Reflective notes | Interview, body mapping |
| Research Cafe | CLUE |
| Role-playing | Bookmaking; MAS model; Agent-based modeling; ComMod approach |
| Seasonal calendars | Venn diagrams; mapping; daily work schedules |
| Self-documentation | Content analysis |
| Semi-structured interview | Participant observation; live testing; interactive testing; qualitative data analysis |
| Semi-participant observations | Informal interview |
| Sensemaking | User stories |
| Short audit tool: the Preliminary Checklist | Rapid HIA; Threshold Analysis; Plan Review Checklists |
| Situation analysis tool | Telephone-based screening tool |
| Snowball sampling | Convenience sampling |
| Social Network Analysis | Multi-criteria analysis; Stakeholder Analysis; interview, focus group; participatory mapping process; |
| Sociogram (directed graph) | quantitative analysis; Photo-elicitation; graphic facilitation; focus group; interview |
| Stakeholder Analysis | Snowball sampling; social mapping |
| Stakeholder research interview | Rapid review of the grey literature; Model development workshop |
| Story board | Word search |
| Storyboard & animations | Living lab |
| Storytelling | Field notes; memos; journaling; LEGO Serious Play |
| Strategic Environmental Assessment | Problem structuring method; public participation geographic information system |
| Structured surveys | Interview |
| The Five Whys Method | Photovoice |
| The Three-step test-Interview | Literature review; review of secondary analysis datasets; focus group |
| Think aloud | Mock patient encounters; focus group; observations, storytelling |
| Threshold Analysis | A short audit tool; Rapid HIA; Plan Review Checklist |
| Transdisciplinarity | Bidirectional Emic–Etic Tool; interview |
| Transect walk | Geo-referencing; workshop; participatory GIS |
| User driven systematic review | Discussion groups; consensus method |

| | |
|---|---|
| User stories | Future scenarios; customer journey mapping; scenario writing; collective mind map; open spaces; World Café |
| Venn diagrams | Mapping; daily work schedules; seasonal calendars |
| Video diary | Qualitative data analysis |
| Visioning | Alternative scenarios; pathways; projections; River of Life |
| VR festlab | Interviewing; brainstorming |
| Wild Cards | Experience mapping; prototyping; persona building |
| Word Search | Storyboarding |
| Youth react (Research Actualizing Critical Thought) data analysis method | Photovoice; expert panel |
| Zaltman Metaphor Elicitation Technique | Construct elicitation; thematic analysis |
