# Supplementary material for: Co-creation methods for public health research — characteristics, benefits, and challenges: a Health CASCADE scoping review
Source: BMC Med Res Methodol. 2025 Mar 6;25:60. doi: 10.1186/s12874-025-02514-4 (PMC11884017; doi:10.1186/s12874-025-02514-4)
Supplement: Supplementary file 6 — Additional file 6. [file 12874_2025_2514_MOESM6_ESM.pdf]

Additional File 6. Methods Pros and Cons Codebook

Codes

Name	Description	Example text	Indicator
**BENEFITS OF METHODS (Q1)**			
**Theme 1) Collaboration and Participation**			
1. Collaboration	Enables people to work together.	“method embraces a participatory and collaborative approach” and “It fosters a collaborative approach to problem-solving.”	“collaborate”; “collaborative”; “collaboration”
2. Meaningful Participation	Enables participation in a meaningful, non-tokenistic way	“It enhances participation, as participants engage actively” and “making them active participants rather than mere subjects.”	“participate”; “participation”; “engagement”
3. Socializing and Connection	Enables connection among stakeholders, or increases social cohesion or socializing among stakeholders (including the researcher).	“Building a connection with the community” and “allowing participants to connect through shared artistic expression”	“connecting”; “connection”; “socializing”
**Theme 2) Empowerment and Agency**			
4. Decision-making and democracy	Enables some form of decision-making, individually or collectively, or enables democratic processes.	“decentralized, autonomous decision-making processes” and “transform decision-making by incorporating environmental values.”	“decision-making”; “democracy”; “discussion”
5. Heard and supported	Those involved feel that they are heard and they are supported in the process.	“while also enabling them to share their insights and experiences” and “sense of being heard.”	“support”; “understand”; “heard”
6. Sense of Ownership	Those involved feel a sense of ownership of the process, the data, the outcome, etc.	“fostering ownership” and “allows individuals to create their own maps rather than using pre-drawn maps or census boundaries.”	“ownership”; “possession”; “own”
7. Empowerment	Enables the empowerment of those involved through the transfer of	“empowers marginalized and stigmatized groups” and “positioning participants as	“empower”; “equal”; “power”

	power, an evening of power dynamics, or increases a sense of being empowered.	equal partners alongside government authorities."	
8. Inclusive and accessible	The approach is inclusive and increases accessibility to all those who engage with the method.	"Participants found this approach relatable" and "accessible to a wide range of stakeholder groups, including those with literacy or numeracy challenges, ensuring that no participants are disenfranchised during the research process."	"inclusive"; "accessible"; "reachable"
**Theme 3) Innovation and Creativity**			
9. Creativity and fun	Enables collective creativity, a sense of enjoyment, or fun	"enjoy the creative process of developing diverse scenarios" and "often leading to collective laughter."	"Creative"; "enjoy"; "fun"; "laugh"
10. Innovation or new ideas	Create new ideas, new knowledge, and innovations, or approach it innovatively.	"help participants expand their focus and identify a broader range of intervention options" and "an innovative way to collect detailed activity space data."	"innovate"; "idea"; "emerge"
11. Knowledge Integration	Bringing different pieces of knowledge together - engaging collective intelligence.	"provides a powerful means to access and transform clients' inner experiences" and "ensuring diverse perspectives are represented and considered."	"integrate"; "knowledge"; "perspectives"
**Theme 4) Well-being and Satisfaction**			
12. Improved well-being	Creates a sense of improved well-being, or improves the health or wellness of those involved.	"benefiting from therapeutic effects" and "This method can alleviate tension and apprehension in the research setting."	"well-being"; "wellness"; "therapeutic"; "relief"
13. Satisfied	There is a sense of satisfaction in the process or experience, or even with the outcomes	"high subjective satisfaction with the event" and "Participants appreciated their involvement."	"satisfied"; "appreciation"; "experience"
14. Trust	People involved trust the process, others, or even the outcomes.	"trusted the organizer" and "fostering trust."	"trust"; "trusted"
15. Motivation and inspiration	Increase motivation or inspire those involved.	"motivating residents to seek solutions" and "often perceived as inspiring by participants."	"motivation"; "motivated"; "inspired"

**Theme 5) Communication and Transparency**			
16. Enhanced communication	Execute processes or do something that enables or improves communication.	"allowing participants to explore and articulate their inner worlds in ways that traditional interviews may not facilitate" and "fostering rapport between researchers and community members."	"communication"; "communicate"; "understanding"; "articulate"
17. Transparency	Enables transparency or increases transparency about knowledge.	"transparency by providing structured opportunities for participants to express their opinions" and "offers transparency."	"Transparency"; "clear"; "clarity"
**Theme 6) Flexibility and Ease of Use**			
18. Flexibility	Discusses the adaptability, flexibility, or iterative nature.	"The methods used are often agile and flexible, not always requiring the constant presence of designers or architects" and "ease of modification."	"adapt"; "flexible"; "ease"
19. Simplicity	Makes things simple and easy.	"The PDR was praised for its concrete, manageable format" and "easy to use."	"simple"; "easy"
**Theme 7) Impactful and Valid**			
20. Impactful	What is created or executed has an impact.	"This method was particularly effective for both policy and firm frameworks" and "providing real-world applications and problem-solving opportunities."	"impact"; "effective"
21. Validity	Increases the validity of a process or the data.	"enhances data validity by involving all stakeholders in selecting and mapping community assets" and "rated highly for efficacy."	"valid"; "efficacy"; "validiy"
22. Skill development	Enables an increase in skill or knowledge.	"enhancing learning" and "It offers opportunities for co-researcher skill development."	"skills"; "learning"
**Theme 8) Reflection and Understanding**			
23. Reflection	Enables some form of self or collective reflection on the process, thoughts, ideas, etc.	"This method promotes self-directed learning, self-reflection" and "enables personal and social reflection"	"Reflect"; "mirror"
24. Understanding Complexity	Using processes or approaches to make complex things simple, or to understand complexity.	"making it a valuable tool for understanding and addressing complex social and environmental issues" and	"complex"; "comprehensive"; "understand"

		"providing a more authentic and comprehensive understanding than traditional methods."	
25. Visualization	There is some kind of visual representation.	"enhances the visual exploration and presentation of health data" and "creating large and visually appealing graphics."	"visualize"; "visual"; "represent"; "presentation"
**Theme 9) Efficiency and Strategy**			
26. Strategic	A strategic approach enables strategy or strategizing.	"ability to provide an aggregate or strategic view of problem structures help maintain focus on feedback loops rather than on details."	"strategic"; "strategy"
27. Efficient	the method is efficient or cost-effective	"is a cost-effective approach" and "making the process more efficient and comprehensive."	"efficient"; "effective;" "efficacy"; "cost-effective"
**Method Challenges (Q2)**			
**Theme 1) Engagement and Participation**			
1. Poor engagement	Captures instances of insufficient or ineffective participant involvement in research activities.	"which may limit the participants' engagement with the full scope of available qualitative data" and "Some participants, particularly adults, were reluctant to complete visual activities."	"limits engagement"; "limits participation"; "poor participation"; insufficient engagement"
2. Poor recruitment	Highlights issues related to participant recruitment.	"recruitment can be challenging, especially when using snowballing techniques" and "as reliance on village leadership and local research assistants affected participant selection."	"recruitment"; "poor recruitment"; "participant selection"
3. Unmotivated	Challenges related to people's motivation to engage or participate	"Additionally, if used in isolation, children may become bored with verbal interactions" and "which may limit participation unless individuals perceive a direct benefit for their work."	"not motivation"; "unmotivated"
4. Requires facilitation	Highlights instances where there is a need for skilled facilitation to achieve the aims.	"Participants often rely on guidance from project team members to identify issues for further exploration" and "if appropriate facilitators are available, the	"facilitation"; "facilitator"

		method's effectiveness is dependent on these conditions.”	
5. Requires skill or training	Emphasizes the necessity for specialized skills and training to effectively engage in certain research methods.	“Inadequate training can lead to errors, as participants may struggle with hypothesizing and linking variables effectively.” And “Proper training is necessary to utilize CLDs effectively.”	“inadequate training”; “required training”; “needs skill”; “needs training”
6. Group dynamics	Where people don't express their own opinions, but try to align with the consensus or the opinion of a leader in the group. Or about the way people interact, or engage with each other in the process.	“balancing researcher-participant dynamics.” And “These narratives can be influenced by those shared by other participants.”	“poor dynamics”; “shared ideas”; “bad interactions”; “poor interactions”; “negative particpant dynamics”
**Theme 2) Resources and Practical Constraints**			
7. Insufficient funds	instances where participants or activities face barriers due to a lack of necessary financial resources.	“insufficient funding to evaluate its effectiveness” and “resource-dependent.”	“insufficient funds”; “bad funding”; “resource-dependent”; “lack of funds”
8. Resource-intensive	Addresses the substantial resource requirements associated with certain research methods.	“requiring significant resources” and “the requirement for substantial resources for web-based Health impact assessment tools.”	“requires resources”; “substantial resources”
9. Logistical constraints	Captures the practical challenges and organizational hurdles that can impede the implementation of research methods and activities.	“Logistical considerations also arise, as facilitating art activities, especially in nature, can be messy and require careful planning.” And “scheduling, and conducting interviews, as well as producing and revising educational materials.”	“logistical constraints”; “practical challenges”; “scheduling issues”; “logisitical issues”
10. Time-intensive	Highlights methods that require significant time investment.	“Asset Mapping is time-intensive” and “Bayesian Networks (BNs) require time-intensive”	“time-intensive”; “time investment”; “requires time”
**Theme 3) Trust and Transparency**			
11. Distrust	Lack of trust.	“Additionally, participants may withhold information due to concerns about privacy or confidentiality.”	“distrust”; “lack of trust”; “withold”

12. Low transparency	Lack of transparency or clarity	"The method may also face challenges related to unclear outcomes or goals" and "Agent-Based Modeling (ABM) faces challenges such as low transparency, which raises doubts about its accuracy and effectiveness."	"lack of transparency"; "lack of clarity"; "low transparency"
13. Misunderstanding	Highlights instances where participants or researchers may misinterpret or misrepresent data or concepts, leading to confusion or inaccurate conclusions.	"involves challenges in interpretation" and "Very densely drawn sociograms can complicate interpretation."	"misunderstanding"; "complicated interpretations"; "challenges in interpretation"; "misrepresent"; "misinterpret"
14. Ethical concerns	The process raises concerns such as confidentiality and consent, ownership, or fairness.	"there are concerns about photo ownership, difficulties in capturing negative social concepts" and "as well as confidentiality and ownership issues."	"ethical concerns"; "concerns about confidentiality"; "unfair"
15. Emotionally draining	It causes negative emotions such as aggression, or people experience negative emotions or are emotionally drained due to the process.	"Draw and Write/Talk Technique can induce negative feelings" and "participant anxiety over taking "proper" photographs."	"negative emotions"; "emotionally draining"; "emotionally drained"; "aggression"; "anxiety"
**Theme 4) Methodological Limitations**			
16. Not robust	Identifies challenges related to the reliability and validity of research methods and data collection techniques.	"faces several challenges, including a lack of methods for systematically establishing traceability and validation" and "Convenience sampling could result in biases."	"not robust"; "challenges with validation"; "biases"; "poor reliability"
17. Not representative	Addresses concerns regarding the lack of representation in participant samples or data collection methods.	"participants may feel that the events do not capture a representative group of older people" and "may offer limited interaction space if migrant participants are excluded."	"not representative"; "lack of representation"; "limited interactions"; "participants are excluded"
18. Limited generalizability	Instances when you cannot generalize the findings	"which may limit generalizability" and "limits the generalizability of the results."	"limited generalizability"; "generalize the findings"
19. Limited data	This code refers to constraints and limitations in the scope and depth	"is limited to defining linear relationships between concepts" and "limited in-depth responses."	"limited scope"; "limited data"; "limited responses"; "constraints in depth"; "constraings of data"

	of data obtained from various methods and approaches.		
20. No evaluation	Captures instances where there is an absence or lack of comprehensive evaluation of methods or practices.	"The Five Whys Method is limited by its application to one youth participatory action research project within a school setting, which may affect its applicability to other settings" and "User stories are limited in improving quality, and there is a lack of empirical studies on their use and effectiveness."	"no evaluation"; "limited in quality"; "lack of effectiveness"
21. No impact	Instances where the method did not generate the intended outcomes or impact.	"not all centralities have a useful meaning" and "Pathways methods generated no uptake by the local government, which expressed little interest in the communities' input."	"no impact"; "no uptake"
**Theme 5) Systemic and Structural Barriers**			
22. System barriers	Addresses challenges inherent in using various methods that interact with systemic structures and geographic contexts.	"those whose activities span areas without available data may experience biased exposure estimates" and "Complex coding systems can be challenging to apply in real-time recording."	"integration"; "systems"; "bottom-up"; "geography"
23. Rigid	Identifies limitations related to inflexible research methods and frameworks that may not adequately adapt to dynamic or complex systems.	"are not well-suited for dynamic systems due to the exponential increase in computational burden with the number of variables" and "inability to handle uncertainty."	"inflexible"; "rigid"; "limit flexibility"
24. Inaccessible	Too challenging, advanced, or unsuitable for those engaging in the process. People struggle to access information or to engage.	"the public nature of these activities may exclude marginalized groups" and "Participants may struggle with reading maps or providing directions."	"inaccessible"; "not suitable"; "difficult to access"
**Theme 6) Focus and Commitment**			
25. Needs Commitment	Instances where there was a need for commitment from the people involved for it to be successful.	"The method requires a significant commitment from participants, researchers, and institutions" and "In some cases, it may necessitate guidance from a community advisory board."	"requires commitment"; "necessitates support"; "requires prioritization"

26. Topic drifting	Addresses the tendency for discussions or processes to veer off from the main focus	"the risk of losing the authentic voice of the child" and "cultural peculiarities can constrain their effectiveness, as participants may drift to side issues rather than focusing on the main process."	"drift"; "not focusing"; "lack of focus"
27. Disconnect	Asymmetry or disconnect between individuals, data, or systems.	"difficulties in attributing policy changes to specific interventions" and "limited community embedding."	"asymmetry"; "disconnected"; "difficulties integrating"
