# Supplementary material for: Co-creation methods for public health research — characteristics, benefits, and challenges: a Health CASCADE scoping review
Source: BMC Med Res Methodol. 2025 Mar 6;25:60. doi: 10.1186/s12874-025-02514-4 (PMC11884017; doi:10.1186/s12874-025-02514-4)
Supplement: Supplementary file 7 — Additional file 7. [file 12874_2025_2514_MOESM7_ESM.pdf]

Additional File 7. Benefits and Challenges of Each Method

Benefits of the Methods: 139 sources reported the benefits of 106 methods. The following are summaries of those benefits, referred to as the Pros of the method (in alphabetical order).

1. **Agent-Based Models (ABMs)** excel in representing complex spatial interactions and decentralized, autonomous decision-making processes. They are particularly effective in scenarios with heterogeneous conditions, allowing for detailed spatial and temporal representation. ABMs facilitate both qualitative and quantitative forecasting, and their flexibility makes them easy to modify and adapt. They support feedback loops and manage uncertainty well, providing a comprehensive understanding of dynamic systems. Additionally, ABMs promote knowledge integration and co-production by fostering robust, open processes that enhance communication among stakeholders. This approach improves the clarity of results and supports collaborative learning, making it a valuable tool for understanding and addressing complex social and environmental issues [1,2].
2. **Alternative scenarios** foster democratic processes and transparency by providing structured opportunities for participants to express their opinions, cast votes, and collectively review outcomes. It enhances participation, as participants engage actively and enjoy the creative process of developing diverse scenarios. This method stimulates critical thinking about uncertainties and vulnerabilities, encouraging discussions on dependence and proactive strategies for future preparedness. By exploring alternative futures, families, and communities can collaboratively identify preventative measures to mitigate economic uncertainties, building resilience and a shared understanding of potential challenges [3].
3. **Art making** provides a collaborative space where children can socialize, discuss meaningful topics, and share experiences. Group art activities foster communication and the exchange of ideas, creating a common ground for children to explore and express their unique interests. This interactive environment encourages creativity and peer engagement, allowing participants to connect through shared artistic expression [4]
4. An **Art-based narrative interview** provides a powerful means to access and transform clients' inner experiences, inviting them into a realm of phenomenological understanding that transcends verbal expression. This method promotes self-directed learning, self-reflection, and transformative growth, allowing participants to explore and articulate their inner worlds in ways that traditional interviews may not facilitate [5].
5. **Asset mapping** enhances data validity by involving all stakeholders in selecting and mapping community assets. This collaborative approach ensures that the data accurately reflects the community's strengths and resources, providing a more authentic and comprehensive understanding than traditional methods [6].

6. **Bayesian Networks (BNs)** effectively engage stakeholders by eliciting their knowledge and values and producing outputs in various formats. They are useful for summarizing information, focusing dialogue on key issues, and integrating both qualitative and quantitative knowledge. Their capacity for rapid updates and simulations promotes communication and learning, making BNs valuable for strategic considerations. Additionally, BNs are a helpful tool for assessing levels of knowledge and uncertainty [2,7].
7. A **blended approach of photovoice and photo-elicitation** captures complex experiences and perspectives, bridges cognitive limitations, and fosters creativity. Using interviews instead of group discussions also reduces stigmatization, creating a more inclusive and supportive environment for participants to share their insights [8].
8. **Body mapping** facilitates the exploration of thoughts, feelings, emotions, and vulnerabilities by providing a creative means to represent experiences that are challenging to articulate verbally. It helps participants reflect on their gender embodiment, dysphoria, and well-being, while also enabling them to share their insights and experiences with others in a meaningful way [9].
9. **Building a Model** offers significant advantages by allowing children to create models that reflect their unique interests and interactions with their environment. These models provide valuable insights into children's personal experiences with their local surroundings, capturing both past and present perspectives. Children's descriptions of their models reveal the social, cultural, geographical, and familial influences on their experiences of nature, highlighting the role fantasy plays in their environmental identity development [4].
10. The **Carer's Assembly** received positive feedback overall, with participants feeling heard and valued. Many reported that, for the first time as family carers, they felt they had a voice and were appreciated [10].
11. The **Causal Loop Diagrams** help participants expand their focus and identify a broader range of intervention options. It can inform the development of a logic model based on newly identified intervention components. CLD is simple to use due to its few conventions, making it accessible to non-technical audiences. Its simplicity and ability to provide an aggregate or strategic view of problem structures help maintain focus on feedback loops rather than on details. Additionally, CLD elicits personal stories and experiences, highlighting individual struggles and narratives [2,11,12].
12. **Checklist:** Participants found this approach relatable and appreciated the ranking exercise, as it encouraged deeper thinking about the issues. This method was particularly effective for both policy and firm frameworks [13].
13. The **Citizen's Jury** effectively includes "hard-to-reach" residents in decision-making processes, ensuring diverse perspectives are represented and considered. Most participants in the citizens' jury felt that the group was independent of the organizer and commissioner, trusted the organizer, and found the task clarity and access to resources satisfactory. Participants were comfortable with the level of conflict and the extent to which they listened to each other. The process was rated highly in efficacy and fairness, and participants reported an increased understanding of the issue and high subjective satisfaction with the event [14].

14. **The citizens' workshop** is effective in engaging diverse perspectives. Most participants felt that the workshop was independent of the organizer and commissioner and considered the organizer trustworthy. The clarity of the task and access to resources were judged positively. Participants were comfortable with the degree of conflict and the extent to which they listened to each other. The workshop process was rated highly for efficacy and fairness, with participants reporting an increased understanding of the issue and high subjective satisfaction with the event [14].
15. **Co-design by Appropriation of Affordances (Co-DAA)** involves an iterative, long-term approach that maintains a continuous exchange with stakeholders, fostering ownership and trust. This trust and ownership are crucial for motivating professionals to co-design cognitive tools in dynamic workplace settings and to embrace changes brought about by technology adoption. The co-design process evolves into a learning journey, enhancing both the development and integration of new practices [15].
16. **Cognitive mapping** is valuable for developing a shared understanding among participants, helping to align perspectives and clarify complex issues [2].
17. **Collaborative songwriting** offers a unique way to express experiences, providing participants with a sense of being heard and benefiting from therapeutic effects [16].
18. **Commentary charts** facilitate meaningful engagement in a "safe space," enhancing learning and generating new understandings. They embody democracy-in-action by promoting power and ownership among participants, leading to sustained engagement [17].
19. **Community mapping** can effectively engage youth not only as participants but also as facilitators of research, fostering openness and inclusiveness throughout the map-making process. It helps identify service needs and gaps, particularly for specific populations, by providing more nuanced information through workshops than traditional interviews or focus groups. Additionally, as residents discuss neighborhood problems and view data visualized in maps, they become motivated to seek solutions, stimulating action. This process also supports generating hypotheses and theorizing how community contexts influence health outcomes [16,18,19].
20. **Concept mapping** leverages mental models to provide insights into stakeholders' perceptions and serves as a basis for communication. It uses familiar images to help participants, such as immigrant residents, identify connections between factors affecting their neighborhoods. This method maintains a participatory and visual approach to research, systematically structures visual data quantitatively, and includes new community members without compromising results. Concept mapping fosters strong commitment and completion of the process, facilitates action for change, and enhances community engagement. It is an affordable method for assessing community needs, fostering rapport between researchers and community members, and organizing complex concepts for community planning. By combining qualitative and quantitative approaches, concept mapping increases research credibility and supports effective community involvement [18–20].

21. **Creative practice** explores research gaps and amplifies community voices to more influential actors. It serves as a method to address research shortcomings and ensure that community perspectives are heard by key stakeholders [21].
22. **Cultural consensus** is effective for communicating results clearly, handling uncertainty, and being easily modified. It offers transparency and supports both qualitative forecasting and dynamic temporal representation [2].
23. **The daily activity space travel diary** utilized an incentive-based completion approach, where compensation increased with each item completed, effectively minimizing respondent drop-out. This method applied spatial epidemiology in a low- and middle-income country setting and offered an innovative way to collect detailed activity space data, allowing for more precise risk exposure measures. These methods may apply to other high-risk populations and can inform broader health interventions [22].
24. **Daily work schedules** help determine community members' daily workloads and identify their routine activities, roles, and functions within 24 hours [23].
25. **Decision tree analyses** stimulate discussion among decision-makers, planners, and stakeholders by making uncertainty explicit, enhancing the transparency of the modeling process, and linking decisions to desired outcomes. This approach facilitates clearer communication and understanding of future actions. Decision trees are noted for their straightforwardness, ease of modification, and effectiveness in quantitative forecasting [2].
26. **Diamond ranking**, like picture sorting, effectively elicits preferences for specific aspects of a setting and requires participants to quantify these preferences. It also allows for collecting background reasons through annotations. Most participants were engaged and willing to participate in this activity, which stimulated discussion among them and with the facilitator. This method revealed a less obvious but significant aspect of participants' experiences with their surroundings, aiding in the development of aspirations for improvements, and reducing the power imbalance between researcher and participants [24,25].
27. **Direct ranking** fosters meaningful engagement in a "safe space," enhances learning, and generates new understandings. It exemplifies democracy-in-action by promoting power and ownership among participants, leading to sustained engagement. This method is accessible to a wide range of stakeholder groups, including those with literacy or numeracy challenges, ensuring that no participants are disenfranchised during the research process [17].
28. **A Dot map focus group** provides an easy way to gather community input on broad, place-related issues. The activity is interactive and accessible, allowing involvement from any community member regardless of educational background or literacy level [26].
29. **The draw and write technique** serves as a conversation starter, facilitating further discussions with children, ideally when participants have limited vocab. It enhances participation and expression of children's views, is not dependent on language, and is enjoyable, fun, and relaxing. This method reduces anxiety and helps build rapport. Drawings can be adjusted over time, researching sensitive issues: a critical appraisal

of 'draw-and-write' as a data collection technique acknowledging that a child's thoughts and emotions evolve. While it provides unique insights into participants' perceptions, it is most effective when combined with other methods for deeper interpretation and understanding [27–29].

30. **The draw and write/talk** method offers both academic and socioemotional benefits for participants. When used with children, researchers need to scaffold interventions based on the children's abilities, be conversant in their home language, and consider the research context to ensure effectiveness [30].
31. **Drawings** create a "universal language" that allows participants from diverse backgrounds and local languages to engage effectively in the research process. This method is inclusive, allowing all children to engage, regardless of artistic skill. It also provides participants with a sense of control, making it especially ideal for those with low literacy skills [31–33].
32. **Empathic Design** involves incorporating the perspectives of inhabitants to address social and cultural sustainability challenges in the design of affordable housing in developing countries. The methods used are often agile and flexible, not always requiring the constant presence of designers or architects. Building a connection with the community can form a strong foundation for achieving sustainable outcomes [34].
33. **Ethnographic Facilitation (EF)** incorporates a variety of data-gathering techniques, including participant observation, interviews, field notes, and document analysis, and applies methods such as constant comparison, grounded theory, and discourse tracing. EF focuses on solving real-world problems to improve lives and acknowledges the importance of addressing power dynamics and participation. The method embraces a participatory and collaborative approach, integrating group facilitation principles with ethnographic research. EF enhances traditional ethnographic and discourse analysis practices by facilitating interventions in group, organizational, and community communication, contributing to needs assessment and evaluation, and integrating various facilitation techniques within a long-term ethnographic framework. It provides a collaborative research model without necessarily adopting co-investigator roles and demonstrates how applied communication scholarship can benefit both scholarly and practitioner communities [35].
34. **The festival** as a research methodology provides valuable contexts for negotiating cultural identity and developing empowering knowledge. It addresses participants' lived conditions and experiences on both micro and macro levels, highlighting that all experiences are deeply embedded in social contexts [36].
35. **The Five Field Map** method facilitated interaction between the researcher and participating children, allowing them to express their feelings through the game [37].
36. **A Focus group** facilitates and evaluates group-based principles for action, stimulates dialogue, is culturally sensitive, and gains insights beyond one-on-one interview [38,39].
37. **Forum theatre** creates a safe space for physical movement within the group, often leading to collective laughter, recognition, and emotional release [40].
38. **The FUBI method** can be generalized to various application areas in Human-Computer Interaction, requiring primarily the use of one's own body [41].

39. **The future workshop** method provides a solid foundation for developing a shared understanding of future scenarios and common goals. It supports broad participation, encourages open discussion, and is often perceived as inspiring by participants [42].
40. **Fuzzy cognitive mapping** is a versatile and efficient tool for evaluating socio-environmental systems. It allows stakeholders to explore 'what if' scenarios, compare different interventions, and assess system trends. The method supports various construction approaches, integrating diverse qualitative data and experiences. With sufficient data, models can be generated using learning algorithms and analyzed through network metrics to reveal differing perspectives on a problem. Additionally, fuzzy cognitive mapping measures structural variations among stakeholders, providing valuable insights into uncertainty and the complexity of dynamic issues [2].
41. **Geographic Information Systems (GIS)** are valuable for examining spatial relationships in health research. They allow researchers to compare activity space areas with self-report survey data, revealing potentially significant links between psychosocial variables and geographic areas. GIS offers the opportunity to target resources and interventions geographically and enhances the visual exploration and presentation of health data. Key benefits include spatial representation, ease of communicating results, ease of modification, and transparency [2,19,43].
42. **Graphic facilitation** involves creating large and visually appealing graphics that can make complex information more engaging and memorable. The "trick" lies in the ability to use these visuals to impress and effectively communicate ideas [44].
43. **Graphs over time** enable non-static thinking by visually representing changes and trends, allowing for a dynamic understanding of data and its evolution [12].
44. **Informal interviews** facilitate free and natural discussion, capturing a child's unique perspective while avoiding rigid question-and-answer formats. They can be conducted spontaneously in natural settings, and occasionally, interactions among multiple children can provide particularly fruitful data [27].
45. **An interpretive focus group** enables discussion about sensitive topics by initially breaking into smaller groups, which provides a more comfortable setting for participants. This approach allows a spokesperson to report back to the larger group, ensuring that the opinions and experiences of those who may not feel comfortable speaking in a big group are still represented [45].
46. **Kitchen table talks** enable researchers to observe participants' inter-relational dynamics in the setting where the service is delivered and stimulate group discussions [46].
47. **Learner Verification and Revision (LV&R)** contributes to health communication by creating suitable health information and improving communication with intended audiences for new or adapted educational materials. This approach allows researchers, practitioners, and educators to identify and refine features in materials that need clarification and improvement. LV&R links communication theory with pedagogical practice to produce clear and effective materials for specific audiences. It has broad applications for developing health materials across both printed and digital platforms, including websites and apps, and can be extended to other topics and audiences [47].

48. **Living Lab** engages end users in the innovation process by creating a relevant work environment [48].
49. **Illustrative arts-based methodology (drawings)** empowers marginalized and stigmatized groups by allowing them to depict their realities, challenging traditional expert-driven narratives. This approach is adaptable to various practice settings, especially for social workers working with marginalized service users in culturally sensitive contexts. It leverages indirect communication through symbols and metaphors, which is valuable in collective societies where direct discussion might conflict with cultural norms [49].
50. **Mandala drawing**, as an arts-based method, offers several benefits. It is strengths-based and enjoyable for participants, providing a more expressive alternative to verbal communication. This method can alleviate tension and apprehension in the research setting and fosters a less hierarchical relationship between participants and researchers. Additionally, it facilitates an active process of meaning-making [50].
51. **The Mānoa mash-up** method facilitates participatory scenario building by helping participants navigate complexity through storytelling, allowing them to engage with their emotions and beliefs. It connects participants to their creative side and can be personally transformative, as some individuals develop a strong belief in their co-created vision of the future [51].
52. **The Metaplan method** fosters more effective group discussion and collects comprehensive information by saving time and ensuring the involvement of all participants in the group [52].
53. **Mind mapping** offers an advantage over standard note-taking by reflecting our natural, non-linear thinking patterns. This approach helps in organizing ideas more intuitively and visually, capturing the way thoughts and concepts are interconnected [53,54].
54. **Mockups of webpages**, whether hand-drawn sketches or wireframes created with tools like Balsamiq are presented at workshops to facilitate discussion. This allows for critical analysis and development of content and design ideas, enabling iterative refinement and enhancement [55].
55. **The Modified Delphi** fosters inclusive dialogue and reflexivity. Reflexivity involves critical reflection by researchers and participants on their paradigms and engages in a dynamic interaction between data and participants. This process leads to a more refined focus and deeper understanding [56].
56. **The MUST method** addresses common sustainability challenges in IT systems, fosters mutual learning between designers and end-users, and helps develop a coherent vision for projects. It offers tools and techniques for project management, and deals with ethical and practical issues related to users, aiding in the understanding, development, and presentation of relationships between IT and users' work practices [57].
57. **Narrative interviews** effectively capture the dynamic and temporal aspects of service experiences, including the spatial dimension beyond hospital settings. They provide deep and broad data on the spatial complexity and multiparty nature of child patients' service experiences. These interviews can also be useful for mapping children's

experiences and identifying influencing actors, as well as for exploratory studies focusing on children in sensitive contexts. They offer a practical alternative to methods requiring direct data collection from children [58].

58. **Participant observation** helps build relationships with study participants by allowing researchers to engage directly with them in their natural settings, fostering trust and understanding through immersive interaction [59,60].
59. **Participant photography** offers several benefits. It is an inclusive and flexible tool that addresses issues related to limited literacy, language barriers, or physical and developmental disabilities. This method is relatively inexpensive and was found to be both interesting and engaging for youth participants, making it a valuable addition to research [61].
60. **The Participatory Design Generator Cards** are user-friendly and accessible, even for those unfamiliar with the design process. They are perceived as interesting and inspiring by participants, with photo material and incentives fostering a common language among them. The cards facilitate the expression of knowledge and new ideas, leading design groups to identify practical ideas for planning new spaces. Simple solutions can be implemented immediately, and the cards are complemented by a user-friendly Internet tool [62].
61. **Participatory geographic mapping** is appreciated for its interactive nature, which engages participants actively. The methods used in this approach demonstrate the application of spatial epidemiology in low- and middle-income countries (LMICs) and help address challenges related to limited spatial data among vulnerable populations. This method not only gathers information but also promotes knowledge production and empowerment through participation. It enhances the community's understanding of the place and develops awareness about their surroundings. These methods may be generalizable to other high-risk populations and have broader applications for informing health interventions [22].
62. **Participatory Impact Pathways Analysis (PIPA)** facilitates extensive participation, often exceeding what is achieved with other methods such as Participatory Systems and Network Analysis (PSNA). It is comprehensive, addressing multiple issues, and offers flexibility in its application, making it adaptable to various contexts [63].
63. **Participatory mapping** allows individuals to create their own maps rather than using pre-drawn maps or census boundaries. This approach helps researchers understand which resources and activities are most significant to individuals' daily functioning and well-being. It helps re-frame citizens' knowledge in order to present it to technical experts for inclusion into governance [43,64].
64. **Participatory Theme Elicitation (PTE)** was found to be easy to understand and largely accessible for Youth Advisory Panel members, including a range of mixed-ability pupils. The activity was completed within the allotted time with minimal assistance from the research staff. PTE is designed to be accessible to both researchers and participants while minimizing the influence of researcher bias [65].
65. **Participatory Threats Assessment** often left participants enthusiastic about the findings and motivated to address the identified threats. Workshop facilitation teams

consistently reported that participants were eager to map their understanding and willing to quantify the threats [66].

66. **Participatory Video (PV)** provides individuals and communities with the opportunity to take a leading role in researching, developing awareness, reflecting on practices, and taking action. It fosters a collaborative approach to problem-solving, positioning participants as equal partners alongside government authorities. This method is increasingly valued for its ability to yield and validate community knowledge, guide policies, and programs to reduce social disparities, and improve communication between stakeholders. PV can enhance participatory communication by offering a powerful medium that reveals new perspectives, challenges existing perceptions, and stimulates creative processes. Aligning with other participatory arts-based methodologies, PV facilitates creative engagement and ensures direct community representation. It serves as an innovative tool that enables community-led engagement with a clear message and direction that embodies the voices of the entire community. It is easy and accessible and brings people together to explore issues, voice concerns, or be creative and tell stories. PV also uncovers important insights into the dynamics and contexts of people’s everyday experiences [67–69].
67. **Participatory visual methodology** enabled teachers to uncover new perceptions and understandings of their views on parental collaboration through video discussions. This approach engaged the teachers fully in the research process, making them active participants rather than mere subjects. It allowed for the exploration of sensitive topics in a non-threatening manner. Additionally, the process facilitated significant learning experiences for the teachers, including teamwork, video design and production, and using the final product to stimulate further discussions [70].
68. **Participatory/reflective photography** involves a structured coding scheme to identify common themes and patterns across a sample, capturing details for further textual analysis. This method encourages youth to explore their surroundings and enhances their connection with their community, whether they are familiar with it or not. It plays a crucial role in community engagement by providing real-world applications and problem-solving opportunities. Reflective photography offers several benefits: it conveys non-verbal content and triggers unforeseen interpretations, produces rich data with aesthetic and material dimensions, and captures a culturally significant mode of communication. It enhances participants' visual literacy, enables personal and social reflection, and treats participants as active, creative contributors, addressing power imbalances in the research relationship [71].
69. **The Partnership Data Report for Reflection (PDR)** offered valuable insights into the processes and outcomes of the CBPR model, linking past practices to current approaches. About 80% of respondents agreed that the PDR aligned with their values, and 70% found it easy to use. The PDR was praised for its concrete, manageable format, with one issue per page, and for supporting reflection on partnership processes. As a tangible tool, it was anticipated to serve as an iterative thought piece to be referenced throughout the project [72].

70. **Pathways** can aid in developing realistic solutions to problems and strategies for mitigation. They facilitate the creation of solutions by providing a structured approach to problem-solving and strategy development [3].
71. **Persona Building** can be utilized as a tool to navigate interventions from a specific perspective, offering insights into the needs and experiences of particular user profiles [73].
72. **Personas** can capture and communicate different customer categories, facilitating the development of various service design scenarios. The persona technique focuses on users and their experiences, providing insights into the lived experience beyond isolated service encounters. It has proven to be a valuable tool for identifying relevant themes for target groups in public sector environments, offering more comprehensive information than simple observational methods [74].
73. **Photo-elicitation** is a powerful method in co-creation due to its ability to evoke a broad spectrum of ideas and insights from participants, often capturing information that might otherwise be difficult to articulate. By using photographs, this method bridges gaps between researchers and participants, facilitating a focused dialogue and addressing potential communication barriers. Its inclusive nature allows diverse stakeholders to express their viewpoints, making it particularly effective in engaging various groups, including those with differing literacy levels, such as younger children. Additionally, photo-elicitation is valuable in working with vulnerable or frail populations who might not normally participate in research, as it does not rely solely on verbal communication. The visual stimuli provided by photographs stimulate discussion and reflection, enabling participants to convey practical knowledge, perceptions, and values associated with specific behaviors or phenomena. This approach fosters a collaborative environment where participants and researchers can jointly explore and understand their physical, social, and cultural contexts, enhancing the depth and quality of the data collected [24,44,75–78].
74. **Photo production with interviews** is particularly beneficial as it does not rely solely on language, making it accessible for vulnerable or frail populations who might otherwise be excluded from research [79].
75. **Photo walkabouts** are a visual method that encourages participant collaboration while accessing their experiences and voices, enhancing the depth and richness of the research, and has the potential to gain in-depth perspectives from the target group [46,80].
76. **Photovoice** is a powerful method for capturing and communicating community perspectives on significant changes and issues. It complements traditional evaluation methods by offering a rich, visual layer of insight that enhances understanding of local health and social issues. This method engages individuals, especially those with limited experience or confidence in research, by providing them with a platform for self-expression and advocacy through photography. It helps reveal deeper psychological layers and contextual understanding, facilitating dialogue and action that can lead to social change. Photovoice is especially effective in capturing both tangible and intangible aspects of life, bridging gaps between physical and social environments. It fosters community-oriented solutions, promotes collective action,

and allows for nuanced exploration of behaviors and their impacts. The method supports the development of critical knowledge and dialogue, enhances visual literacy, and addresses power imbalances, making it suitable for diverse and marginalized populations. Additionally, Photovoice is adaptable for online platforms, which became increasingly relevant during the pandemic. It offers opportunities for co-researcher skill development, including photography, critical reflection, and political engagement, and is a cost-effective approach that can be integrated with other methods to enrich research outcomes [40,68,75,81–119].

77. **Playback Theatre** involves a collective storytelling process where participants' stories are interconnected and built upon each other. This approach reflects participants' constitutive experiences and identity constructions in a non-judgmental manner. Unlike traditional interviews, which often yield detailed chronological accounts or normative perceptions, Playback Theatre generates unique themes and moments that might not otherwise surface. It fosters a dynamic and reflective environment where personal experiences are shared and explored collectively [40].
78. **PowerView** empowers students by inverting traditional power dynamics, placing them in a position of authority over the researcher. This shift in power relations allows students to have greater control and influence over the research process [120].
79. **Prototyping** facilitates communication by clarifying and refining ideas, providing tangible models for participants to interact with, and enhancing dialogue and understanding [73].
80. **Purposive sampling** aims to maintain rigor by identifying a sampling frame based on specific study-driven variables or characteristics [121].
81. **Q sorting (paper-based sorting approach)** involves re-examining the results, which enhances the rigor of the method [122].
82. **Rapid HIA** is a versatile tool that can be applied at various stages of the planning process to provide meaningful feedback for planning, design, evaluation, and mitigation. It is supported by guidebooks and manuals shaped by user feedback and relies on research evidence to ensure relevance. The approach integrates diverse forms of knowledge, including local and expert insights, and makes evidence accessible through web resources. Rapid HIA accommodates different timeframes, planning types, and staff capacities, and helps manage controversies by offering new perspectives. It addresses a wide range of health topics while accounting for uneven access to local health data and relies on established GIS data and expertise [123].
83. **Reflective drawing** is a valuable method for generating data directly from participants, rather than relying on designers to interpret conversations. It merges the processes of iteration and reflection, providing a cohesive approach to understanding participant perspectives. This technique reveals the relationship between people and their environment and uncovers emotional dynamics that might be difficult to express through writing [124].
84. **Role-playing** is a beneficial data collection method, particularly for children, as it is perceived as fun and engaging. It offers flexibility, allowing storylines to adapt to both group consensus and individual ideas. The method is somewhat structured and

focused, promoting social engagement among peers. When conducted outdoors, role-playing encourages children to connect their stories with their environment [4].

85. **Role-Playing Games (RPGs)** facilitate participative discussions in conflict regions, allowing for the collection of information without physically visiting subregions. They help verify social, economic, and environmental dynamics and identify interactions among players. RPGs are described as "dense methodological tools," meaning they are designed to synthesize complex systems into a cohesive format [125].
86. **The SAT's Competence Tool** is valuable for empowering communities to self-assess their competence and track their progress. It facilitates self-assessment of needs, fosters dialogue, provokes thought, and inspires communities to enhance their capacity to address challenges related to HIV and AIDS [126].
87. **Semi-structured interviews** offer interviewers flexibility to explore topics in-depth with participants and capture a generalized view of experiences, particularly in healthcare settings [58,59].
88. **Snowball sampling** is efficient and often yields higher attendance rates compared to purposive sampling. It effectively recruits larger numbers of eligible stakeholders, particularly in hard-to-reach or hidden populations, by leveraging established social networks. Snowball sampling can lead to higher engagement and credibility in community research and mixed methods studies [121].
89. **Social Network Analysis (SNA)** is effective in highlighting stakeholder behavior, revealing new and urgent topics, and uncovering sub-topics. It facilitates crowdsourcing to identify a broad range of stakeholders and is not limited to specific organizations. SNA helps to map local stakeholder ecosystems, assess priorities across a large number of stakeholders, and identify emergent features within networks. Once the ecosystem is established, it allows for the evaluation of numerous organizations [2,127].
90. **Sociogram (directed graph):** The immediacy of capturing events through hand-drawn sociograms allows participants to engage in interpreting the data immediately, enhancing participation and reducing recall inaccuracies. Sociograms made visible the patterns of interaction between health professionals and families, stimulating dialogue about communication and practice. The drawing process itself often sparked curiosity among participants, initiating discussions on communication patterns depicted [44].
91. **Spatial access priority mapping** benefited from an excellent response rate, with fishers eagerly sharing diverse information and appreciating the opportunity to express their views. The research's on-site approach, conducted on fishers' boats, at the quayside, or in local cafes, minimized the costs of participation and offered convenience. Utilizing digitized maps provided flexibility for interviews, enabling the use of multiple maps at varying scales [128].
92. **Stakeholder analysis** increases the visibility of issues, clarifies how stakeholder values and objectives converge, and provides a foundation for reforming participation and decision-making processes. It offers a deeper understanding of stakeholders' perceptions of existing conflicts, uncovers hidden areas of tension, and gives stakeholders a platform to voice concerns. Profiling and mapping stakeholder

attributes aid in conflict resolution and prevention. This method is versatile and applicable across various research fields, including forestry and natural resource management, by analyzing stakeholder interests and participation in governance processes [129–131].

93. **The stick-a-star quiz** is effective in engaging younger children by providing them with active tasks rather than passive participation. These techniques stimulated interactive dialogue and minimized power imbalances between the adult researcher and child participants. They helped maintain motivation and attention, supported focused verbal interactions, and provided structural guidance, all while shifting focus away from the researchers and recognizing individual interests [27].
94. **Storyboards and animations** encourage participants to engage actively [48].
95. **Storytelling** helps researchers understand the influence of language and culture on research and provides strategies to minimize cross-cultural barriers in the research process[132,133].
96. **Strategic Environmental Assessment (SEA)** aims to transform decision-making by incorporating environmental values and adopting a multidimensional perspective. This approach enhances decision-making by integrating diverse environmental considerations rather than relying on a single-disciplinary viewpoint. By providing decision-makers with comprehensive and relevant information, SEA ensures that environmental impacts are effectively integrated into the planning process, ultimately adding significant value compared to traditional assessments [134].
97. **Structured surveys** benefit significantly from the increased availability of tablets and low-end smartphones, which have addressed barriers to electronic data collection. Smartphones enhance structured surveys by enabling the collection of a wide range of data, including location, multimedia (photos, videos, and audio), and text. This advancement improves data visualization, accuracy, and analysis, making the process more efficient and comprehensive [2].
98. **The Five Whys Method** effectively reveals root causes by highlighting more structural attributions compared to individual attributions. This outcome underscores the method's strength in identifying underlying issues and provides valuable insights into problem-solving [135].
99. **Threshold Analysis** is a versatile tool used throughout various stages of the planning process, offering valuable feedback for planning, design, evaluation, and mitigation. It is supported by guidebooks and manuals informed by user feedback and relies on research evidence to select measures that balance evidence with policy or planning relevance. The method integrates diverse forms of knowledge, including local insights through rapid assessment and expert knowledge in other tools, with the website providing broad access to evidence. It effectively addresses controversies by raising concerns and offering new perspectives, covers a wide range of health topics, and accounts for uneven access to local health data. Threshold Analysis also utilizes well-established GIS data and expertise [123].
100. **Transdisciplinarity (TD)** operates on the principle that the knowledge and experience of all participants are considered equally valuable. This approach

emphasizes the equal contribution of diverse perspectives in the collaborative process [136].

101. **User-driven systematic reviews** enhance the relevance and quality of the review process by incorporating user input and perspectives, ensuring that the findings are more aligned with the needs and priorities of the intended audience [137].

102. **The video diary method** used with child patients, along with narrative interviews with their parents, effectively captures the dynamic nature of service experiences and the broad temporal dimension. It provides rich, subjective data on the daily life and surroundings of child patients, offering valuable insights into their experiences and perspectives. Using video diaries or narrative methods with individuals, including children and adults, can address issues by capturing patient perspectives and information that extends beyond the service setting [58].

103. **The Visioning Tool**, as part of the CBPR model, received high approval from 80% of respondents, who found it beneficial and noted its advantages over current practices. Approximately half of the respondents found it easy to use and expressed a likelihood of using it again. The tool allowed teams to develop their models, leading to new insights into their partnerships, desired outcomes, and collaborative practices. It was particularly useful in addressing power differentials, exploring new possibilities in long-term partnerships, and supporting brainstorming and foundational work in newly formed partnerships [72].

104. **The Visual Voices method** enhances integrated knowledge transfer by involving adolescent participants in analyzing, interpreting, and summarizing both multimedia and traditional qualitative data. This participatory approach also informed the planning of three primary dissemination sessions, ensuring that the findings were effectively communicated and relevant to the participants' perspectives [138].

105. **The Youth ReACT data analysis method** enabled authors to produce a comprehensive analysis of current community conditions and develop substantial action recommendations from youth perspectives. The success of this in-depth analysis and actionable insights is attributed to the effectiveness of the method used [139].

106. **The Zaltman Metaphor Elicitation Technique (ZMET)** proved effective in uncovering both conscious and unconscious thoughts and feelings, which contributed to its adoption. The technique's ability to reveal latent and nuanced dimensions of consumer value and value co-creation in healthcare contexts was particularly valuable. ZMET facilitated a deeper understanding of complex concepts such as quality of care and treatment efficiency, with themes emerging and evolving through visual elicitation. The use of evocative images allowed participants to explore and articulate higher-order constructs and personal healthcare experiences. Participants appreciated their involvement, especially in the visual elicitation phase, finding it enjoyable and meaningful. The simplified version of ZMET used in the second phase of interviews was noted for its efficiency and ease of implementation [140].

Method Challenges: 90 sources reported the challenges of using 78 methods. The following are summaries of those potential pitfalls, referred to as the Cons of the method (in alphabetical order).

1. **Agent-Based Modeling (ABM**) faces challenges such as low transparency, which raises doubts about its accuracy and effectiveness[1,2].
2. **Alternative Scenarios** face challenges such as the need for skilled facilitators who can maintain focused discussions while allowing free expression of ideas. Additionally, the public nature of these activities may exclude marginalized groups, though this can be somewhat mitigated by adapting the activities to enhance personal expression [3].
3. **Art Making** methods face several challenges, including the potential for social influence, where children may mimic the artwork of others or create art that aligns with perceived expectations of the researcher rather than their authentic ideas. Additionally, some children might produce artwork that seems unrelated to the environmental context, though these creations should not be dismissed but rather explored for their intended connections. Logistical considerations also arise, as facilitating art activities, especially in nature, can be messy and require careful planning [4].
4. **Asset Mapping** is time-intensive and demands extensive training and oversight to be effective [6].
5. **Bayesian Networks (BNs)** require time-intensive training to master and are not well-suited for dynamic systems due to the exponential increase in computational burden with the number of variables. Additionally, cultural peculiarities can constrain their effectiveness, as participants may drift to side issues rather than focusing on the main process [2,7].
6. The **Blended Approach of Photovoice and Photo-Elicitation** may be too advanced for some individuals with complex health needs, posing a challenge for effective application [8].
7. **Body Mapping** faces several challenges, including the potential awkwardness for participants with arts-informed methods. Additionally, lack of time and funding can create interconnected difficulties, and recruitment can be challenging, especially when using snowballing techniques [8].
8. **Building a Model** method has notable disadvantages. Children's incorporation of both real and imaginary elements may obscure the true representation of their lived experiences. Additionally, the method may not be suitable for very young children who are still developing fine motor skills, as they might find it challenging to use liquid glue or cut and shape materials effectively [4].
9. **Causal Loop Diagrams (CLDs)** face several disadvantages. Inadequate training can lead to errors, as participants may struggle with hypothesizing and linking variables effectively. CLDs may represent flawed mental models and can introduce ambiguity if loop polarity is not labeled correctly. They do not distinguish between variables that accumulate or decrease in number, which can result in flawed inferences about problem dynamics. Additionally, CLDs require a shared understanding of variable

definitions and system functioning among group members, or else they risk producing shallow diagrams that overlook significant insights and differences in stakeholder perspectives. Proper training is necessary to utilize CLDs effectively, though it comes with associated costs [2,11].

10. **Checklists** face challenges such as working less effectively in certain agency environments. Participants often rely on guidance from project team members to identify issues for further exploration. Additionally, the application of checklists typically follows a top-down sequence, which may limit the flexibility and comprehensiveness of the method [13].
11. **Citizens' Jury** may face challenges such as being too expensive and time-consuming. Additionally, participants may feel that the events do not capture a representative group of older people [141].
12. **Citizens' Workshop** may face criticism for not capturing a representative group of older people, as noted by participants in the workshop [141].
13. **Co-design by Appropriation of Affordances (Co-DAA)** faces several challenges, including a lack of methods for systematically establishing traceability and validation. It is primarily supported by a single case in workplace learning and places additional demands on professionals' schedules [15].
14. **Collaborative Songwriting** can be time- and resource-dependent as children may initially be shy and require encouragement to participate effectively [16].
15. **Commentary Charts** can be slow and labor-intensive. While they are useful and provide a valuable learning experience, the process may make some participants feel like outsiders if they are not involved in daily practice [17].
16. **Concept Mapping** has several challenges. It provides only a snapshot of neighborhood characteristics, which may change over time. Convenience sampling could result in biases, as those who chose to participate might differ from those who did not. The method requires a significant commitment from participants, researchers, and institutions, which may limit its feasibility in some community-based participatory research (CBPR) contexts. Additionally, Concept Mapping lacks robust statistical mechanisms, like factor analysis, to reduce the number of statements generated. The structured nature of brainstorming sessions may lead to the loss of in-depth information that might be uncovered in less structured settings [142–147].
17. **Consensus Conference** faces challenges such as imbalances between parties and asymmetry in knowledge due to the lack of shared background materials before the conference. Additionally, the model has been modified to reduce the duration of meetings, which may impact its effectiveness [148].
18. **Creative Practice** faces several challenges, including insufficient funding to evaluate its effectiveness and limited community embedding, as reliance on village leadership and local research assistants affected participant selection and communication. Interaction with policymakers was restricted to a few exchanges, and there was insufficient time to integrate the approach into the decision-making process or combine it with conventional drought measures. Additionally, the project lacked the time to evaluate the long-term benefits of the creative practice approach [21].

19. **Daily Activity Space Travel Diary** faces several challenges. Participants often struggle with writing and recording exact addresses due to unfamiliarity with street names. Although participants expressed openness to using GPS tracking in future studies, the current method relies on self-reported addresses and Google Maps, leading to potential spatial imprecision and errors. Additionally, the time spent at each location is subject to recall and reporting bias [22].
20. **Decision Tree Analyses (DTA)** face several limitations, including poor handling of uncertainty, inability to support feedback loops, and limited qualitative forecasting. Additionally, DTA provides an inadequate spatial representation [2].
21. **Dialogic Art (Relational Art)** faces challenges including its application as a case study in a specific region with self-selecting participants, which may limit generalizability. Although the models of participation described are readily replicable if appropriate facilitators are available, the method's effectiveness is dependent on these conditions [149].
22. **Diamond Ranking** faces challenges such as reluctance from some participants, like the groundsman and a group of technicians, who preferred their views to be recorded rather than engaging with the activity. This reluctance led to difficulties integrating their opinions into later stages of analysis, as their views did not align with those generated by other participants through the activities [24,25].
23. **Direct Ranking** can be slow and labor-intensive. While useful, they may involve lengthy presentations and may leave some participants feeling like outsiders, particularly if they are not involved in daily practice. The method may also face challenges related to unclear outcomes or goals, such as the benefits of assessing guidelines and training initiatives, and may offer limited interaction space if migrant participants are excluded [17].
24. **Draw and Write Technique** presents several challenges, including the need for specific knowledge and skill, as participants may only depict what is easy for them rather than abstract concepts, which can be taxing. The method also raises issues of confidentiality and ownership, can be emotionally draining for participants, and involves challenges in interpretation [27,28].
25. **Draw and Write/Talk Technique** can induce negative feelings during the writing and narrative explanation process. Therefore, it requires careful and sensitive handling by the researcher to manage participants' emotional responses effectively [30].
26. **Empathic Design** requires significant organization and time investment. The process can be time-consuming, which may pose challenges in managing and executing the design effectively [34].
27. **Festival** faces considerable debate regarding the nature of knowledge produced, as they rely on qualitative research methods using textual and multi-modal data rather than numerical data. This can lead to questions about the validity and interpretation of the findings [36].
28. **Forum Theatre** may not be suitable for all situations of social conflict. There are instances where the scenarios selected by participants are excessively repressive or aggressive, leaving no room for productive negotiation or resolution [40].

29. **Future Workshop** often produce findings that are quite general, making it difficult to achieve concrete results within the workshop itself. The results typically require further reprocessing and are only the initial phase of a change process, necessitating additional measures to fully implement and realize outcomes [62].
30. **Fuzzy Cognitive Mapping (FCM)** faces several challenges: it does not represent specific quantities and is limited to defining linear relationships between concepts, lacking the ability to capture time or delays as the system evolves in discrete steps rather than real-world time. The model output is restricted to conceptual and qualitative units without real-time reference, making it difficult to assess how system dimensions change over desired time horizons for decision-making. Additionally, FCM lacks spatial capability [2].
31. **Focus Groups** may face limitations as traditional research designs and conceptual frameworks often fall within narrow boundaries defined by existing academic literature. Experiences from fieldwork indicate a need to extend these definitions to include creative and activity-based approaches, which are not adequately covered by conventional focus group methods [150].
32. **Geographic Information Systems (GIS)** can present challenges in assessing community integration. The method may underestimate integration if activity locations are clustered close together or overestimate it if an activity is infrequent but far from home. Additionally, activity space methods may not provide easily interpretable indices of community integration across different study locations, such as metropolitan versus rural settings. Using GIS for health investigations can be complicated by limitations in accessing confidential health data at a fine geographical scale. Furthermore, implementation of quantitative GIS models typically requires a high level of technical skill, and over-reliance on technical aspects of GIS may alienate less-skilled stakeholders. GIS also faces limitations such as poor temporal representation, poor qualitative and quantitative forecasts, inability to handle uncertainty, and lack of support for feedback loops [2,19,43].
33. **Graphic Facilitation** requires a degree of artistic skill to create graphic records, which can be a challenge for some practitioners. However, considerable guidance on using icons, calligraphy, color, and other techniques is available for those wishing to develop their graphic competence [44].
34. **Informal interviews** with children can face challenges related to parental presence, as parents might either constrain the child's participation or aid in building trust and communication with the researcher. Additionally, if used in isolation, children may become bored with verbal interactions, leading to reluctance to talk and limited in-depth responses. Challenges also include confidentiality issues and the risk of losing the authentic voice of the child [27].
35. **The interpretive focus group** presents challenges including its reliance on the researcher's prior analysis of existing data, which may limit the participants' engagement with the full scope of available qualitative data. Additionally, participants only engage with a small portion of the data, potentially affecting the depth of analysis. Time constraints also pose challenges, impacting the thoroughness of the process [45].

36. **Learner Verification and Revision (LV&R)** requires significant time and planning, including the creation of interview guides, participant recruitment, scheduling, and conducting interviews, as well as producing and revising educational materials. This method involves costs related to staff time, participant incentives, and material production. A thorough understanding of the interviewer's guide and experience in establishing rapport is essential for success. Participants may struggle to envision the end product, and a careful review of data from each iteration is necessary to uncover new insights. Establishing a comfortable environment and involving advisors early and continuously are crucial for enhancing the quality of the materials and interventions [47].
37. **Illustrative Arts-Based Methodology (Drawings)** presents challenges as arts-based self-expression can be a Westernized concept, potentially unfamiliar and uncomfortable for participants, much like questionnaires and interviews. Additionally, participants with backgrounds in welfare and therapy might fear that the art is being used to 'diagnose' or 'analyze' them, which could affect their engagement with the method [49].
38. **Mandala Drawing** can present challenges, including participants experiencing anxiety about drawing or fear of negative judgment. Some might view the activity as childish. Additionally, instructors (researchers) need to be trained in arts-based methods, requiring expert training for effective implementation [50].
39. The **Mānoa Mash-Up Method** for facilitating participatory scenario building can be very time-intensive, which may limit participation unless individuals perceive a direct benefit for their work. The method can also face potential constraints in engaging diverse groups and is highly dependent on the participants invited and the seed initiatives chosen. It is best suited for specific circumstances where creative and radical future visions are needed [51].
40. **Narrative Interview** has limitations including the inherent unverifiability of secondhand accounts and observations of actual encounters. Additionally, some parents found it difficult to review their experiences [58].
41. **Participant Photography** presents challenges in both ethical considerations and practical application. Time constraints can limit the completion of photography projects, affecting both the duration for participants to collect images and the sessions available for discussing the photographs. The costs associated with this approach may also be prohibitive for some researchers. Additionally, self-censorship is a concern, as participants may make conscious and unconscious choices during the act of taking photographs [61].
42. **Participatory Design Generator Cards** require a skilled facilitator to guide discussions beyond initial examples and tailor the application to specific contexts. The large set of cards can be time-consuming to work through. Additionally, the cards may become outdated unless they are regularly updated [62].
43. **Participatory Geographic Mapping** faces challenges due to limitations in locating points or areas caused by outdated or limited satellite imagery, such as that of Santo Domingo on Google Maps. Participants may struggle with reading maps or providing directions, and those whose activities span areas without available data may

experience biased exposure estimates. Additionally, spatial imprecision and error are likely because data are based on participant-reported addresses rather than GPS tracking [22].

44. **Participatory Impact Pathways Analysis (PIPA)** is very time-consuming and can be perceived as such even when shortened, which may affect participant engagement and overall effectiveness [63].
45. **Participatory Mapping** can face limitations in representation if the sample size is small and may not always provide exact locations for each activity [43,64].
46. **Participatory Threats Assessment** requires careful handling of data on stakeholders' perceptions, as these can reflect both personal experiences and stories from others, potentially exaggerating the likelihood of dramatic events. Additionally, this approach may capture threats over broad regions and extended timeframes. It is crucial to gather accurate perceptions to avoid proposing interventions that do not align with stakeholders' views, which could lead to opposition [66].
47. **Participatory/Reflective Photography** can face challenges such as the development of detailed quasi-quantitative frameworks for analyzing photographs, which may seem excessive given the varied sophistication and artistic merit of images. Despite the proliferation of digital technologies, visual research remains marginal in practice, with many scholars unaware of its potential. The lack of rigorous methodology and specificity in studies can reinforce prejudices about the ambiguous nature of images as data. Additionally, the field's focus on textual research culture may hinder the adoption of visual methods. However, visual research can offer rich and unique insights if conducted with rigor, transparency, and reflexivity [71].
48. **Pathways** methods generated no uptake by the local government, which expressed little interest in the communities' input [3].
49. **Photo-elicitation:** Photo-elicitation can be time-consuming for both researchers and participants, requiring significant resources. Some participants, particularly adults, were reluctant to complete visual activities, which may indicate a tendency to overlook their involvement. The method's effectiveness can be limited by delays in commenting on photographs, which may lead to difficulties in recalling original thoughts. Training provided to participants might influence their choices and affect the results. The method may not always be appropriate or accepted by all participants, and the use of complex equipment, like digital cameras, may deter engagement. The study represents a snapshot in time, lacking longitudinal insights into changes in perceptions or behavior [24,44,76–78,151].
50. **Photovoice** can be time-consuming and expensive, particularly with large participant groups and multiple time points. Challenges include the risk of overlooking adult perspectives, difficulties in attributing policy changes to specific interventions, and potential non-representativeness of the viewpoints reflected. Ethical concerns involve the need for extensive consent forms, handling sensitive imagery, and addressing stigmatization risks. Practical issues include complications with camera use, photo selection, and balancing researcher-participant dynamics. Additionally, there are concerns about photo ownership, difficulties in capturing negative social concepts, participant anxiety over taking "proper" photographs, and the influence of researchers

on photo selection. Technological issues, such as limited participant skills and reliance on regular engagement, further complicate implementation [81,83,84,86,87,89,94–96,98,100,101,103–112,114–116,118,152–154].

51. **Playback Theatre** often produces incomplete narratives, omitting much of participants' daily life experiences. These narratives can be influenced by those shared by other participants and cannot replace long-term ethnographic or participant observation studies [40].
52. **Purposive Sampling** requires collaboration to identify participants matching specific characteristics, which can be time-consuming. It may be challenging to implement if there is a lack of cooperation from employers or other key stakeholders, potentially limiting access to diverse community groups. In some cases, it may necessitate guidance from a community advisory board and a shift to convenience sampling to address recruitment challenges [121].
53. **Plan Review Checklists** require further testing for reliability and validity. Additional testing is needed beyond the development team to ensure different users produce consistent results. The checklists should be tested for reliability to verify if various individuals arrive at the same conclusions. While the tool has face validity—appearing logical or sensible to experts—testing their predictive accuracy for health consequences remains challenging. More extensive evaluation in different contexts would enhance the tools' effectiveness and applicability [123].
54. **Rapid HIA** can be challenging to initiate discussions among participants. Key difficulties include encouraging users to prioritize the collection and dissemination of information about the project and related health issues before the workshop, and ensuring sponsors provide accessible versions of the results [123].
55. **Role-playing** often has limitations as storylines can be constrained by the props available to children [4].
56. **Role-Playing Games (RPG)** can be challenging due to the difficulty of recording all information generated by the synchronicity of events and the high volume of data. Additionally, the time available for the game may be insufficient to play the desired number of rounds [125].
57. **SAT's Competence Tool (SATCOMP)** is limited by its reliance on reviewing the perceptions of a community, which are translated into qualitative results [126].
58. **Semi-participant observations** raise concerns about the observer's effect on the observed, as their presence can disturb the natural environment and alter typical human behavior [27].
59. **Semi-structured interviews** may concentrate on specific periods, such as hospitalizations, and provide limited insight into patient-led experiences. Additionally, healthcare professionals may exhibit bias in describing their impact on patients' experiences [58].
60. **Snowball sampling** may be ineffective in identifying diverse individuals and might only locate participants with specific characteristics. Additionally, participants may withhold information due to concerns about privacy or confidentiality, particularly in qualitative studies [121].

61. **Social Network Analysis (SNA)** can be challenging due to difficulties in engaging a statistically significant group, explaining the meaning of centrality degrees for topics, and properly weighting relationships. It also requires a significant amount of time to map many stakeholders, and not all centralities have a useful meaning [127].
62. **Sociogram (directed graph)** has limitations including the reduction of a visual web of interactions to a numerical tally, which may not be very instructive. Early sociograms produced by researchers were densely drawn, making interpretation and analysis difficult. Accurate charting requires attention to patterns and intentions of interaction without getting caught up in the content or meaning during recording. Very densely drawn sociograms can complicate interpretation, and it's often better to use new sheets of paper for lengthy or complex interactions. Complex coding systems can be challenging to apply in real-time recording, whereas a simple color key is more practical and supports consistency between recorders [44].
63. **Stakeholder Analysis** varies in its limitations depending on the sub-method used. It has been criticized for weaknesses related to analytic, qualitative, and academic rigor [130,131].
64. **Stick-a-Star Quiz** has limitations including providing limited in-depth data, challenges related to exploring issues raised, context difficulties such as interruptions and time limits, as well as confidentiality and ownership issues [27].
65. **Strategic Environmental Assessment (SEA)** faces technical and institutional challenges including significant differences between sectors and decision-making levels within countries, the lack of formal decision-making procedures for many policies, programs, and plans, the extensive geographic scope of SEA, the complexity of data collection and analysis due to numerous alternatives, uncertainty about future environmental, technological, economic, and social conditions, and limited information or incompatible data about SEA [134].
66. **Structured surveys** face challenges with managing and processing paper-based survey data, particularly when dealing with large sample sizes [2].
67. **The Five Whys Method** is limited by its application to one youth participatory action research project within a school setting, which may affect its applicability to other settings [135].
68. **Threshold Analysis** faces challenges such as confusion in determining distances for measuring performance, the need for geographic information system knowledge which varies among agencies, and the requirement for substantial resources for web-based Health impact assessment tools. Additionally, while the tools have face validity, they need more extensive testing for reliability and validity beyond the development team [123].
69. **Transdisciplinarity (TD)** processes are complex and dynamic, with partnerships and their interactions being shaped by the problem being addressed. In cross-cultural settings, TD faces additional challenges. The method lacks general procedures for achieving reflexivity, though efforts have been made to characterize successful collaborations. Budgetary discussions in intercultural TD also present challenges due to differing definitions of "value," "compensation," and "fairness," which can be difficult to negotiate and integrate [136].

70. **User-driven systematic reviews** may face challenges in achieving a representative composition of co-creators [137].
71. **The video diary method** may present challenges such as high rejection rates, with 20 of 34 contacted children declining due to lack of time, strength, or motivation, potentially leading to biased data as only highly motivated participants remain. Additionally, completing the video diary can be time-consuming [58].
72. **User stories** are limited in improving quality, and there is a lack of empirical studies on their use and effectiveness [155].
73. **Visioning with the CBPR model** can be complex and challenging, as some respondents found the text to be dense [72].
74. **Visioning** methods in Vietnam face limitations for bottom-up planning due to the lack of devolved decision-making power at the village level, and simply adopting a participatory method does not reform the process [3].
75. **The Visual Voices method** is time-consuming, requires participants to be comfortable with various media, and has limited generalizability [138].
76. **The Wild Cards method** was challenging for many participants, who found the wild cards abstract or difficult to grasp [73].
77. **The Youth ReACT (Research Actualizing Critical Thought) data analysis method** faced time constraints that necessitated some initial data filtering by researchers, which reduced the amount of data youth could analyze themselves, thus conflicting with youth participatory action research principles. This method also requires strong facilitation skills, attention to the subtleties of conversations, and patience due to adolescent group dynamics [139].
78. **The Zaltman Metaphor Elicitation Technique (ZMET**) was considered too onerous for participants due to its lengthy two-hour interview process and its need for participants to locate 12 or more images, which some found difficult, leading to duplicate selections. Many participants preferred gathering images overtaking their photos due to ease and time constraints. While participants found the picture selection enjoyable, the reduced number of images impacted the sorting task, which requires categorizing and labeling similar images. Additionally, the small sample size limits the generalizability of the results [140].

References

1. Smetschka B, Gaube V. Co-creating formalized models: Participatory modelling as method and process in transdisciplinary research and its impact potentials. Environ Sci Policy. 2020;103:41–9.

2. Voinov A, Jenni K, Gray S, Kolagani N, Glynn PD, Bommel P, et al. Tools and methods in participatory modeling: Selecting the right tool for the job. Environ Model Softw. 2018;109:232–55.

3. Evans K, Jong W de, Cronkleton P, Nghi TH. Participatory Methods for Planning the Future in Forest Communities. Soc Nat Resour. 2010;23:604–19.

4. Green C. Four Methods for Engaging Young Children as Environmental Education Researchers. Int J Emerg Issues Early Child Educ. 2023;5:6.

5. Wang Q. Art-based narrative interviewing as a dual methodological process: A participatory research method and an approach to coaching in secondary education. Int Coach Psychol Rev. 2016;11:39–56.

6. Lightfoot E, McCleary JS, Lum T. Asset mapping as a research tool for community-based participatory research in social work. Soc Work Res. 2014;38:59–64.

7. Zorrilla P, Carmona G, De la Hera Á, Varela-Ortega C, Martínez-Santos P, Bromley J, et al. Evaluation of Bayesian Networks in Participatory Water Resources Management, Upper Guadiana Basin, Spain. Ecol Soc [Internet]. 2010 [cited 2023 Nov 22];15. Available from: https://www.jstor.org/stable/26268156

8. Switzer S, Guta A, de Prinse K, Chan Carusone S, Strike C. Visualizing harm reduction: Methodological and ethical considerations. Soc Sci Med 1982. 2015;133:77–84.

9. Furman E, Singh AK, Miller Z. “A Space Where People Get It”: A Methodological Reflection of Arts-Informed Community-Based Participatory Research With Nonbinary Youth. Int J Qual Methods [Internet]. 2019; Available from: https://journals.sagepub.com/doi/full/10.1177/1609406919858530

10. Keogh F, Carney P, O’Shea E. Innovative methods for involving people with dementia and carers in the policymaking process. Health Expect Int J Public Particip Health Care Health Policy. 2021;24:800–9.

11. BeLue R, Carmack C, Myers KR, Weinreb-Welch L, Lengerich EJ. Systems Thinking Tools as Applied to Community-Based Participatory Research: A Case Study. 2012 [cited 2024 Sep 28];39. Available from: https://journals.sagepub.com/doi/10.1177/1090198111430708

12. Gerritsen S, Harré S, Rees D, Renker-Darby A, Bartos AE, Waterlander WE, et al. Community Group Model Building as a Method for Engaging Participants and Mobilising Action in Public Health. Int J Environ Res Public Health. 2020;17:3457.

13. Leurs R. The role of the state in enabling private sector development: an assessment methodology. Public Adm Dev [Internet]. 2000 [cited 2024 Sep 10]; Available from: https://onlinelibrary.wiley.com/doi/10.1002/1099-162X(200002)20:1%3C43::AID-PAD110%3E3.0.CO;2-R

14. Timotijevic L, Raats MM. Evaluation of two methods of deliberative participation of older people in food-policy development. Health Policy. 2007;82:302–19.

15. Dennerlein SM, Tomberg V, Treasure-Jones T, Theiler D, Lindstaedt S, Ley T. Co-designing tools for workplace learning. Inf Learn Sci. 2020;121:175–205.

16. Fairchild R, McFerran KS. Understanding Children’s Resources in the Context of Family Violence through a Collaborative Songwriting Method. Child Aust. 2018;43:255–66.

17. O’Reilly-de Brún M, de Brún T, O’Donnell CA, Papadakaki M, Saridaki A, Lionis C, et al. Material practices for meaningful engagement: An analysis of participatory learning and action research techniques for data generation and analysis in a health research partnership. Health Expect. 2018;21:159–70.

18. Amsden J, VanWynsberghe R. Community mapping as a research tool with youth. Action Res. 2005;3:357–81.

19. Beyer KMM, Comstock S, Seagren R. Disease maps as context for community mapping: a methodological approach for linking confidential health information with local geographical knowledge for community health research. J Community Health. 2010;35:635–44.

20. Canham SL, Fang ML, Battersby L, Wada M. Understanding the functionality of housing-related support services through mapping methods and dialogue. Eval Program Plann. 2019;72:33–9.

21. Van Loon AF, Lester-Moseley I, Rohse M, Jones P, Day R. Creative practice as a tool to build resilience to natural hazards in the Global South. Geosci Commun. 2020;3:453–74.

22. Felker-Kantor E, Polanco C, Perez M, Donastorg Y, Andrinopoulos K, Kendall C, et al. Participatory geographic mapping and activity space diaries: innovative data collection methods for understanding environmental risk exposures among female sex workers in a low-to middle-income country. Int J Health Geogr. 2021;20:25.

23. Maalim A d. Participatory rural appraisal techniques in disenfranchised communities: a Kenyan case study. Int Nurs Rev. 2006;53:178–88.

24. Woolner P, Clark J, Hall E, Tiplady L, Thomas U, Wall K. Pictures are necessary but not sufficient: Using a range of visual methods to engage users about school design. Learn Environ Res. 2010;13:1–22.

25. Hill V, Croydon A, Greathead S, Kenny L, Yates R, Pellicano E. Research methods for children with multiple needs: Developing techniques to facilitate all children and young people to have 'a voice.' Educ Child Psychol. 2016;33:26–43.

26. Green EP, Warren VR, Broverman S, Ogwang B, Puffer ES. Participatory mapping in low-resource settings: Three novel methods used to engage Kenyan youth and other community members in community-based HIV prevention research. Glob Public Health. 2016;11:583–99.

27. Lambert V, Glacken M, McCarron M. Using a range of methods to access children's voices. J Res Nurs. 2013;18:601–16.

28. Sewell K. Researching sensitive issues: a critical appraisal of 'draw-and-write' as a data collection technique in eliciting children's perceptions. Int J Res Method Educ. 2011;34:175–91.

29. Haijes HA, van Thiel GJMW. Participatory methods in pediatric participatory research: a systematic review. Pediatr Res. 2016;79:676–83.

30. Nomakhwezi Mayaba N, Wood L. Using Drawings and Collages as Data Generation Methods With Children: Definitely Not Child's Play. Int J Qual Methods. 2015;14:1609406915621407.

31. Nedelcu A. Analysing students' drawings of their classroom: A child-friendly research method. Rev Cercet Si Interv Sociala. 2013;42:275–93.

32. McCarthy L, Muthuri JN. Engaging Fringe Stakeholders in Business and Society Research: Applying Visual Participatory Research Methods. Bus Soc. 2018;57:131–73.

33. Barker J, Weller S. “Is it fun?” developing children centred research methods. Int J Sociol Soc Policy. 2003;23:33–58.

34. Sandman H, Levänen J, Savela N. Using Empathic Design as a Tool for Urban Sustainability in Low-Resource Settings. Sustainability. 2018;10.

35. Hartwig RT. Ethnographic Facilitation as a Complementary Methodology for Conducting Applied Communication Scholarship. J Appl Commun Res. 2014;42:60–84.

36. Wright PR, Wakholi PM. Festival as methodology: the African cultural youth arts festival. Mark Vicars and Dr Jon Austin D, editor. Qual Res J. 2015;15:213–27.

37. Pereira VR, Coimbra VCC, Cardoso C de S, Oliveira NA, Vieira ACG, Nobre M de O, et al. Participatory methodologies in research with children: creative and innovative approaches. Rev Gaucha Enferm. 2017;37:e67908.

38. Wagemakers A, Mulderij LS, Verkooijen KT, Groenewoud S, Koelen MA. Care-physical activity initiatives in the neighbourhood: study protocol for mixed-methods research on participation, effective elements, impact, and funding methods. BMC Public Health [Internet]. 2018; Available from: https://bmcpublichealth.biomedcentral.com/articles/10.1186/s12889-018-5715-z

39. Bulmer SM, Barton BA, Liefeld J, Montauti S, Santos S, Richard M, et al. Using CBPR Methods in College Health Research: Exploring Excessive Alcohol Consumption.

40. Kaptani E, Yuval-Davis N. Participatory Theatre as a Research Methodology: Identity, Performance and Social Action among Refugees. Sociol Res Online. 2008;13:1–12.

41. Schaper M-M, Iversen OS, Malinverni L, Pares N. FUBImethod: Strategies to engage children in the co-design of Full-Body interactive experiences. Int J Hum-Comput Stud. 2019;132:52–69.

42. Lahtinen M, Nenonen S, Rasila H, Lehtelä J, Ruohomäki V, Reijula K. Rehabilitation centers in change: participatory methods for managing redesign and renovation. HERD. 2014;7:57–75.

43. Townley G, Kloos B, Wright PA. Understanding the experience of place: Expanding methods to conceptualize and measure community integration of persons with serious mental illness. Health Place. 2009;15:520–31.

44. North N, Sieberhagen S, Leonard A, Bonaconsa C, Coetzee M. Making Children’s Nursing Practices Visible: Using Visual and Participatory Techniques to Describe Family Involvement in the Care of Hospitalized Children in Southern African Settings. Int J Qual Methods. 2019;18:1609406919849324.

45. Redman-MacLaren M, Mills J, Tommbe R. Interpretive focus groups: a participatory method for interpreting and extending secondary analysis of qualitative data. Glob Health Action. 2014;7:25214.

46. Lang A. Seniors managing multiple medications: using mixed methods to view the home care safety lens. BMC Health Serv Res [Internet]. [cited 2024 Sep 10]; Available from: https://bmchealthservres.biomedcentral.com/articles/10.1186/s12913-015-1193-5

47. Chavarria EA, Christy SM, Simmons VN, Vadaparampil ST, Gwede CK, Meade CD. Learner Verification: A Methodology to Create Suitable Education Materials |. HLRP Health Lit Res Pract. 2021;5:e49–59.

48. Pedell S, Keirnan A, Priday G, Miller T, Mendoza A, Lopez-Lorca A, et al. Methods for Supporting Older Users in Communicating Their Emotions at Different Phases of a Living Lab Project. Technol Innov Manag Rev. 2017;7:7–19.

49. Segal-Engelchin D, Huss E, Massry N. Arts-Based Methodology for Knowledge Co-Production in Social Work. Br J Soc Work. 2020;50:1277–94.

50. Blodgett AT, Coholic DA, Schinke RJ, McGannon KR, Peltier D, Pheasant C. Moving beyond words: exploring the use of an arts-based method in Aboriginal community sport research. Qual Res Sport Exerc Health. 2013;5:312–31.

51. Pereira L, Hichert T, Hamann M, Preiser R, Biggs R. Using futures methods to create transformative spaces: visions of a good Anthropocene in southern Africa. Ecol Soc [Internet]. 2018 [cited 2024 Sep 28];23. Available from: https://www.ecologyandsociety.org/vol23/iss1/art19/

52. Calba C, Ponsich A, Nam S, Collineau L, Min S, Thonnat J, et al. Development of a participatory tool for the evaluation of Village Animal Health Workers in Cambodia. Acta Trop. 2014;134:17–28.

53. Burgess H, Jongbloed K, Vorobyova A, Grieve S, Lyndon S, Wesseling T, et al. The “Sticky Notes” Method: Adapting Interpretive Description Methodology for Team-Based Qualitative Analysis in Community-Based Participatory Research. Qual Health Res. 2021;31:1335–44.

54. Burgess-Allen J, Owen-Smith V. Using mind mapping techniques for rapid qualitative data analysis in public participation processes.

55. Ospina-Pinillos L, Davenport TA, Ricci CS, Milton AC, Scott EM, Hickie IB. Developing a Mental Health eClinic to Improve Access to and Quality of Mental Health Care for Young People: Using Participatory Design as Research Methodologies. J Med Internet Res. 2018;20:e9716.

56. Revez A, Dunphy N, Harris C, Mullally G, Lennon B, Gaffney C. Beyond Forecasting: Using a Modified Delphi Method to Build Upon Participatory Action Research in Developing Principles for a Just and Inclusive Energy Transition. Int J Qual Methods. 2020;19:160940692090321.

57. Kensing F, Sigurdardottir H, Stoop A. MUST--a participatory method for designing sustainable health IT. Stud Health Technol Inform. 2007;129:1204–8.

58. Litovuo L, Karisalmi N, Aarikka-Stenroos L, Kaipio J. Comparing Three Methods to Capture Multidimensional Service Experience in Children’s Health Care: Video Diaries, Narratives, and Semistructured Interviews. Int J Qual Methods. 2019;18:1609406919835112.

59. Hemming PJ. Mixing Qualitative Research Methods in Children’s Geographies. Area. 2008;40:152–62.

60. Mullings L, Wali A, McLean D, Mitchell J, Prince S, Thomas D, et al. Qualitative methodologies and community participation in examining reproductive experiences: the Harlem Birth Right Project. Matern Child Health J. 2001;5:85–93.

61. O'Hara L, Higgins K. Participant Photography as a Research Tool: Ethical Issues and Practical Implementation. Sociol Methods Res. 2019;48:369–99.

62. Lahtinen M, Nenonen S, Rasila H, Lehtelä J, Ruohomäki V, Reijula K. Rehabilitation Centers in Change: Participatory Methods for Managing Redesign and Renovation. HERD Health Environ Res Des J. 2014;7:57–75.

63. Ekirapa-Kiracho E, Ghosh U, Brahmachari R, Paina L. Engaging stakeholders: lessons from the use of participatory tools for improving maternal and child care health services. Health Res Policy Syst. 2017;15:106.

64. Forrester J, Cook B, Bracken L, Cinderby S, Donaldson A. Combining participatory mapping with Q-methodology to map stakeholder perceptions of complex environmental problems. Appl Geogr. 2015;56:199–208.

65. Best P, Badham J, Corepal R, O'Neill RF, Tully MA, Kee F, et al. Network methods to support user involvement in qualitative data analyses: an introduction to Participatory Theme Elicitation. Trials. 2017;18:559.

66. Treves A, Andriamampianina L, Didier K, Gibson J, Plumptre A, Wilkie D, et al. A Simple, Cost-Effective Method for Involving Stakeholders in Spatial Assessments of Threats to Biodiversity. Hum Dimens Wildl. 2006;11:43–54.

67. Tremblay C. Towards inclusive waste management: participatory video as a communication tool. Proc Inst Civ Eng - Waste Resour Manag. 2013;166:177–86.

68. D'Amico M, Denov M, Khan F, Linds W, Akesson B. Research as intervention? Exploring the health and well-being of children and youth facing global adversity through participatory visual methods. Glob Public Health. 2016;11:528–45.

69. Lomax H, Fink J, Singh N, High C. The politics of performance: methodological challenges of researching children's experiences of childhood through the lens of participatory video. Int J Soc Res Methodol. 2011;14:231–43.

70. Wood L, Olivier T. Video production as a tool for raising educator awareness about collaborative teacher–parent partnerships. Educ Res. 2011;53:399–414.

71. Gerodimos R. Youth and the City: Reflective Photography as a Tool of Urban Voice. J Media Lit Educ. 2018;10:82–103.

72. Parker M, Wallerstein N, Duran B, Magarati M, Burgess E, Sanchez-Youngman S, et al. Engage for Equity: Development of community-based participatory research tools. 2020;47. Available from: https://journals.sagepub.com/doi/10.1177/1090198120921188

73. O'Brien N, Heaven B, Teal G, Evans EH, Cleland C, Moffatt S, et al. Integrating Evidence From Systematic Reviews, Qualitative Research, and Expert Knowledge Using Co-Design Techniques to Develop a Web-Based Intervention for People in the Retirement Transition. J Med Internet Res. 2016;18:e5790.

74. Trischler J, Scott DR. Designing Public Services: The usefulness of three service design methods for identifying user experiences. Public Manag Rev. 2016;18:718–39.

75. Lorenz LS, Kolb B. Involving the public through participatory visual research methods. Health Expect. 2009;12:262–74.

76. Najib Balbale S, Schwingel A, Chodzko-Zajko W, Huhman M. Visual and Participatory Research Methods for the Development of Health Messages for Underserved Populations. Health Commun. 2014;29:728–40.

77. Due C, Riggs DW, Augoustinos M. Research with Children of Migrant and Refugee Backgrounds: A Review of Child-Centered Research Methods. Child Indic Res. 2014;7:209–27.

78. Sebastião E, Gálvez PAE, Bobitt J, Adamson BC, Schwingel A. Visual and participatory research techniques: photo-elicitation and its potential to better inform public health about physical activity and eating behavior in underserved populations. J Public Health. 2016;24:3–7.

79. Kort HSM, Steunenberg B, van Hoof J. Methods for Involving People Living with Dementia and Their Informal Carers as Co-Developers of Technological Solutions. Dement Geriatr Cogn Disord. 2019;47:149–56.

80. Backman C, Stacey D, Crick M, Cho-Young D, Marck PB. Use of participatory visual narrative methods to explore older adults' experiences of managing multiple chronic conditions during care transitions.

81. Molloy JK. Photovoice as a Tool for Social Justice Workers. J Progress Hum Serv. 2007;18:39–55.

82. Walker A, Oomen-Early J. Do You See What I See? Using Photovoice to Teach Health Education Students the Value of Using Community-Based Participatory Methods. J Health Educ Teach Tech. 2014;1:41–52.

83. Valiquette-Tessier S-C, Vandette M-P, Gosselin J. In Her Own Eyes: Photovoice as an Innovative Methodology to Reach Disadvantaged Single Mothers. Can J Commun Ment Health. 2015;34:1–16.

84. Rania N, Coppola I, Pinna L. Adapting Qualitative Methods during the COVID-19 Era: Factors to Consider for Successful Use of Online Photovoice. Qual Rep. 2021;26:2711–29.

85. Saunders G, Dillard L, Frederick M, Silverman S. Examining the Utility of Photovoice as an Audiological Counseling Tool. J Am Acad Audiol. 2019;30.

86. Sutton-Brown CA. Photovoice: A Methodological Guide.

87. Lal S, Jarus T, Suto MJ. A Scoping Review of the Photovoice Method: Implications for Occupational Therapy Research. Can J Occup Ther. 2012;79:181–90.

88. Drawson AS, Toombs E, Mushquash CJ. Indigenous Research Methods: A Systematic Review. Int Indig Policy J [Internet]. 2017 [cited 2024 Sep 10];8. Available from: https://ojs.lib.uwo.ca/index.php/iipj/article/view/7515

89. Teti M, Murray C, Johnson L, Binson D. Photovoice as a Community-Based Participatory Research Method among Women Living with HIV/AIDS: Ethical Opportunities and Challenges. J Empir Res Hum Res Ethics. 2012;7:34–43.

90. Campbell RB, Larsen M, DiGiandomenico A, Davidson MA, Booth GL, Hwang SW, et al. The challenges of managing diabetes while homeless: a qualitative study using photovoice methodology. CMAJ. 2021;193:E1034–41.

91. Madrigal DS, Salvatore A, Casillas G, Casillas C, Vera I, Eskenazi B, et al. Health in My Community: Conducting and Evaluating PhotoVoice as a Tool to Promote Environmental Health and Leadership Among Latino/a Youth. Prog Community Health Partnersh Res Educ Action. 2014;8:317–29.

92. MacFarlane EK, Shakya R, Berry HL, Kohrt BA. Implications of participatory methods to address mental health needs associated with climate change: 'photovoice' in Nepal. BJPsych Int. 2015;12:33–5.

93. Houle J, Coulombe S, Radziszewski S, Leloup X, Saïas T, Torres J, et al. An intervention strategy for improving residential environment and positive mental health among public housing tenants: rationale, design and methods of Flash on my neighborhood! BMC Public Health. 2017;17:737.

94. Aw S, Koh GC, Oh YJ, Wong ML, Vrijhoef HJ, Harding SC, et al. Interacting with place and mapping community needs to context: Comparing and triangulating multiple geospatial-qualitative methods using the Focus–Expand–Compare approach. Methodol Innov. 2021;14:205979912098777.

95. Nykiforuk CIJ, Vallianatos H, Nieuwendyk LM. Photovoice as a Method for Revealing Community Perceptions of the Built and Social Environment. Int J Qual Methods. 2011;10:103–24.

96. Kramer L, Schwartz P, Cheadle A, Rauzon S. Using Photovoice as a Participatory Evaluation Tool in Kaiser Permanente's Community Health Initiative. Health Promot Pract. 2013;14:686–94.

97. Downey LH, Ireson CL, Scutchfield FD. The Use of Photovoice as a Method of Facilitating Deliberation. Health Promot Pract. 2009;10:419–27.

98. Pauwels L. 'Participatory' visual research revisited: A critical-constructive assessment of epistemological, methodological and social activist tenets. Ethnography. 2015;16:95–117.

99. Livingood WC, Monticalvo D, Bernhardt JM, Wells KT, Harris T, Kee K, et al. Engaging Adolescents Through Participatory and Qualitative Research Methods to Develop a Digital Communication Intervention to Reduce Adolescent Obesity.

100. Golden T. Reframing Photovoice: Building on the Method to Develop More Equitable and Responsive Research Practices. Qual Health Res. 2020;30:960–72.

101. Baker TA, Wang CC. Photovoice: Use of a Participatory Action Research Method to Explore the Chronic Pain Experience in Older Adults. Qual Health Res. 2006;16:1405–13.

102. Johnson LR, Drescher CF, Assenga SH, Marsh RJ. Assessing Assets Among Street-Connected Youth: New Angles With Participatory Methods in Tanzania. J Adolesc Res. 2019;34:619–51.

103. Bisung E, Elliott SJ, Abudho B, Karanja DM, Schuster-Wallace CJ. Using Photovoice as a Community Based Participatory Research Tool for Changing Water, Sanitation, and Hygiene Behaviours in Usoma, Kenya.

104. Marshalsey L, Sclater M. Arts-Based Educational Research: The Challenges of Social Media and Video-Based Research Methods in Communication Design Education.

105. Maclean K, Woodward E. Photovoice Evaluated: An Appropriate Visual Methodology for Aboriginal Water Resource Research. Geogr Res. 2013;51:94–105.

106. Witkowski K, Matiz Reyes A, Padilla M. Teaching diversity in public participation through participatory research: A case study of the PhotoVoice methodology. J Public Aff Educ. 2021;27:218–37.

107. Fay F. The impact of the school space on research methodology, child participation and safety: views from children in Zanzibar. Child Geogr. 2018;16:405–17.

108. Umurungi J-P, Mitchell C, Gervais M, Ubalijoro E, Kabarenzi V. Photovoice as a Methodological Tool to Address HIV and AIDS and Gender Violence amongst Girls on the Street in Rwanda. J Psychol Afr. 2008;18:413–9.

109. Barry J, Higgins A. PhotoVoice: An Ideal Methodology for Use within Recovery-Oriented Mental Health Research. Issues Ment Health Nurs. 2021;42:676–81.

110. Capous-Desyllas M, Forro VA. Tensions, Challenges, and Lessons Learned: Methodological Reflections From Two Photovoice Projects With Sex Workers. J Community Pract. 2014;22:150–75.

111. Sitter KC. Taking a Closer Look at Photovoice as a Participatory Action Research Method. J Progress Hum Serv. 2017;28:36–48.

112. Peabody CG. Using Photovoice as a Tool to Engage Social Work Students in Social Justice. J Teach Soc Work. 2013;33:251–65.

113. Russinova Z, Mizock L, Bloch P. Photovoice as a tool to understand the experience of stigma among individuals with serious mental illnesses. Stigma Health. 2018;3:171–85.

114. Novek S, Morris-Oswald T, Menec V. Using photovoice with older adults: some methodological strengths and issues. Ageing Soc. 2012;32:451–70.

115. Ronzi S, Pope D, Orton L, Bruce N. Using photovoice methods to explore older people's perceptions of respect and social inclusion in cities: Opportunities, challenges and solutions. SSM - Popul Health. 2016;2:732–45.

116. Krutt H, Dyer L, Arora A, Rollman J, Jozkowski AC. PhotoVoice is a feasible method of program evaluation at a center serving adults with autism. Eval Program Plann. 2018;68:74–80.

117. Doucet M, Pratt H, Dzhenganin M, Read J. Nothing About Us Without Us: Using Participatory Action Research (PAR) and arts-based methods as empowerment and social justice tools in doing research with youth "aging out" of care. Child Abuse Negl. 2022;130:105358.

118. Participatory Methodologies With Adolescents: A Research Approach Used to Explore Structural Factors Affecting Alcohol Use and Related Unsafe Sex in Tanzania | Journal of Prevention [Internet]. [cited 2024 Sep 29]. Available from: https://link.springer.com/article/10.1007/s10935-020-00586-0

119. Foster-Fishman P, Nowell B, Deacon Z, Nievar MA, McCann P. Using methods that matter: the impact of reflection, dialogue, and voice. Am J Community Psychol. 2005;36:275–91.

120. Hemy AD, Meshulam A. 'Is that okay, teacher?' The camera as a tool to challenge power relations in a participatory action research classroom. Qual Res. 2021;21:750–67.

121. Valerio MA, Rodriguez N, Winkler P, Lopez J, Dennison M, Liang Y, et al. Comparing two sampling methods to engage hard-to-reach communities in research priority setting. BMC Med Res Methodol [Internet]. 16. Available from: https://bmcmedresmethodol.biomedcentral.com/articles/10.1186/s12874-016-0242-z

122. Cross-Sudworth F, Williams A, Herron-Marx S. Maternity services in multi-cultural Britain: Using Q methodology to explore the views of first- and second-generation women of Pakistani origin. Midwifery. 2011;27:458–68.

123. Forsyth A, Slotterback CS, Krizek KJ. Health impact assessment in planning: Development of the design for health HIA tools. Environ Impact Assess Rev. 2010;30:42–51.

124. Calvo M. "Reflective drawing": Drawing as a tool for reflection in design research. 2016.

125. Vieira Pak M, Castillo Brieva D. Designing and implementing a Role-Playing Game: A tool to explain factors, decision making and landscape transformation.

126. Bhanjee T, Mugabe M, Mawoneke S. Measuring HIV and AIDS Community Competence Using SAT's Competence Tool (SATCOMP): A Case Study of AIDS Among Us in Zimbabwe. Can J Public Health. 2008;99:S27–34.

127. Cottafava D, Corazza L. Co-design of a stakeholders' ecosystem: an assessment methodology by linking social network analysis, stakeholder theory and participatory mapping.

128. Yates KL, Schoeman DS. Spatial Access Priority Mapping (SAPM) with Fishers: A Quantitative GIS Method for Participatory Planning. PLOS ONE. 2013;8:e68424.

129. Rockloff SF, Lockie S. Participatory tools for coastal zone management: Use of stakeholder analysis and social mapping in Australia. J Coast Conserv. 2004;10:81–92.

130. Blanc S, Lingua F, Bioglio L, Pensa RG, Brun F, Mosso A. Implementing Participatory Processes in Forestry Training Using Social Network Analysis Techniques. Forests. 2018;9:463.

131. Reed MS, Graves A, Dandy N, Posthumus H, Hubacek K, Morris J, et al. Who's in and why? A typology of stakeholder analysis methods for natural resource management. J Environ Manage. 2009;90:1933–49.

132. Bird S, Wiles JL, Okalik L, Kilabuk J, Egeland GM. Methodological consideration of story telling in qualitative research involving Indigenous Peoples. Glob Health Promot. 2009;16:16–26.

133. Fraser SL. What stories to tell? A trilogy of methods used for knowledge exchange in a community-based participatory research project. Action Res. 2018;16:207–22.

134. Cerreta M, De Toro P. Integrated spatial assessment for a creative decision-making process: a combined methodological approach to strategic environmental assessment.

135. Kohfeldt D, Langhout R. The Five Whys Method: A Tool for Developing Problem Definitions in Collaboration with Children. J Community Appl Soc Psychol. 2012;22:316–29.

136. Berger-González M, Stauffacher M, Zinsstag J, Edwards P, Krütli P. Transdisciplinary Research on Cancer-Healing Systems Between Biomedicine and the Maya of Guatemala: A Tool for Reciprocal Reflexivity in a Multi-Epistemological Setting.

137. Pollock A, Campbell P, Baer G, Choo PL, Morris J, Forster A. User involvement in a Cochrane systematic review: using structured methods to enhance the clinical relevance, usefulness and usability of a systematic review update. Syst Rev. 2015;4:55.

138. Yonas MA, Burke JG, Miller E. Visual Voices: A Participatory Method for Engaging Adolescents in Research and Knowledge Transfer. Clin Transl Sci. 2013;6:72–7.

139. Foster-Fishman PG, Law KM, Lichty LF, Aoun C. Youth ReACT for Social Change: A Method for Youth Participatory Action Research. Am J Community Psychol. 2010;46:67–83.

140. Dodds S, Bulmer S, Murphy A. Incorporating visual methods in longitudinal transformative service research. J Serv Theory Pract. 2018;28:434–57.

141. Timotijevic L, Raats MM. Evaluation of two methods of deliberative participation of older people in food-policy development. Health Policy Amst Neth. 2007;82:302–19.

142. Haque N, Rosas S. Concept Mapping of Photovoices: Sequencing and Integrating Methods to Understand Immigrants' Perceptions of Neighborhood Influences on Health. Fam Community Health. 2010;33:193.

143. Thompson JR, Burke JG. Increasing Community Participation in Public Health Research: Applications for Concept Mapping Methodology. Prog Community Health Partnersh Res Educ Action. 2020;14:243–50.

144. Ahmad F, Norman C, O'Campo P. What is needed to implement a computer-assisted health risk assessment tool? An exploratory concept mapping study. BMC Med Inform Decis Mak. 2012;12:149.

145. Burke JG, O'Campo P, Peak GL, Gielen AC, McDonnell KA, Trochim WMK. An introduction to concept mapping as a participatory public health research method. Qual Health Res. 2005;15:1392–410.

146. Vaughn LM, Jones JR, Booth E, Burke JG. Concept mapping methodology and community-engaged research: A perfect pairing. Eval Program Plann. 2017;60:229–37.

147. Windsor LC. Using Concept Mapping in Community-Based Participatory Research: A Mixed Methods Approach. J Mix Methods Res. 2013;7:274–93.

148. Ravarotto L, Crovato S, Mantovani C, D'Este F, Pinto A, Mascarello G. Reducing microbiological risk in the kitchen: piloting consensus conference methodology as a communication strategy. J Risk Res. 2016;19:934–50.

149. Scott-Bottoms S, Roe M. Who is a hydrocitizen? The use of dialogic arts methods as a research tool with water professionals in West Yorkshire, UK. Local Environ. 2020;25:273–89.

150. Caretta MA, Vacchelli E. Re-Thinking the Boundaries of the Focus Group: A Reflexive Analysis on the Use and Legitimacy of Group Methodologies in Qualitative Research.

151. Barnidge E, Baker EA, Motton F, Rose F, Fitzgerald T. A participatory method to identify root determinants of health: the heart of the matter.

152. Moletsane R, de Lange N, Mitchell C, Stuart J, Buthelezi T, Taylor M. Photo-voice as a tool for analysis and activism in response to HIV and AIDS stigmatisation in a rural Kwazulu-Natal school.

153. Campbell DJT, Davidson MA. The challenges of managing diabetes while homeless: a qualitative study using photovoice methodology: CMAJ.

154. López EDS, Eng E, Randall-David E, Robinson N. Quality-of-Life Concerns of African American Breast Cancer Survivors Within Rural North Carolina: Blending the Techniques of Photovoice and Grounded Theory. Qual Health Res. 2005;15:99–115.

155. Nyström A-G, Mustonen M, Yrjölä S. Co-Creating User Stories: A Tool for Making Sense of Business Opportunities.
