# Supplementary material for: Co-creation methods for public health research — characteristics, benefits, and challenges: a Health CASCADE scoping review
Source: BMC Med Res Methodol. 2025 Mar 6;25:60. doi: 10.1186/s12874-025-02514-4 (PMC11884017; doi:10.1186/s12874-025-02514-4)
Supplement: Supplementary file 8 — Additional file 8. [file 12874_2025_2514_MOESM8_ESM.pdf]

Additional File 8. Methods per Theme and Sub-Theme

Method Benefits

Data was extracted per method regarding the benefits of each method: 139 studies reported the benefits of 106 different methods, and the following is a summary of the findings in grouped 9 themes and 27 sub-themes.

Theme 1: Collaboration and Participation

Collaboration and participation are central to 34 different co-creation methods, fostering collective learning, peer engagement, and problem-solving. Table 1 contains the sub-theme name, description, and associated methods.

Table 1. Theme 1 - Collaboration and Participation's three sub-themes and associated methods

Sub-theme	Method Name [source study]	Key Benefits
Collaboration	20 Methods: Agent-based Models [1,2]; Alternative Scenarios [3]; Art Making [4]; Asset Mapping [5]; Concept Mapping [6–12]; Draw and Write Technique [13–15]; Ethnographic Facilitation [16]; Five Field Map [17]; Future Workshop [18]; Metaplan [19]; Modified Delphi [20]; Participatory Video [21–23]; Photo-elicitation [24–29]; Photo Walkabouts [30,31]; Photovoice [22,26,32–71]; Playback Theatre [32], Role-Playing Games [72]; Transdisciplinarity [73]; and The Visioning Tool [74].	Collective learning, peer engagement, rapport-building, collaborative problem-solving, storytelling, fostering shared understanding, and long-term partnerships.
Meaningful Participation	15 Methods: Alternative Scenarios [3]; Draw and Write Technique [13–15]; Co-design by Appropriation of Affordances [75]; Community charts [76]; Community Mapping [77–79]; Concept Mapping [6–12]; Participatory Geographic Mapping [80]; Participatory Impact Pathways Analysis [81]; Ethnographic Facilitation [16]; Participatory Visual Methodology [82]; Participatory/Reflective Photography [83]; like Reflective Drawing [84]; Sociograms [25]; Snowball Sampling [85]; and Stick-a-star Quiz [13].	Active engagement, participant empowerment, continuous interaction, community involvement, meaningful engagement, particularly among children and non-traditional participants.
Socializing and Connection	7 Methods: Art Making [4]; Body Mapping [86]; Role-playing [4]; Empathic Design [87]; Participatory/Reflective Photography	Facilitates social and deeper connection through shared experiences, enhances community ties, builds

	[83]; Five Field Map [17]; and Participant Observation [88,89].	relationships through artistic expression or peer engagement, especially among youth. Promotes meaningful social interactions.

Theme 2: Empowerment and Agency

Empowerment and agency are central to 65 co-creation methods, promoting democratic participation, decentralized decision-making, and reducing power imbalances.

Table 2. Theme 2 - Empowerment and Agency's five sub-themes and associated methods

Sub-theme	Method Name [source study]	Key Benefits
Decision-making and Democracy	7 Methods: Agent-based Modelling [1,2]; Alternative Scenarios [3]; Community Charts [76]; Direct Ranking [76]; Diamond Ranking [24,90]; Decision Tree Analyses [2]; Strategic Environmental Assessment [91].	Promotes democratic participation, and decentralized decision-making, reduces power imbalances, links decisions to outcomes, and integrates environmental values.
Heard and supported	19 Methods: A Blended Approach of Photovoice and Photo-elicitation [86]; Body Mapping [86]; The Carer's Assembly [92]; Citizen's Jury [93]; Citizen's Workshop [93]; Collaborative Songwriting [77]; Community Charts [76]; Creative Practice [94]; Direct Ranking [76]; Forum Theatre [32]; Informal Interviews [13]; Interpretive Focus Groups [95]; Illustrative Arts-based Methodology [96]; Photovoice [22,26,32–71]; Stakeholder Analysis [97–99]; Rapid HIA [100]; Spatial Access Priority Mapping [101]; Stick-a-Star Quiz [13]; and Learner Verification and Revision [102].	Supportive environments, participant voice amplification, meaningful engagement, self-expression, inclusive dialogue, conflict understanding, flexibility in expression, and structural support for collaboration.
Sense of Ownership	5 Methods: Co-design by Appropriation of Affordances [75]; Community charts [76]; Direct ranking [76]; Drawings [103–105]; The Mānoa mash-up [106]; and Participatory mapping [107,108].	Participant ownership, process control, belief in co-created outcomes, encouragement of personal framework development, and enhanced investment in results.
Empowerment	15 Methods: Community charts [76]; Direct ranking [76]; Ethnographic Facilitation [16]; The festival [109]; Illustrative arts-based methodology [96]; Mandala drawing [110]; Participatory geographic mapping [80]; Participatory Video [21–23]; Participatory/reflective photography [83]; Photovoice [22,26,32–71]; PowerView [111]; The SAT's Competence Tool [112]; Stick-a-Star Quiz [13]; Transdisciplinarity [73]; and The Visioning Tool [74].	Empowerment promotion, addressing power dynamics and imbalances, fostering equal partnerships, enhancing community leadership, encouraging community-led engagement, inverting traditional hierarchies, and valuing participant knowledge and experience.

Inclusive and accessible	38 Methods: A blended approach of photovoice and photo-elicitation [86]; Causal Loop Diagrams [2,113,114]; Checklist [115]; Citizen's Jury [93]; Citizens' Workshop [93]; Transdisciplinarity [73]; Concept mapping [6–12]; Direct ranking [76]; Dot Map Focus Group [116]; The draw and write technique [13–15]; Draw and Write/talk method [117]; Drawings [103–105]; Focus group [118,119]; Forum theatre [32]; Learner Verification and Revision [102]; Illustrative arts-based methodology [96]; Metaplan method [19]; Mind mapping [120,121]; The Modified Delphi [20]; Narrative interviews [122]; Participant observation [88,89]; Participant photography [123]; Participatory Design Generator Cards [124]; Participatory geographic mapping [80]; Participatory mapping [107,108]; Participatory Theme Elicitation [125]; Participatory Video [21–23]; Photo-elicitation [24–29]; Photovoice [22,26,32–71]; Rapid HIA [100]; Role-Playing Games [72]; Snowball sampling [85]; Structured surveys [2]; Transdisciplinarity [73]; User-driven systematic reviews [126]; Visual Voices method [127]; and Alternative Scenarios [3].	Inclusivity promotion, accessibility for non-technical audiences, engagement of marginalized groups, language barrier transcendence, cultural sensitivity adaptation, safe engagement spaces, broad participation facilitation, and empowerment of diverse perspectives.

Theme 3: Innovation and Creativity

Innovation and creativity are central to 91 co-creation methods, fostering creativity connection, stimulating curiosity, and facilitating storytelling. Table 4 contains the sub-theme name, description, and associated methods.

Table 3. Innovation and Creativity's three sub-themes and associated methods

Sub-theme	Method Name [source study]	Key Benefits
Creativity and fun	20 Methods: Alternative scenarios [3]; Art making [4]; A blended approach of photovoice and photo-elicitation [86]; Body mapping [86]; Collaborative songwriting [77]; A Dot map focus group [116]; Draw and Write Technique [13–15]; Five Field Map [17]; Forum theatre [32]; Mandala drawing [110]; The Mānoa mash-up [106]; Participant photography [123]; Participatory Design Generator Cards [124]; Participatory Video [21–23]; Participatory/reflective photography [83]; Playback Theatre [32]; Role-playing [4]; Sociogram [25]; and	Creativity connection, curiosity stimulation, storytelling facilitation, interactive and enjoyable experiences, collective engagement, dynamic environment creation, and youth engagement enhancement.

	Zaltman Metaphor Elicitation Technique [128].	
Innovation or new ideas	<u>34 Methods</u>: Causal Loop Diagrams [2,113,114]; Checklist [115]; Co-design by Appropriation of Affordances [75]; Community charts [76]; Community mapping [77–79]; Daily Activity Space Travel Diary [80]; Diamond ranking [24,90]; Direct ranking [76]; Focus Group [118,119]; Fuzzy Cognitive Mapping [2]; Graphs Over Time[113]; Living Lab [129]; Illustrative Arts-based Methodology [96]; Mockups of webpages [130]; MUST method [131]; Participatory Design Generator Cards [124]; Participatory geographic mapping [80]; Participatory Video [21–23]; Participatory visual methodology [82]; Participatory/reflective photography [83]; Partnership Data Report for Reflection [74]; Pathways [3]; Persona Building [132]; Photo-elicitation [24–29]; Photovoice [22,26,32–71]; Playback Theatre [32]; Rapid HIA [100]; Threshold Analysis [100]; Reflective Drawing [84]; SAT's Competence Tool [112]; Social Network Analysis [2,133]; Stakeholder analysis [97–99]; Storytelling [134,135]; Five Whys Method [136]; and Visioning Tool [74].	Deeper thinking, expanded focus, broader intervention options identification, innovative practice development, non-static thinking promotion, emotional dynamics exploration, hidden tension uncovering, problem-solving insights, brainstorming support, and coherent project vision development.
Knowledge Integration	<u>Methods:</u> Agent-Based Models [1,2]; Art making [4]; Art-based narrative interview [137]; Bayesian Networks [2,138]; Body mapping [86]; Building a Model [4]; Citizen's Jury [93]; Citizens' Workshop [93]; Cognitive Mapping [2,113]; Concept Mapping [6–12]; Daily Work Schedules [139]; A Dot Map Focus Group [116]; Draw and Write Technique [13–15]; Empathic Design [87]; Ethnographic Facilitation [16]; The festival [109]; Fuzzy cognitive mapping [2]; Mānoa mash-up [106]; Metaplan method [19]; Mind mapping [120,121]; Personas [140]; Photo-elicitation [24–29]; Photovoice [22,26,32–71]; Playback Theatre [32]; Rapid HIA [100]; Spatial access priority mapping [101]; Stakeholder analysis [97–99]; Stick-a-Star Quiz [13]; Strategic Environmental Assessment [91]; Structured surveys [2]; Threshold Analysis [100]; Transdisciplinarity [73]; User-driven systematic reviews [122]; Video Diary Method [122]; Visual Voices method [127]; and Zaltman Metaphor Elicitation Technique [128].	Knowledge integration, stakeholder engagement, diverse perspective representation, enhanced credibility, emotional exploration, community dynamics understanding, practical knowledge conveyance, social and cultural sustainability addressing, and collective sharing.

Theme 4: Well-being and Satisfaction

Well-being and satisfaction are key benefits of 37 co-creation methods, offering therapeutic effects and reducing stigmatization while providing a relaxing, anxiety-reducing environment. Table 5 contains the sub-theme name, description, and associated methods.

Table 4. Well-being and Satisfaction's four sub-themes and associated methods

Sub-theme	Method Name [source study]	Key Benefits
Improved well-being	8 Methods: A blended approach of photovoice and photo-elicitation [86]; Collaborative songwriting [77]; Draw and Write Technique [13–15]; Draw and Write/talk method [117]; Forum theatre [32]; Interpretive focus group [95]; Mandala Drawing [110]; and Participatory Visual Methodology [82].	Reduces stigmatization, promotes therapeutic effects, and provides a relaxing, anxiety-reducing environment. Offers socioemotional benefits, emotional release, and a more comfortable setting for participants, alleviating tension and apprehension. Facilitates the exploration of sensitive topics in a non-threatening manner.
Satisfied	4 Methods: Citizen's Jury [93]; Citizens' Workshop [93]; Visioning Tool [74]; and Zaltman Metaphor Elicitation Technique [128].	High subjective satisfaction with the process, with participants expressing a likelihood of using it again. Participants found their involvement meaningful and appreciated the opportunity to contribute.
Trust	5 Methods: Citizen's Jury [93]; Citizens' Workshop [93]; Co-design by Appropriation of Affordances[75]; Participant observation [88,89]; and Participatory visual methodology [82].	Participants trusted the organizer, considering them trustworthy, which fostered engagement. The long-term approach built trust, allowing for the exploration of sensitive topics.
Motivation and Inspiration	14 Methods: Co-design by Appropriation of Affordances[75]; Community charts [76]; Direct ranking [76]; Community mapping [77–79]; Concept mapping [6–12]; Daily Activity Space Travel Diary [80]; Diamond ranking [24,90]; The future workshop [18]; Participatory Design Generator Cards [124]; Participatory Threats Assessment [141]; SAT's Competence Tool [112]; Spatial access priority mapping [101]; Stick-a-Star Quiz [13]; and Storyboards and animations [129].	Motivation enhancement, sustained engagement, solution-seeking stimulation, strong commitment fostering, incentive-based participation, enthusiasm generation, threat identification eagerness, knowledge sharing encouragement, and active engagement promotion.

Theme 5: Communication and Transparency

Communication and Transparency are key benefits of 39 co-creation methods in this theme, enhancing stakeholder communication through experience sharing, rapport building, and non-verbal expression. Table 6 contains the sub-theme name, description, and associated methods.

Table 5. Communication and Transparency's two sub-themes and associated methods

Sub-theme	Method Name [source study]	Key Benefits
Enhanced communication	31 Methods: Agent-Based Models [1,2]; Alternative scenarios [3]; Art making [4]; Art-based Narrative Interview [142]; Bayesian Networks [2,138]; Causal Loop Diagrams [2,113,114]; Concept mapping [6–12]; Cultural consensus [2]; Decision tree analyses [2]; Diamond ranking [24,90]; Draw and Write Technique [13–15]; Focus Group [118,119]; Future Workshop [18]; Geographic Information Systems [2,79,107]; Graphic Facilitation [25]; Informal Interviews [13]; Kitchen Table Talk [30]; Learner Verification and Revision [102]; Mandala drawing [110]; Participatory Design Generator Cards [124]; Participatory Video [21–23]; Participatory/reflective photography [83]; Personas [140]; Photo-elicitation [24–29]; Photovoice [22,26,32–71]; Prototyping [132]; SAT's Competence Tool [112]; Sociogram [25]; Stick-a-Star Quiz [13]; Threshold Analysis [100]; and Visual Voices method [127].	Communication enhancement, experience sharing, rapport building, non-verbal expression facilitation, key issue exploration, focused dialogue stimulation, idea clarification, visual communication support, participatory communication promotion, controversy addressing, natural dialogue fostering, and culturally significant communication capturing.
Transparency	9 Methods: Agent-Based Models [1,2]; Alternative scenarios [3]; Citizen's Jury [93]; Cognitive Mapping [2,113]; Community Mapping [77–79]; Cultural consensus [2]; Decision Tree Analyses [2]; Geographic Information Systems [2,79,107]; and Participatory Video [21–23].	Transparency promotion, open process facilitation, opportunities to transparently express opinions, task clarity, inclusiveness fostering, modeling process transparency enhancement, and clear messaging and direction.

Theme 6: Flexibility and Ease of Use

Flexibility and Ease of Use are significant benefits of 30 co-creation methods, enhancing adaptability to evolving insights and facilitating ease of modification for various contexts. Table 7 contains the sub-theme name, description, and associated methods.

Table 6. Flexibility and Ease of Use's two sub-themes and associated methods

Sub-theme	Method Name [source study]	Key Benefits
Flexibility	23 Methods: Agent-Based Models [1,2]; Bayesian Networks [2,138]; Cognitive mapping [2,113]; Cultural consensus [2]; Decision tree analyses [2]; Geographic Information Systems [2,79,107]; Daily Activity Space Travel Diary [80]; Draw and Write Technique [13–15]; Empathic Design [87]; FUBI method [143]; Fuzzy cognitive mapping [2]; Informal interviews [13]; Learner Verification and Revision [102]; Participant photography [123]; Participatory geographic mapping [80]; Participatory Impact Pathways Analysis [81]; Photovoice [22,26,32–71]; Rapid HIA [100]; Role-playing [4]; Semi-structured Interviews [88,122]; Spatial access priority mapping [101]; Stakeholder Analysis [97–99]; and Threshold Analysis [100];	Flexibility enhancement, adaptability to evolving insights, ease of modification, spontaneous application facilitation, versatility across contexts, broad applicability for health materials development, timeframe accommodation, planning type adjustment, meaningful feedback provision, in-depth topic exploration support, storyline adjustment capability, and population generalization.
Simplicity	7 Methods: Causal Loop Diagrams [2,113,114]; Decision tree analyses [2]; Participatory Design Generator Cards [124]; Partnership Data Report for Reflection [74]; Spatial access priority mapping [101]; Visioning Tool [74]; and Zaltman Metaphor Elicitation Technique [128].	Simplicity enhancement, ease of use promotion, straightforward implementation facilitation, immediate solution applicability, manageable format provision, tangible tool convenience, and user-friendly design support.

Theme 7: Impactful and Valid

Impactful and Valid benefits of 49 co-creation methods include action facilitation and logic model development, which create a foundation for sustainable outcomes and targeted resource allocation. Table 8 contains the sub-theme name, description, and associated methods.

Table 7. Impactful and Valid's three sub-themes and associated methods

Sub-theme	Method Name [source study]	Key Benefits
Impactful	18 Methods: Causal Loop Diagrams [2,113,114]; Checklist [115]; Concept mapping [6–12]; Empathic Design [87]; Geographic Information Systems [2,79,107]; Participatory Video [21–23]; Participatory/reflective photography [83]; Pathways [3]; Personas [140]; Photovoice [22,26,32–71]; Role-playing [4]; Snowball sampling [85]; Social Network Analysis [2,133]; Spatial access priority mapping [101]; Stakeholder analysis [97–99]; Strategic Environmental Assessment [91];	Action facilitation, logic model development, sustainable outcome foundation, resource targeting, community-oriented solution promotion, real-world application support, realistic problem-solving aid, stakeholder recruitment enhancement, decision-making process reform, and valuable insight generation.

	The Visioning Tool [74]; and Youth ReACT data Analysis method [144].	
Validity	31 Methods: Agent-Based Models [1,2]; Asset mapping [5]; Bayesian Networks [2,138]; Citizen's Jury [93]; Citizens' Workshop [93]; Concept mapping [6–12]; The daily activity space travel diary [80]; Decision Tree Analyses [2]; Diamond Ranking [24,90]; Focus Group [118,119]; Geographic Information Systems [2,79,107]; Learner Verification and Revision [102]; Mockups of Webpages [130]; Participatory Theme Elicitation [125]; Participatory Video [21–23]; Participatory/reflective photography [83]; Photo-elicitation [24–29]; Photo Walkabouts [30,31]; Photovoice [22,26,32–71]; Purposive sampling [85]; Q sorting [145]; Rapid HIA [100]; Reflective drawing [84]; Role-Playing Games [72]; Snowball sampling [85]; Social Network Analysis [2,133]; Sociogram [25]; Structured surveys [2]; Threshold Analysis [100]; User-driven systematic reviews [126]; and Visual Voices method [127].	Data accuracy enhancement, community strength reflection, knowledge assessment facilitation, efficacy improvement, risk exposure precision, quantitative forecasting effectiveness, preference quantification requirement, action principle evaluation, self-report data comparison, material clarification identification, researcher bias minimization, community knowledge validation, research depth enrichment, rigor maintenance, relevance assurance, stakeholder behavior highlighting, immediate data interpretation engagement, and recall inaccuracy reduction.
Skill Development	9 Methods: Bayesian Networks [2,138]; Co-design by Appropriation of Affordances[75]; Community charts [76]; Direct ranking [76]; MUST method [131]; Participatory visual methodology [82]; Photovoice [22,26,32–71]; SAT's Competence Tool [112]; and Commentary Charts [76].	Learning enhancement, mutual learning facilitation between designers and end-users, significant experience provision for teachers, teamwork skill development, learning about video design and production, co-researcher capacity building, and challenge-addressing skill improvement.

Theme 8: Reflection and Understanding

Reflection and Understanding benefits of 57 co-creation methods include promoting self-directed learning, personal transformation, and critical reflection, facilitating the exploration of gender embodiment and well-being. Table 9 contains the sub-theme name, description, and associated methods.

Table 8. Reflection and Understanding's three sub-themes and associated methods

Sub-theme	Method Name [source study]	Key Benefits
Reflection	12 Methods: Art-based narrative interview [142]; Body mapping [86]; Mānoa mash-up [106]; Modified Delphi [20]; Participatory/reflective photography [83]; The Partnership Data Report for Reflection	Promotes self-directed learning and personal transformation, facilitates critical reflection, and encourages gender embodiment and well-being

	[74]; Photo-elicitation [24–29]; Photo walkabouts [30,31]; Playback Theatre [32]; Photovoice [22,26,32–71]; Reflective drawing [84]; and SAT's Competence Tool [112].	exploration. Supports personal and social reflection, stimulates iterative reflection, fosters a reflective environment, enhances self-assessment competence, and supports progress tracking.
Understanding Complexity	35 Methods: Agent-Based Models [1,2]; Alternative scenarios [3]; Asset mapping [5]; A blended approach of photovoice and photo-elicitation [86]; Building a Model [4]; Citizen's Jury [93]; Citizens' Workshop [93]; Cognitive mapping [2,113]; Community mapping [77–79]; Cultural consensus [2]; Draw and Write Technique [13–15]; Ethnographic Facilitation [16]; The Festival [109]; Fuzzy Cognitive Mapping [2]; Geographic Information Systems [2,79,107]; Graphic Facilitation [25]; Graphs Over Time [113]; Kitchen Table Talks [30]; Mānoa Mash-up [106]; Modified Delphi [20]; MUST Method [131]; Narrative Interviews [122]; Participant Observation [88,89]; Participatory Geographic Mapping [80]; Participatory Mapping [107,108]; Personas [140]; Photo-elicitation [24–29]; Photovoice [22,26,32–71]; Role-Playing Games [72]; Social Network Analysis [2,133]; Stakeholder analysis [97–99]; Strategic Environmental Assessment [91]; The Video Diary Method [122]; Youth ReACT Data Analysis Method [144]; and Zaltman Metaphor Elicitation Technique [128].	Represents complex spatial interactions, supports feedback loops, manages uncertainty, and clarifies values. Captures dynamic service experiences, reveals nuanced values, and provides decision-makers with relevant insights. Enhances understanding of quality, care efficiency, and social issues. Captures perspectives beyond service settings, fosters immersive interaction, supports critical thinking and bridges cognitive limitations. Facilitates scenario building, and dynamic data interpretation.
Visualization	15 Methods: Community Mapping [77–79]; Concept mapping [6–12]; Geographic Information Systems [2,79,107]; Graphic facilitation [25]; Graphs over time [113]; Illustrative arts-based methodology [96]; Mind mapping [120,121]; Participatory/reflective photography [83]; Photo-elicitation [24–29]; Photo walkabouts [30,31]; Photovoice [22,26,32–71]; Sociogram [25]; Stakeholder analysis [97–99]; Structured surveys [2]; and Zaltman Metaphor Elicitation Technique [128].	Enhances exploration of higher-order constructs, articulates personal experiences, supports visual elicitation, and improves health data visualization. Facilitates spatial representation, trends analysis, and intuitive idea organization. Leverages symbols, metaphors, and aesthetic dimensions to increase visual literacy. Stimulates discussion, reveals interaction patterns, raises issue visibility, and provides rich visual insights when conducted with rigor and reflexivity.

Theme 9: Efficient and Strategic

Efficient and Strategic benefits of 15 co-creation methods include time efficiency and effective group discussion facilitation, minimizing costs while ensuring a comprehensive approach. Table 10 contains the sub-theme name, description, and associated methods.

Table 9. Efficient and Strategic's two sub-themes and associated methods

Sub-theme	Method Name [source study]	Key Benefits
Efficient	10 Methods: Fuzzy cognitive mapping [2]; Metaplan method [19]; Participant photography [123]; Participatory Impact Pathways Analysis [81]; Participatory Theme Elicitation [125]; Photovoice [22,26,32–71]; Spatial access priority mapping [101]; Structured surveys [2]; Threshold Analysis [100]; and Zaltman Metaphor Elicitation Technique [128].	Promotes time efficiency, facilitates effective group discussions, minimizes costs, and ensures a comprehensive approach. Supports rapid assessment, integrates local insights, and operates with minimal staff assistance. Offers a cost-effective and streamlined process for addressing multiple issues efficiently.
Strategic	5 Methods: Bayesian Networks [2,138]; Causal Loop Diagrams [2,113,114]; Decision tree analyses [2]; Pathways [3]; and Storytelling [134,135].	Supports strategic considerations, provides an aggregate view of problem structures, and maintains focus on feedback loops. Facilitates understanding of future actions, aids in mitigation strategy development, enhances problem-solving capabilities, and minimizes cross-cultural barriers in the research process.

Method Challenges

Data was extracted per method regarding the benefits and potential challenges when using the method: 90 studies reported the challenges of using 78 methods, and the following is a summary of the findings in grouped 6 themes and 25 sub-themes

Theme 1: Engagement and Participation

The 42 co-creation methods in this theme include engagement and participation challenges with stakeholder cooperation, limited interaction with policymakers, and participant reluctance, particularly in visual activities. Table 11 contains the sub-theme name, description, and associated methods.

Table 10. Engagement and Participation's six sub-themes and associated methods

Sub-theme	Method Name [source study]	Key Challenges
Poor engagement	8 Methods: Creative Practice [94]; Diamond Ranking [24,90]; Informal interviews [13]; Interpretive Focus Group [95]; Photo-elicitation [24,25,27–29,146]; Photovoice [33,35,36,38,39,41,46–48,50,52,53,55–64,66–68,70,147–149]; Purposive Sampling [85]; and Youth ReACT data analysis method [144].	Faces challenges with stakeholder cooperation, limited interaction with policymakers, and reluctance from some participants. Parental presence may constrain children's participation, and reluctance to engage can limit data collection. Some participants, especially adults, resist visual activities, and reliance on regular engagement can reduce participant-driven data analysis, conflicting with participatory principles.
Poor recruitment	3 Methods: Body Mapping [86]; Creative Practice [94]; and Learner Verification and Revision [102].	Presents recruitment challenges, especially with snowballing techniques, and reliance on village leadership and local research assistants can impact participant selection.
Unmotivated	3 Methods: Informal interviews [13]; Mānoa Mash-Up Method [106]; and video diary method [122].	Presents challenges with high rejection rates, especially among children, due to lack of time, strength, or motivation. Risks reduced participation if individuals do not see direct benefits, and children may become bored with verbal interactions when used in isolation.
Requires facilitation	7 Methods: Alternative Scenarios [3]; Checklists [115]; Collaborative Songwriting [77]; Dialogic Art (Relational Art) [150]; Draw and Write/Talk Technique [151]; Participatory Design Generator Cards [124]; and Youth ReACT (Research Actualizing Critical Thought) data analysis method [144].	Challenges requiring skilled facilitators to maintain focused discussions while allowing free expression, with participants often relying on project team guidance and children needing encouragement to participate. The method's effectiveness depends on facilitators who can guide discussions and tailor approaches to specific contexts, necessitating strong facilitation skills.
Requires Skill or Training	15 Methods: Asset Mapping [5]; Bayesian Networks [2,138]; Causal Loop Diagrams [2,114]; Draw and Write Technique [13,14]; Geographic Information Systems [2,79,107]; Graphic Facilitation [25]; Learner Verification and Revision [102]; Mandala Drawing [110]; Participatory Threats Assessment [141]; Photovoice	Challenges requiring extensive training and specialized skills, including artistic abilities and technical proficiency, complexity in data collection and analysis, inadequate training leading to errors, mastery of arts-based methods, handling

	[33,35,36,38,39,41,46–48,50,52,53,55–64,66–68,70,147–149]; Sociogram [25]; Strategic Environmental Assessment [91]; Threshold Analysis [100]; Visual Voices method [127]; and The Youth ReACT (Research Actualizing Critical Thought) data analysis method [144].	stakeholder perception data, proficiency in diverse media, and attention to conversational nuances.
Group dynamics	8 Methods: Art Making [4]; Photo-elicitation [24,25,27–29,146]; Photovoice [33,35,36,38,39,41,46–48,50,52,53,55–64,66–68,70,147–149]; Playback Theatre [32]; Rapid HIA [100]; Semi-participant observations [13]; Transdisciplinarity [73]; and The Youth ReACT (Research Actualizing Critical Thought) data analysis method [144].	Challenges from social influence, where children may mimic peers or conform to researcher expectations, researcher-participant dynamics affecting outcomes, difficulties initiating discussions, observer presence altering natural behaviors, adolescent group dynamics requiring patience, and partnerships shaped by specific issues.

Theme 2: Resources and Practical Constraints

The 45 co-creation methods in this theme face resources and practical constraints associated with funding, which hinder covering essential costs like training, staff time, and participant incentives. Table 12 contains the sub-theme name, description, and associated methods.

Table 11. Resources and Practical Constraints' four sub-themes and associated methods

Sub-theme	Method Name [source study]	Key Challenges
Insufficient funds	7 methods: Body Mapping [86]; Causal Loop Diagrams [2,114]; Citizens' Jury [93]; Collaborative Songwriting [77]; Creative Practice [94]; Learner Verification and Revision [102]; and Participant Photography [123].	Funding challenges for training, staff time, participant incentives, and material production, with limited resources hindering effective implementation and evaluation, making the method too expensive for some researchers.
Resource-intensive	4 Methods: Photo-elicitation [24,25,27–29,146]; Photovoice [33,35,36,38,39,41,46–48,50,52,53,55–64,66–68,70,147–149]; Threshold Analysis [100]; and Transdisciplinarity [73].	Resource-intensive challenges, particularly with large participant groups and multiple time points, substantial resources for web-based health impact assessment tools, and budgetary discussions in intercultural settings complicating the process.
Logistical constraints	10 Methods: Art Making [4]; Empathic design [87]; Gerographic Information Systems [2,79,107]; Learner Verification and Revision [102]; Participant Photography [123]; Photovoice [33,35,36,38,39,41,46–	Logistical constraints, significant organization for activities, challenges in accessing confidential data, careful planning for guides and

	48,50,52,53,55–64,66–68,70,147–149]; Role Playing [4]; Role-Playing Games [72]; Structured surveys [2]; and The Zaltman Metaphor Elicitation Technique [128].	scheduling, complications with camera use and photo selection, managing large data volumes, capturing information during events, and participant preference for gathering images.
Time-intensive	26 Methods: Asset Mapping [5]; Bayesian Networks [138]; Body Mapping [86]; Citizens' Jury [93]; Co-design by Appropriation of Affordances [75]; Collaborative Songwriting [77]; Commentary Charts [76]; Creative Practice [94]; Direct Ranking [76]; Empathic Design [87]; Interpretive Focus Group [95]; Learner Verification and Revision [102]; Mānoa Mash-Up Method [106]; Participant Photography [123]; Participatory Design Generator Cards [124]; Participatory Impact Pathways Analysis [81]; Photo-elicitation [24,25,27–29,146]; Photovoice [33,35,36,38,39,41,46–48,50,52,53,55–64,66–68,70,147–149]; Purposive Sampling [85]; Video Diary Method [122]; Role-Playing Games [72]; Social Network Analysis [133]; Stick-a-Star Quiz [13]; Visual Voices method [127]; The Youth ReACT data analysis method [144]; The Zaltman Metaphor Elicitation Technique [128].	Challenges due to being time-intensive, especially for lengthy processes like interviews and video diaries, requiring significant time commitment from participants and professionals. This complicates integration into decision-making and evaluation, with time constraints hindering effective participation and engagement, leading to perceptions of methods as labor-intensive and slow, diminishing their overall value.

Theme 3: Trust and Transparency

Trust and Transparency challenges associated with 28 co-creation methods include participants from welfare and therapy backgrounds fearing potential 'diagnosis' through art, which can hinder their engagement. Table 13 contains the sub-theme name, description, and associated methods.

Table 12. Trust and Transparency's four sub-themes and associated methods

Sub-theme	Method Name [source study]	Key Challenges
Distrust	3 Methods: Illustrative Arts-Based Methodology [96]; Participatory/Reflective Photography [83]; and Snowball sampling [85].	Challenges arise from participants with welfare and therapy backgrounds fearing that art may be used to 'diagnose' or 'analyze' them, which impacts engagement. The emphasis on textual research can hinder the adoption of visual methods, while concerns about privacy and confidentiality may lead participants to withhold information.

Low transparency	3 Methods: Agent-Based Modeling [1,2]; Bayesian Networks [2,138]; and Direct Ranking [76].	Presents challenges due to low transparency, leading to doubts about accuracy and effectiveness. Unclear outcomes or goals can further complicate participants' understanding of the method.
Misunderstanding	9 Methods: Art Making [4]; Bayesian Networks [2,138]; Building a Model [4]; Causal Loop Diagrams [2,114]; Draw and Write Technique [13,14]; Participatory Threat Assessment [141]; Sociogram [25]; Threshold Analysis [100]; and Wild Cards method [132].	Challenges arise as children's artwork may seem unrelated to the context, necessitating exploration of connections. Communicating results is difficult due to the blend of real and imaginary elements, which can obscure lived experiences. Flawed mental models and a lack of shared understanding of variables may result in shallow diagrams and misinterpretations. Densely drawn sociograms complicate analysis, and participants often find wild cards abstract or difficult to understand.
Ethical concerns	6 Methods: Draw and Write Technique [13,14]; Participant Photography [123]; Photovoice [33,35,36,38,39,41,46–48,50,52,53,55–64,66–68,70,147–149]; Semi-structured interviews [122]; Stick-a-Star Quiz [13]; and Transdisciplinarity [73].	Raises issues of confidentiality and ownership, necessitating extensive consent forms and careful handling of sensitive imagery. Challenges include risks of stigmatization, bias from professionals in impact descriptions, and difficulties in capturing negative social concepts. Negotiating differing definitions of "value," "compensation," and "fairness" complicates ethical considerations.
Emotionally draining	5 Methods: Draw and Write Technique [13,14]; Forum Theatre [32]; Illustrative Arts-Based Methodology [96]; Mandala Drawing [110]; and Photovoice [33,35,36,38,39,41,46–48,50,52,53,55–64,66–68,70,147–149].	Can be emotionally taxing for participants, potentially inducing negative feelings and anxiety. Requires sensitivity to manage emotional responses effectively, especially when scenarios selected may be repressive or aggressive. Participants may feel uncomfortable with unfamiliar methods and experience anxiety about drawing or taking "proper" photographs due to fear of negative judgment.

Theme 4: Methodological Limitations

Methodological limitations in 50 co-creation methods hinder research integrity, with weak analytic rigor and limited predictive accuracy raising concerns about reliability. Table 14 contains the sub-theme name, description, and associated methods.

Table 13. Methodological Limitations' six sub-themes and associated methods

Sub-theme	Method Name [source study]	Key Challenges
Not robust	17 Methods: Co-design by Appropriation of Affordances [75]; Concept Mapping [6–8,10,11,152]; Concept Mapping [6–8,10,11,152]; Consensus Conference [153]; Daily Activity Space Travel Diary [80]; Empathic Design [87]; Festival [109]; Interpretive Focus Group [95]; Narrative Interview [122]; Participant Photography [123]; Particpatory Design Generator Cards [124]; Participatory Geographic Mapping [80]; Participatory/Reflective Photography [83]; Photo-elicitation [24,25,27–29,146]; Plan Review Checklists [100]; Stakeholder Analysis [98,99]; and Threshold Analysis [100].	Presents robustness challenges, requiring further testing for reliability and validity. Criticized for weak analytic rigor and limitations in predictive accuracy. It faces issues with recall delays, reliance on convenience sampling, and insufficient statistical analysis. Modifications to reduce time can affect effectiveness, while self-censorship, spatial imprecision, and outdated materials further limit robustness and depth of analysis.
Not representative	15 Methods: Citizens' Jury [93]; Citizens' Workshop [93]; Concept Mapping [6–8,10,11,152]; Dialogic Art [150]; Direct Ranking [76]; Mānoa Mash-Up Method [106]; Participatory Mapping [107,108]; Photo-elicitation [24,25,27–29,146]; Photovoice [33,35,36,38,39,41,46–48,50,52,53,55–64,66–68,70,147–149]; Purposive Sampling [85]; Snowball Sampling [85]; Social Network Analysis [133]; User-driven Systematic Reviews [126]; Video Diary Method [122]; and the Zaltman Metaphor Elicitation Technique [128].	Faces challenges in achieving representativeness due to small sample sizes, potentially biased data from highly motivated participants, and difficulties in engaging a diverse group. May overlook specific perspectives, particularly from adults or older populations. Self-selection can lead to non-representative viewpoints, and the method may struggle to engage diverse or hard-to-reach groups, limiting the diversity of participant insights.
Limited generalizability	3 Methods: Dialogic Art [150]; Visual Voices Method [127]; and the Zaltman Metaphor Elicitation Technique [128].	Limits the generalizability of results due to challenges in applying findings broadly, potentially reducing the relevance of insights beyond the specific sample or context.
Limited data	16 Methods: Concept Mapping [6–8,10,11,152]; Draw and Write Technique [13–15]; Decision Tree Analyses [2]; Future Workshop [124]; Fuzzy Cognitive Mapping [2]; Geographic Information Systems [2,79,107]; Informal Interviews [13];	Faces challenges with limited data due to reduced image sets affecting categorization and reliance on specific periods. Produces incomplete narratives, lacks longitudinal insights, and

	Interpretive Focus Group [95]; Participatory Mapping [107,108]; Photo-elicitation [24,25,27–29,146]; Playback Theatre [32]; SAT's Competence Tool [112]; Semi-structured Interviews [122]; Stick-a-Star Quiz [13]; Strategic Environmental Assessment [91]; and the Zaltman Metaphor Elicitation Technique [128].	has poor temporal and spatial representation. Engagement with only small portions of data limits in-depth responses, often requiring integration with other methods for deeper analysis.
No evaluation	3 Methods: Creative Practice [94]; Five Whys Method [136]; and User Stories [154].	Faces challenges due to insufficient time for evaluating long-term benefits. Limited applicability beyond specific settings, such as youth participatory action research in schools. Lacks empirical studies on use and effectiveness, hindering assessment of quality improvements.
No impact	2 Methods: Pathways [3]; and Social Network Analysis [133].	Generated no uptake from the local government, which showed little interest in community input. Some centralities lacked meaningful or useful significance, limiting overall impact.

Theme 5: Systemic and Structural Barriers

Systematic and structural barriers in 32 co-creation methods limit bottom-up planning by restricting decision-making power at the community level, making engagement difficult. Table 15 contains the sub-theme name, description, and associated methods.

Table 14. Systemic and Structural Barriers' three sub-themes and associated methods

Sub-theme	Method Name [source study]	Key Challenges
System barriers	7 Methods: Geographic Information Systems [2,79,107]; Participatory Georgraphic Mapping [80]; Social Network Analysis [133]; Sociogram [25]; Stick-a-Star Quiz [13]; Strategic Environmental Assessment [91]; and Visioning [3].	Challenges limit bottom-up planning due to insufficient devolved decision-making power, hindering community engagement and complicating efforts amid future uncertainties. Issues arise from formal decision-making procedures, geographic scope, technical differences across sectors, and complex coding systems, complicating real-time data recording. Visual interactions are reduced to numerical tallies, limiting meaningful insights into community integration. Outdated

		satellite imagery leads to biased exposure estimates, and difficulties in explaining centrality degrees further limit overall impact.
Rigid	8 Methods: Bayesian Networks [2,138]; Checklists [115]; Concept Mapping [6–8,10,11,152]; Decision Tree Analyses [2]; Forum Theatre [32]; Fuzzy Cognitive Mapping [2]; Focus Groups [155]; and Geographic Information Systems [2,79,107].	Challenges arise from rigidity, struggling to handle uncertainty and requiring adaptation to incorporate creative, activity-based approaches. Criticism stems from limitations imposed by narrow academic boundaries, hindering the ability to capture evolving systems or discrete temporal changes. Difficulties in dynamic environments incur significant computational burdens as the number of variables increases. The typical top-down sequence restricts flexibility, while the structured nature may lead to a loss of in-depth insights, limiting effectiveness in social conflict situations.
Inaccessible	18 Methods: Alternative Scenarios [3]; Blended Approach of Photovoice and Photo-Elicitation [86]; Body Mapping [86]; Building a Model [4]; Community Charts [76]; Daily Activity Space Travel Diary [80]; Direct Ranking [76]; Fuzzy Cognitive Mapping [2]; Geographic Information Systems [2,79,107]; Learner Verification and Revision [102]; Illustrative Arts-Based Methodology [96]; Participatory Georgraphic Mapping [80]; Photo-elicitation [24,25,27–29,146]; Rapid HIA [100]; Sociogram [25]; Transdisciplinarity [73]; Visioning with the CBPR model [74]; and the Zaltman Metaphor Elicitation Technique [128].	Challenges related to accessibility arise from participant requirements, leading to reduced engagement. The method's complexity, including dense text and a lack of reflexivity, hinders understanding and may alienate individuals with specific health needs. It may not suit all participants, as complex equipment can deter involvement, and map-reading challenges may exclude some. Activity space methods often lack clear indices for community integration, complicating the analysis of social dynamics. Additionally, the method's public nature and the perception of arts-based self-expression as Westernized may leave some participants feeling like outsiders.

Theme 6: Focus and Commitment

Challenges related to focus and commitment in 14 co-creation methods hinder successful implementation, requiring substantial dedication from participants, researchers, and institutions. Table 16 contains the sub-theme name, description, and associated methods.

Table 15. Focus and Commitment's three sub-themes and associated methods

Sub-theme	Method Name [source study]	Key Challenges
Needs Commitment	3 Methods: Concept Mapping [6–8,10,11,152]; Purposive Sampling [85]; and Rapid HIA [100].	Requires significant commitment from participants, researchers, and institutions, often necessitating guidance from a community advisory board to prioritize information collection and dissemination about the project and related health issues prior to the workshop.
Topic drifting	3 Methods: Bayesian Networks [2,138]; Informal Interviews [13]; and Participatory Threats Assessment [141].	Cultural peculiarities can cause participants to drift toward side issues, diverting focus from the main process and risking the loss of children's authentic voices. This challenge is exacerbated by the method's tendency to capture threats across broad regions and extended timeframes, making it crucial to gather accurate perceptions. Inaccurate perceptions may lead to proposed interventions that do not align with stakeholders' views, resulting in potential opposition.
Disconnect	Methods: Consensus Conference [153]; Creative Practice [94]; Diamond Ranking [24,90]; Forum Theatre [32]; Participatory/Reflective Photography [83]; and Photovoice [33,35,36,38,39,41,46–48,50,52,53,55–64,66–68,70,147–149].	Challenges arise from imbalances between parties and knowledge asymmetry due to a lack of shared background materials before the conference, complicating the integration of diverse opinions. Limited community embedding and insufficient time for conventional measures can hinder productive negotiation and resolution. Additionally, the perceived complexity of detailed quasi-quantitative frameworks for analyzing photographs may complicate efforts to attribute policy changes to specific

		interventions, further impeding effective collaboration.

References

1. Smetschka B, Gaube V. Co-creating formalized models: Participatory modelling as method and process in transdisciplinary research and its impact potentials. Environ Sci Policy. 2020;103:41–9.

2. Voinov A, Jenni K, Gray S, Kolagani N, Glynn PD, Bommel P, et al. Tools and methods in participatory modeling: Selecting the right tool for the job. Environ Model Softw. 2018;109:232–55.

3. Evans K, Jong W de, Cronkleton P, Nghi TH. Participatory Methods for Planning the Future in Forest Communities. Soc Nat Resour. 2010;23:604–19.

4. Green C. Four Methods for Engaging Young Children as Environmental Education Researchers. Int J Emerg Issues Early Child Educ. 2023;5:6.

5. Asset Mapping as a Research Tool for Community-Based Participatory Research in Social Work | Social Work Research | Oxford Academic [Internet]. [cited 2024 Sep 28]. Available from: https://academic.oup.com/swr/article-abstract/38/1/59/1617653?redirectedFrom=fulltext

6. Haque N, Rosas S. Concept Mapping of Photovoices: Sequencing and Integrating Methods to Understand Immigrants' Perceptions of Neighborhood Influences on Health. Fam Community Health. 2010;33:193.

7. Thompson JR, Burke JG. Increasing Community Participation in Public Health Research: Applications for Concept Mapping Methodology. Prog Community Health Partnersh Res Educ Action. 2020;14:243–50.

8. Ahmad F, Norman C, O'Campo P. What is needed to implement a computer-assisted health risk assessment tool? An exploratory concept mapping study. BMC Med Inform Decis Mak. 2012;12:149.

9. Windsor LC. Using Concept Mapping in Community-Based Participatory Research: A Mixed Methods Approach. J Mix Methods Res. 2013;7:274–93.

10. Burke JG, O'Campo P, Peak GL, Gielen AC, McDonnell KA, Trochim WMK. An introduction to concept mapping as a participatory public health research method. Qual Health Res. 2005;15:1392–410.

11. Vaughn LM, Jones JR, Booth E, Burke JG. Concept mapping methodology and community-engaged research: A perfect pairing. Eval Program Plann. 2017;60:229–37.

12. Scholz G, Dewulf A, Pahl-Wostl C. An Analytical Framework of Social Learning Facilitated by Participatory Methods. Syst Pract Action Res. 2014;27:575–91.

13. Lambert V, Glacken M, McCarron M. Using a range of methods to access children's voices. J Res Nurs. 2013;18:601–16.

14. Sewell K. Researching sensitive issues: a critical appraisal of 'draw-and-write' as a data collection technique in eliciting children's perceptions. Int J Res Method Educ. 2011;34:175–91.

15. Haijes HA, van Thiel GJMW. Participatory methods in pediatric participatory research: a systematic review. Pediatr Res. 2016;79:676–83.

16. Hartwig RT. Ethnographic Facilitation as a Complementary Methodology for Conducting Applied Communication Scholarship. J Appl Commun Res. 2014;42:60–84.

17. Pereira VR, Coimbra VCC, Cardoso C de S, Oliveira NA, Vieira ACG, Nobre M de O, et al. Participatory methodologies in research with children: creative and innovative approaches. Rev Gaucha Enferm. 2017;37:e67908.

18. Lahtinen M, Nenonen S, Rasila H, Lehtelä J, Ruohomäki V, Reijula K. Rehabilitation centers in change: participatory methods for managing redesign and renovation. HERD. 2014;7:57–75.

19. Calba C, Ponsich A, Nam S, Collineau L, Min S, Thonnat J, et al. Development of a participatory tool for the evaluation of Village Animal Health Workers in Cambodia. Acta Trop. 2014;134:17–28.

20. Revez A, Dunphy N, Harris C, Mullally G, Lennon B, Gaffney C. Beyond Forecasting: Using a Modified Delphi Method to Build Upon Participatory Action Research in Developing Principles for a Just and Inclusive Energy Transition. Int J Qual Methods. 2020;19:160940692090321.

21. Tremblay C. Towards inclusive waste management: participatory video as a communication tool. Proc Inst Civ Eng - Waste Resour Manag. 2013;166:177–86.

22. D'Amico M, Denov M, Khan F, Linds W, Akesson B. Research as intervention? Exploring the health and well-being of children and youth facing global adversity through participatory visual methods. Glob Public Health. 2016;11:528–45.

23. Lomax H, Fink J, Singh N, High C. The politics of performance: methodological challenges of researching children's experiences of childhood through the lens of participatory video. Int J Soc Res Methodol. 2011;14:231–43.

24. Woolner P, Clark J, Hall E, Tiplady L, Thomas U, Wall K. Pictures are necessary but not sufficient: Using a range of visual methods to engage users about school design. Learn Environ Res. 2010;13:1–22.

25. North N, Sieberhagen S, Leonard A, Bonaconsa C, Coetzee M. Making Children's Nursing Practices Visible: Using Visual and Participatory Techniques to Describe Family Involvement in the Care of Hospitalized Children in Southern African Settings. Int J Qual Methods. 2019;18:1609406919849324.

26. Lorenz LS, Kolb B. Involving the public through participatory visual research methods. Health Expect. 2009;12:262–74.

27. Najib Balbale S, Schwingel A, Chodzko-Zajko W, Huhman M. Visual and Participatory Research Methods for the Development of Health Messages for Underserved Populations. Health Commun. 2014;29:728–40.

28. Due C, Riggs DW, Augoustinos M. Research with Children of Migrant and Refugee Backgrounds: A Review of Child-Centered Research Methods. Child Indic Res. 2014;7:209–27.

29. Sebastião E, Gálvez PAE, Bobitt J, Adamson BC, Schwingel A. Visual and participatory research techniques: photo-elicitation and its potential to better inform public health about physical activity and eating behavior in underserved populations. J Public Health. 2016;24:3–7.

30. Lang A. Seniors managing multiple medications: using mixed methods to view the home care safety lens. BMC Health Serv Res [Internet]. [cited 2024 Sep 10]; Available from: https://bmchealthservres.biomedcentral.com/articles/10.1186/s12913-015-1193-5

31. Use of participatory visual narrative methods to explore older adults' experiences of managing multiple chronic conditions during care transitions | BMC Health Services Research | Full Text [Internet]. [cited 2024 Sep 29]. Available from: https://bmchealthservres.biomedcentral.com/articles/10.1186/s12913-018-3292-6

32. Kaptani E, Yuval-Davis N. Participatory Theatre as a Research Methodology: Identity, Performance and Social Action among Refugees. Sociol Res Online. 2008;13:1–12.

33. Molloy JK. Photovoice as a Tool for Social Justice Workers. J Progress Hum Serv. 2007;18:39–55.

34. Walker A, Oomen-Early J. Do You See What I See? Using Photovoice to Teach Health Education Students the Value of Using Community-Based Participatory Methods. J Health Educ Teach Tech. 2014;1:41–52.

35. Valiquette-Tessier S-C, Vandette M-P, Gosselin J. In Her Own Eyes: Photovoice as an Innovative Methodology to Reach Disadvantaged Single Mothers. Can J Commun Ment Health. 2015;34:1–16.

36. Rania N, Coppola I, Pinna L. Adapting Qualitative Methods during the COVID-19 Era: Factors to Consider for Successful Use of Online Photovoice. Qual Rep. 2021;26:2711–29.

37. Saunders G, Dillard L, Frederick M, Silverman S. Examining the Utility of Photovoice as an Audiological Counseling Tool. J Am Acad Audiol. 2019;30.

38. Sutton-Brown CA. Photovoice: A Methodological Guide.

39. Lal S, Jarus T, Suto MJ. A Scoping Review of the Photovoice Method: Implications for Occupational Therapy Research. Can J Occup Ther. 2012;79:181–90.

40. Drawson AS, Toombs E, Mushquash CJ. Indigenous Research Methods: A Systematic Review. Int Indig Policy J [Internet]. 2017 [cited 2024 Sep 10];8. Available from: https://ojs.lib.uwo.ca/index.php/iipj/article/view/7515

41. Teti M, Murray C, Johnson L, Binson D. Photovoice as a Community-Based Participatory Research Method among Women Living with HIV/AIDS: Ethical Opportunities and Challenges. J Empir Res Hum Res Ethics. 2012;7:34–43.

42. Campbell RB, Larsen M, DiGiandomenico A, Davidson MA, Booth GL, Hwang SW, et al. The challenges of managing diabetes while homeless: a qualitative study using photovoice methodology. CMAJ. 2021;193:E1034–41.

43. Madrigal DS, Salvatore A, Casillas G, Casillas C, Vera I, Eskenazi B, et al. Health in My Community: Conducting and Evaluating PhotoVoice as a Tool to Promote Environmental Health and Leadership Among Latino/a Youth. Prog Community Health Partnersh Res Educ Action. 2014;8:317–29.

44. MacFarlane EK, Shakya R, Berry HL, Kohrt BA. Implications of participatory methods to address mental health needs associated with climate change: 'photovoice' in Nepal. BJPsych Int. 2015;12:33–5.

45. Houle J, Coulombe S, Radziszewski S, Leloup X, Saïas T, Torres J, et al. An intervention strategy for improving residential environment and positive mental health among public housing tenants: rationale, design and methods of Flash on my neighborhood! BMC Public Health. 2017;17:737.

46. Aw S, Koh GC, Oh YJ, Wong ML, Vrijhoef HJ, Harding SC, et al. Interacting with place and mapping community needs to context: Comparing and triangulating multiple geospatial-qualitative methods using the Focus–Expand–Compare approach. Methodol Innov. 2021;14:205979912098777.

47. Nykiforuk CIJ, Vallianatos H, Nieuwendyk LM. Photovoice as a Method for Revealing Community Perceptions of the Built and Social Environment. Int J Qual Methods. 2011;10:103–24.

48. Kramer L, Schwartz P, Cheadle A, Rauzon S. Using Photovoice as a Participatory Evaluation Tool in Kaiser Permanente's Community Health Initiative. Health Promot Pract. 2013;14:686–94.

49. Downey LH, Ireson CL, Scutchfield FD. The Use of Photovoice as a Method of Facilitating Deliberation. Health Promot Pract. 2009;10:419–27.

50. Pauwels L. 'Participatory' visual research revisited: A critical-constructive assessment of epistemological, methodological and social activist tenets. Ethnography. 2015;16:95–117.

51. Engaging Adolescents Through Participatory and Qualitative Research Methods to Develop a Digital Communication Intervention to Reduce Adolescent Obesity - William C. Livingood, David Monticalvo, Jay M. Bernhardt, Kelli T. Wells, Todd Harris, Kadra Kee, Johnathan Hayes, Donald George, Lynn D. Woodhouse, 2017 [Internet]. [cited 2024 Sep 29]. Available from: https://journals.sagepub.com/doi/10.1177/1090198116677216

52. Golden T. Reframing Photovoice: Building on the Method to Develop More Equitable and Responsive Research Practices. Qual Health Res. 2020;30:960–72.

53. Baker TA, Wang CC. Photovoice: Use of a Participatory Action Research Method to Explore the Chronic Pain Experience in Older Adults. Qual Health Res. 2006;16:1405–13.

54. Johnson LR, Drescher CF, Assenga SH, Marsh RJ. Assessing Assets Among Street-Connected Youth: New Angles With Participatory Methods in Tanzania. J Adolesc Res. 2019;34:619–51.

55. Bisung E, Elliott SJ, Abudho B, Karanja DM, Schuster-Wallace CJ. Using Photovoice as a Community Based Participatory Research Tool for Changing Water, Sanitation, and Hygiene Behaviours in Usoma, Kenya.

56. Marshalsey L, Sclater M. Arts-Based Educational Research: The Challenges of Social Media and Video-Based Research Methods in Communication Design Education.

57. Maclean K, Woodward E. Photovoice Evaluated: An Appropriate Visual Methodology for Aboriginal Water Resource Research. Geogr Res. 2013;51:94–105.

58. Witkowski K, Matiz Reyes A, Padilla M. Teaching diversity in public participation through participatory research: A case study of the PhotoVoice methodology. J Public Aff Educ. 2021;27:218–37.

59. Fay F. The impact of the school space on research methodology, child participation and safety: views from children in Zanzibar. Child Geogr. 2018;16:405–17.

60. Umurungi J-P, Mitchell C, Gervais M, Ubalijoro E, Kabarenzi V. Photovoice as a Methodological Tool to Address HIV and AIDS and Gender Violence amongst Girls on the Street in Rwanda. J Psychol Afr. 2008;18:413–9.

61. Barry J, Higgins A. PhotoVoice: An Ideal Methodology for Use within Recovery-Oriented Mental Health Research. Issues Ment Health Nurs. 2021;42:676–81.

62. Capous-Desyllas M, Forro VA. Tensions, Challenges, and Lessons Learned: Methodological Reflections From Two Photovoice Projects With Sex Workers. J Community Pract. 2014;22:150–75.

63. Sitter KC. Taking a Closer Look at Photovoice as a Participatory Action Research Method. J Progress Hum Serv. 2017;28:36–48.

64. Peabody CG. Using Photovoice as a Tool to Engage Social Work Students in Social Justice. J Teach Soc Work. 2013;33:251–65.

65. Russinova Z, Mizock L, Bloch P. Photovoice as a tool to understand the experience of stigma among individuals with serious mental illnesses. Stigma Health. 2018;3:171–85.

66. Novek S, Morris-Oswald T, Menec V. Using photovoice with older adults: some methodological strengths and issues. Ageing Soc. 2012;32:451–70.

67. Ronzi S, Pope D, Orton L, Bruce N. Using photovoice methods to explore older people's perceptions of respect and social inclusion in cities: Opportunities, challenges and solutions. SSM - Popul Health. 2016;2:732–45.

68. Krutt H, Dyer L, Arora A, Rollman J, Jozkowski AC. PhotoVoice is a feasible method of program evaluation at a center serving adults with autism. Eval Program Plann. 2018;68:74–80.

69. Doucet M, Pratt H, Dzhenganin M, Read J. Nothing About Us Without Us: Using Participatory Action Research (PAR) and arts-based methods as empowerment and social justice tools in doing research with youth "aging out" of care. Child Abuse Negl. 2022;130:105358.

70. Participatory Methodologies With Adolescents: A Research Approach Used to Explore Structural Factors Affecting Alcohol Use and Related Unsafe Sex in Tanzania | Journal of Prevention [Internet]. [cited 2024 Sep 29]. Available from: https://link.springer.com/article/10.1007/s10935-020-00586-0

71. Foster-Fishman P, Nowell B, Deacon Z, Nievar MA, McCann P. Using methods that matter: the impact of reflection, dialogue, and voice. Am J Community Psychol. 2005;36:275–91.

72. Vieira Pak M, Castillo Brieva D. Designing and implementing a Role-Playing Game: A tool to explain factors, decision making and landscape transformation.

73. Berger-González M, Stauffacher M, Zinsstag J, Edwards P, Krütli P. Transdisciplinary Research on Cancer-Healing Systems Between Biomedicine and the Maya of Guatemala: A Tool for Reciprocal Reflexivity in a Multi-Epistemological Setting.

74. Engage for Equity: Development of Community-Based Participatory Research Tools - Myra Parker, Nina Wallerstein, Bonnie Duran, Maya Magarati, Ellen Burgess, Shannon Sanchez-Youngman, Blake Boursaw, Amanda Heffernan, Justin Garoutte, Paul Koegel, 2020 [Internet]. [cited 2023 Aug 17]. Available from: https://journals.sagepub.com/doi/abs/10.1177/1090198120921188?journalCode=hebc

75. Dennerlein SM, Tomberg V, Treasure-Jones T, Theiler D, Lindstaedt S, Ley T. Co-designing tools for workplace learning. Inf Learn Sci. 2020;121:175–205.

76. O'Reilly-de Brún M, de Brún T, O'Donnell CA, Papadakaki M, Saridaki A, Lionis C, et al. Material practices for meaningful engagement: An analysis of participatory learning and action

research techniques for data generation and analysis in a health research partnership. Health Expect. 2018;21:159–70.

77. Fairchild R, McFerran KS. Understanding Children's Resources in the Context of Family Violence through a Collaborative Songwriting Method. Child Aust. 2018;43:255–66.

78. Amsden J, VanWynsberghe R. Community mapping as a research tool with youth. Action Res. 2005;3:357–81.

79. Beyer KMM, Comstock S, Seagren R. Disease maps as context for community mapping: a methodological approach for linking confidential health information with local geographical knowledge for community health research. J Community Health. 2010;35:635–44.

80. Felker-Kantor E, Polanco C, Perez M, Donastorg Y, Andrinopoulos K, Kendall C, et al. Participatory geographic mapping and activity space diaries: innovative data collection methods for understanding environmental risk exposures among female sex workers in a low-to middle-income country. Int J Health Geogr. 2021;20:25.

81. Ekirapa-Kiracho E, Ghosh U, Brahmachari R, Paina L. Engaging stakeholders: lessons from the use of participatory tools for improving maternal and child care health services. Health Res Policy Syst. 2017;15:106.

82. Wood L, Olivier T. Video production as a tool for raising educator awareness about collaborative teacher–parent partnerships. Educ Res. 2011;53:399–414.

83. Gerodimos R. Youth and the City: Reflective Photography as a Tool of Urban Voice. J Media Lit Educ. 2018;10:82–103.

84. Calvo M. "Reflective drawing": Drawing as a tool for reflection in design research. 2016.

85. Comparing two sampling methods to engage hard-to-reach communities in research priority setting | BMC Medical Research Methodology | Full Text [Internet]. [cited 2024 Sep 29]. Available from: https://bmcmedresmethodol.biomedcentral.com/articles/10.1186/s12874-016-0242-z

86. Switzer S, Guta A, de Prinse K, Chan Carusone S, Strike C. Visualizing harm reduction: Methodological and ethical considerations. Soc Sci Med 1982. 2015;133:77–84.

87. Using Empathic Design as a Tool for Urban Sustainability in Low-Resource Settings [Internet]. [cited 2024 Sep 28]. Available from: https://www.mdpi.com/2071-1050/10/7/2493

88. Hemming PJ. Mixing Qualitative Research Methods in Children's Geographies. Area. 2008;40:152–62.

89. Mullings L, Wali A, McLean D, Mitchell J, Prince S, Thomas D, et al. Qualitative methodologies and community participation in examining reproductive experiences: the Harlem Birth Right Project. Matern Child Health J. 2001;5:85–93.

90. Hill V, Croydon A, Greathead S, Kenny L, Yates R, Pellicano E. Research methods for children with multiple needs: Developing techniques to facilitate all children and young people to have 'a voice.' Educ Child Psychol. 2016;33:26–43.

91. Cerreta M, De Toro P. Integrated spatial assessment for a creative decision-making process: a combined methodological approach to strategic environmental assessment.

92. Keogh F, Carney P, O'Shea E. Innovative methods for involving people with dementia and carers in the policymaking process. Health Expect Int J Public Particip Health Care Health Policy. 2021;24:800–9.

93. Timotijevic L, Raats MM. Evaluation of two methods of deliberative participation of older people in food-policy development. Health Policy Amst Neth. 2007;82:302–19.

94. Van Loon AF, Lester-Moseley I, Rohse M, Jones P, Day R. Creative practice as a tool to build resilience to natural hazards in the Global South. Geosci Commun. 2020;3:453–74.

95. Redman-MacLaren M, Mills J, Tommbe R. Interpretive focus groups: a participatory method for interpreting and extending secondary analysis of qualitative data. Glob Health Action. 2014;7:25214.

96. Segal-Engelchin D, Huss E, Massry N. Arts-Based Methodology for Knowledge Co-Production in Social Work. Br J Soc Work. 2020;50:1277–94.

97. Rockloff SF, Lockie S. Participatory tools for coastal zone management: Use of stakeholder analysis and social mapping in Australia. J Coast Conserv. 2004;10:81–92.

98. Blanc S, Lingua F, Bioglio L, Pensa RG, Brun F, Mosso A. Implementing Participatory Processes in Forestry Training Using Social Network Analysis Techniques. Forests. 2018;9:463.

99. Reed MS, Graves A, Dandy N, Posthumus H, Hubacek K, Morris J, et al. Who's in and why? A typology of stakeholder analysis methods for natural resource management. J Environ Manage. 2009;90:1933–49.

100. Forsyth A, Slotterback CS, Krizek KJ. Health impact assessment in planning: Development of the design for health HIA tools. Environ Impact Assess Rev. 2010;30:42–51.

101. Yates KL, Schoeman DS. Spatial Access Priority Mapping (SAPM) with Fishers: A Quantitative GIS Method for Participatory Planning. PLOS ONE. 2013;8:e68424.

102. Learner Verification: A Methodology to Create Suitable Education Materials | HLRP: Health Literacy Research and Practice [Internet]. [cited 2024 Sep 28]. Available from: https://journals.healio.com/doi/10.3928/24748307-20210201-02

103. Nedelcu A. Analysing students' drawings of their classroom: A child-friendly research method. Rev Cercet Si Interv Sociala. 2013;42:275–93.

104. McCarthy L, Muthuri JN. Engaging Fringe Stakeholders in Business and Society Research: Applying Visual Participatory Research Methods. Bus Soc. 2018;57:131–73.

105. Barker J, Weller S. "Is it fun?" developing children centred research methods. Int J Sociol Soc Policy. 2003;23:33–58.

106. Pereira L, Hichert T, Hamann M, Preiser R, Biggs R. Using futures methods to create transformative spaces: visions of a good Anthropocene in southern Africa. Ecol Soc [Internet]. 2018 [cited 2024 Sep 28];23. Available from: https://www.ecologyandsociety.org/vol23/iss1/art19/

107. Townley G, Kloos B, Wright PA. Understanding the experience of place: Expanding methods to conceptualize and measure community integration of persons with serious mental illness. Health Place. 2009;15:520–31.

108. Forrester J, Cook B, Bracken L, Cinderby S, Donaldson A. Combining participatory mapping with Q-methodology to map stakeholder perceptions of complex environmental problems. Appl Geogr. 2015;56:199–208.

109. Wright PR, Wakholi PM. Festival as methodology: the African cultural youth arts festival. Mark Vicars and Dr Jon Austin D, editor. Qual Res J. 2015;15:213–27.

110. Blodgett AT, Coholic DA, Schinke RJ, McGannon KR, Peltier D, Pheasant C. Moving beyond words: exploring the use of an arts-based method in Aboriginal community sport research. Qual Res Sport Exerc Health. 2013;5:312–31.

111. Hemy AD, Meshulam A. 'Is that okay, teacher?' The camera as a tool to challenge power relations in a participatory action research classroom. Qual Res. 2021;21:750–67.

112. Bhanjee T, Mugabe M, Mawoneke S. Measuring HIV and AIDS Community Competence Using SAT's Competence Tool (SATCOMP): A Case Study of AIDS Among Us in Zimbabwe. Can J Public Health. 2008;99:S27–34.

113. Gerritsen S, Harré S, Rees D, Renker-Darby A, Bartos AE, Waterlander WE, et al. Community Group Model Building as a Method for Engaging Participants and Mobilising Action in Public Health. Int J Environ Res Public Health. 2020;17:3457.

114. Systems Thinking Tools as Applied to Community-Based Participatory Research: A Case Study - Rhonda BeLue, Chakema Carmack, Kyle R. Myers, Laurie Weinreb-Welch, Eugene J. Lengerich, 2012 [Internet]. [cited 2024 Sep 28]. Available from: https://journals.sagepub.com/doi/10.1177/1090198111430708

115. Leurs R. The role of the state in enabling private sector development: an assessment methodology. Public Adm Dev [Internet]. 2000 [cited 2024 Sep 10]; Available from: https://onlinelibrary.wiley.com/doi/10.1002/1099-162X(200002)20:1%3C43::AID-PAD110%3E3.0.CO;2-R

116. Green EP, Warren VR, Broverman S, Ogwang B, Puffer ES. Participatory mapping in low-resource settings: Three novel methods used to engage Kenyan youth and other community members in community-based HIV prevention research. Glob Public Health. 2016;11:583–99.

117. Nomakhwezi Mayaba N, Wood L. Using Drawings and Collages as Data Generation Methods With Children: Definitely Not Child's Play. Int J Qual Methods. 2015;14:1609406915621407.

118. Wagemakers A, Mulderij LS, Verkooijen KT, Groenewoud S, Koelen MA. Care-physical activity initiatives in the neighbourhood: study protocol for mixed-methods research on participation, effective elements, impact, and funding methods.

119. Bulmer SM, Barton BA, Liefeld J, Montauti S, Santos S, Richard M, et al. Using CBPR Methods in College Health Research: Exploring Excessive Alcohol Consumption.

120. The "Sticky Notes" Method: Adapting Interpretive Description Methodology for Team-Based Qualitative Analysis in Community-Based Participatory Research - Heather Burgess, Kate Jongbloed, Anna Vorobyova, Sean Grieve, Sharyle Lyndon, Tim Wesseling, Kate Salters, Robert S. Hogg, Surita Parashar, Margo E. Pearce, 2021 [Internet]. [cited 2024 Sep 28]. Available from: https://journals.sagepub.com/doi/10.1177/10497323211002489

121. Using mind mapping techniques for rapid qualitative data analysis in public participation processes - Burgess-Allen - 2010 - Health Expectations - Wiley Online Library [Internet]. [cited 2024 Sep 28]. Available from: https://onlinelibrary.wiley.com/doi/10.1111/j.1369-7625.2010.00594.x

122. Litovuo L, Karisalmi N, Aarikka-Stenroos L, Kaipio J. Comparing Three Methods to Capture Multidimensional Service Experience in Children's Health Care: Video Diaries, Narratives, and Semistructured Interviews. Int J Qual Methods. 2019;18:1609406919835112.

123. O'Hara L, Higgins K. Participant Photography as a Research Tool: Ethical Issues and Practical Implementation. Sociol Methods Res. 2019;48:369–99.

124. Lahtinen M, Nenonen S, Rasila H, Lehtelä J, Ruohomäki V, Reijula K. Rehabilitation Centers in Change: Participatory Methods for Managing Redesign and Renovation. HERD Health Environ Res Des J. 2014;7:57–75.

125. Best P, Badham J, Corepal R, O'Neill RF, Tully MA, Kee F, et al. Network methods to support user involvement in qualitative data analyses: an introduction to Participatory Theme Elicitation. Trials. 2017;18:559.

126. Pollock A, Campbell P, Baer G, Choo PL, Morris J, Forster A. User involvement in a Cochrane systematic review: using structured methods to enhance the clinical relevance, usefulness and usability of a systematic review update. Syst Rev. 2015;4:55.

127. Yonas MA, Burke JG, Miller E. Visual Voices: A Participatory Method for Engaging Adolescents in Research and Knowledge Transfer. Clin Transl Sci. 2013;6:72–7.

128. Dodds S, Bulmer S, Murphy A. Incorporating visual methods in longitudinal transformative service research. J Serv Theory Pract. 2018;28:434–57.

129. Pedell S, Keirnan A, Priday G, Miller T, Mendoza A, Lopez-Lorca A, et al. Methods for Supporting Older Users in Communicating Their Emotions at Different Phases of a Living Lab Project. Technol Innov Manag Rev. 2017;7:7–19.

130. Ospina-Pinillos L, Davenport TA, Ricci CS, Milton AC, Scott EM, Hickie IB. Developing a Mental Health eClinic to Improve Access to and Quality of Mental Health Care for Young People: Using Participatory Design as Research Methodologies. J Med Internet Res. 2018;20:e9716.

131. Kensing F, Sigurdardottir H, Stoop A. MUST--a participatory method for designing sustainable health IT. Stud Health Technol Inform. 2007;129:1204–8.

132. O'Brien N, Heaven B, Teal G, Evans EH, Cleland C, Moffatt S, et al. Integrating Evidence From Systematic Reviews, Qualitative Research, and Expert Knowledge Using Co-Design Techniques to Develop a Web-Based Intervention for People in the Retirement Transition. J Med Internet Res. 2016;18:e5790.

133. Cottafava D, Corazza L. Co-design of a stakeholders' ecosystem: an assessment methodology by linking social network analysis, stakeholder theory and participatory mapping.

134. Bird S, Wiles JL, Okalik L, Kilabuk J, Egeland GM. Methodological consideration of story telling in qualitative research involving Indigenous Peoples. Glob Health Promot. 2009;16:16–26.

135. Fraser SL. What stories to tell? A trilogy of methods used for knowledge exchange in a community-based participatory research project. Action Res. 2018;16:207–22.

136. Kohfeldt D. The Five Whys Method: A Tool for Developing Problem Definitions in Collaboration with Children. J Community Appl Soc Psychol [Internet]. 2012 [cited 2024 Sep 17]; Available from: https://onlinelibrary.wiley.com/doi/10.1002/casp.1114

137. Wang Q. Art-based narrative interviewing as a dual methodological process: A participatory research method and an approach to coaching in secondary education. Int Coach Psychol Rev. 2016;11:39–56.

138. Zorrilla P, Carmona G, De la Hera Á, Varela-Ortega C, Martínez-Santos P, Bromley J, et al. Evaluation of Bayesian Networks in Participatory Water Resources Management, Upper Guadiana Basin, Spain. Ecol Soc [Internet]. 2010 [cited 2023 Nov 22];15. Available from: https://www.jstor.org/stable/26268156

139. Maalim A d. Participatory rural appraisal techniques in disenfranchised communities: a Kenyan case study. Int Nurs Rev. 2006;53:178–88.

140. Trischler J, Scott DR. Designing Public Services: The usefulness of three service design methods for identifying user experiences. Public Manag Rev. 2016;18:718–39.

141. Treves A, Andriamampianina L, Didier K, Gibson J, Plumptre A, Wilkie D, et al. A Simple, Cost-Effective Method for Involving Stakeholders in Spatial Assessments of Threats to Biodiversity. Hum Dimens Wildl. 2006;11:43–54.

142. Wang Q. Art-based narrative interviewing as a dual methodological process: A participatory research method and an approach to coaching in secondary education. Int Coach Psychol Rev. 2016;11:39–56.

143. Schaper M-M, Iversen OS, Malinverni L, Pares N. FUBImethod: Strategies to engage children in the co-design of Full-Body interactive experiences. Int J Hum-Comput Stud. 2019;132:52–69.

144. Foster-Fishman PG, Law KM, Lichty LF, Aoun C. Youth ReACT for Social Change: A Method for Youth Participatory Action Research. Am J Community Psychol. 2010;46:67–83.

145. Cross-Sudworth F, Williams A, Herron-Marx S. Maternity services in multi-cultural Britain: Using Q methodology to explore the views of first- and second-generation women of Pakistani origin. Midwifery. 2011;27:458–68.

146. Barnidge E, Baker EA, Motton F, Rose F, Fitzgerald T. A participatory method to identify root determinants of health: the heart of the matter.

147. Moletsane R, de Lange N, Mitchell C, Stuart J, Buthelezi T, Taylor M. Photo-voice as a tool for analysis and activism in response to HIV and AIDS stigmatisation in a rural Kwazulu-Natal school.

148. Campbell DJT, Davidson MA. The challenges of managing diabetes while homeless: a qualitative study using photovoice methodology: CMAJ.

149. López EDS, Eng E, Randall-David E, Robinson N. Quality-of-Life Concerns of African American Breast Cancer Survivors Within Rural North Carolina: Blending the Techniques of Photovoice and Grounded Theory. Qual Health Res. 2005;15:99–115.

150. Scott-Bottoms S, Roe M. Who is a hydrocitizen? The use of dialogic arts methods as a research tool with water professionals in West Yorkshire, UK. Local Environ. 2020;25:273–89.

151. Nomakhwezi Mayaba N, Wood L. Using Drawings and Collages as Data Generation Methods With Children: Definitely Not Child's Play. Int J Qual Methods. 2015;14:1609406915621407.

152. Windsor LC. Using Concept Mapping in Community-Based Participatory Research: A Mixed Methods Approach. J Mix Methods Res. 2013;7:274–93.

153. Ravarotto L, Crovato S, Mantovani C, D'Este F, Pinto A, Mascarello G. Reducing microbiological risk in the kitchen: piloting consensus conference methodology as a communication strategy. J Risk Res. 2016;19:934–50.

154. Nyström A-G, Mustonen M, Yrjölä S. Co-Creating User Stories: A Tool for Making Sense of Business Opportunities.

155. Caretta MA, Vacchelli E. Re-Thinking the Boundaries of the Focus Group: A Reflexive Analysis on the Use and Legitimacy of Group Methodologies in Qualitative Research.
